# Ovule initiation in crops characterized by multi-ovulate ovaries

**DOI:** 10.1186/s43897-024-00116-0

**Published:** 2024-10-18

**Authors:** Yuan-Xin Wang, Xian-Chen Geng, Lu-Han Yang, Ze-Yu Xiong, Yu-Tong Jiang, Jian Pan, Wen-Hui Lin

**Affiliations:** 1 School of Life Sciences and Biotechnology, The Joint International Research Laboratory of Metabolic & Developmental Sciences, Shanghai Jiao Tong University, Shanghai, 200240 China; 2 Shanghai Collaborative Innovation Center of Agri‐Seeds/Joint Center for Single Cell Biology, Shanghai Jiao Tong University, Shanghai, 200240 China; 3 College of Horticulture, Shenyang Agricultural University, Shenyang, 110866 China

Seed is crucial for reproduction in angiosperms and serves as a significant food source for humans. Increasing seed number benefits both plant breeding and crop yield. Ovule is the precursor of seed. In plants characterized by multi-ovulate ovaries (producing many seeds per fruit), ovule initiation determines ovule number per flower and the maximum seed number per fruit, which is an important yield trait and has great impact in seed yield (Cucinotta et al. 2020). Fruit develops from the ovary, the main part of the pistil, which is formed by the inward coiling of one carpel or the union of several carpels. Carpels fuse to form the placenta, on which ovules protrude (Ferrandiz et al. 2010). Based on the number of carpels and their fusion position, angiosperm placentation can be classified as marginal, parietal, free-central, axial, apical, basal, and others (Shivaprakash and Bawa 2022). In the model plant *Arabidopsis thaliana*, the pistil comprises two carpels that fuse to form four carpel margin meristems, developing into four placentae. Ovules are linearly arranged on each placenta, and ovule primordia initiate asynchronously in the same placenta, including two processes: early initiation (in two rounds) and late initiation (Yu et al. 2022). Placenta elongation, the identity of ovule primordia and boundaries, and ovule primordia initiation determine ovule number. Numerous genes, such as *AINTEGUMENTA* (*ANT*) and *APETALA2* (*AP2*), are reported to be expressed in the placenta and ovule, regulating placenta development and ovule identity, thereby affecting ovule number. Multiple phytohormones, including auxin, cytokinin (CK), brassinosteroid (BR), and gibberellin acid (GA), are involved in regulating ovule initiation and ovule number by influencing gynoecium/placenta size and/or other mechanisms. Phytohormone-related genes (e.g., *PIN-FORMED 3* (*PIN3*), *CYTOKININ OXIDASE 3/5* (*CKX3/5*), *BRASSINAZOLE-RESISTANT 1* (*BZR1*), and *DELLAs*) also express in the placenta and ovule during ovule initiation (relevant references were listed in Supplementary Table 1).

To investigate whether conserved regulatory mechanisms of ovule initiation exist among plants with multi-ovulate ovaries, we selected four important crops: rapeseed (*Brassica napus*), cucumber (*Cucumis sativus*), soybean (*Glycine max*), and tomato (*Solanum lycopersicum*). The pattern formation of flowers and fruits was initially observed through dissection (Fig. 1A and Supplementary Fig. 1). Subsequently, the morphology of placenta and ovule primordia during ovule initiation was further examined using Differential Interference Contrast (DIC) (Fig. 1B). Rapeseed exhibits a morphology and pattern formation of placenta and ovule primordia similar to Arabidopsis, with a marginal placenta. The pistil contains four placentae, and ovules are linearly arranged on each placenta (Fig. 1B). Cucumber also possesses the marginal placenta, but its pistils consist of three carpels, forming six placentae. Each placenta has two or more rows of ovules, with the ovules linearly arranged in each row (Fig. 1B). The coexistence of ovules in different developmental stages within the same placenta of rapeseed and in the same row of the same placenta of cucumber was observed, indicating that rapeseed and cucumber follow similar rules of asynchronous ovule initiation as Arabidopsis. However, the spacing of young and old ovules in cucumber is not as neatly interval-arranged as in Arabidopsis, with many young ovules mainly located at the ends of the placenta (Fig. 1B, outlined in dashed lines), suggesting that the initiation pattern of ovule primordia in cucumber differs from that of Arabidopsis, likely due to the distinct growth pattern of the placenta. Soybean (Williams 28 variety) also has marginal placenta developed from a single carpel. The ovules are borne on both edges of the fused carpel, forming two rows in the pod. Most soybean pods contain 2 or 3 seeds and only 1 or 2 ovules on each row (Fig. 1B). Due to the small number of ovules in soybean, asynchronous initiation was not observed. Furthermore, soybean exhibits abundant placenta space, with adjacent ovules not tightly connected as in Arabidopsis, rapeseed, and cucumber.

**Fig. 1:**
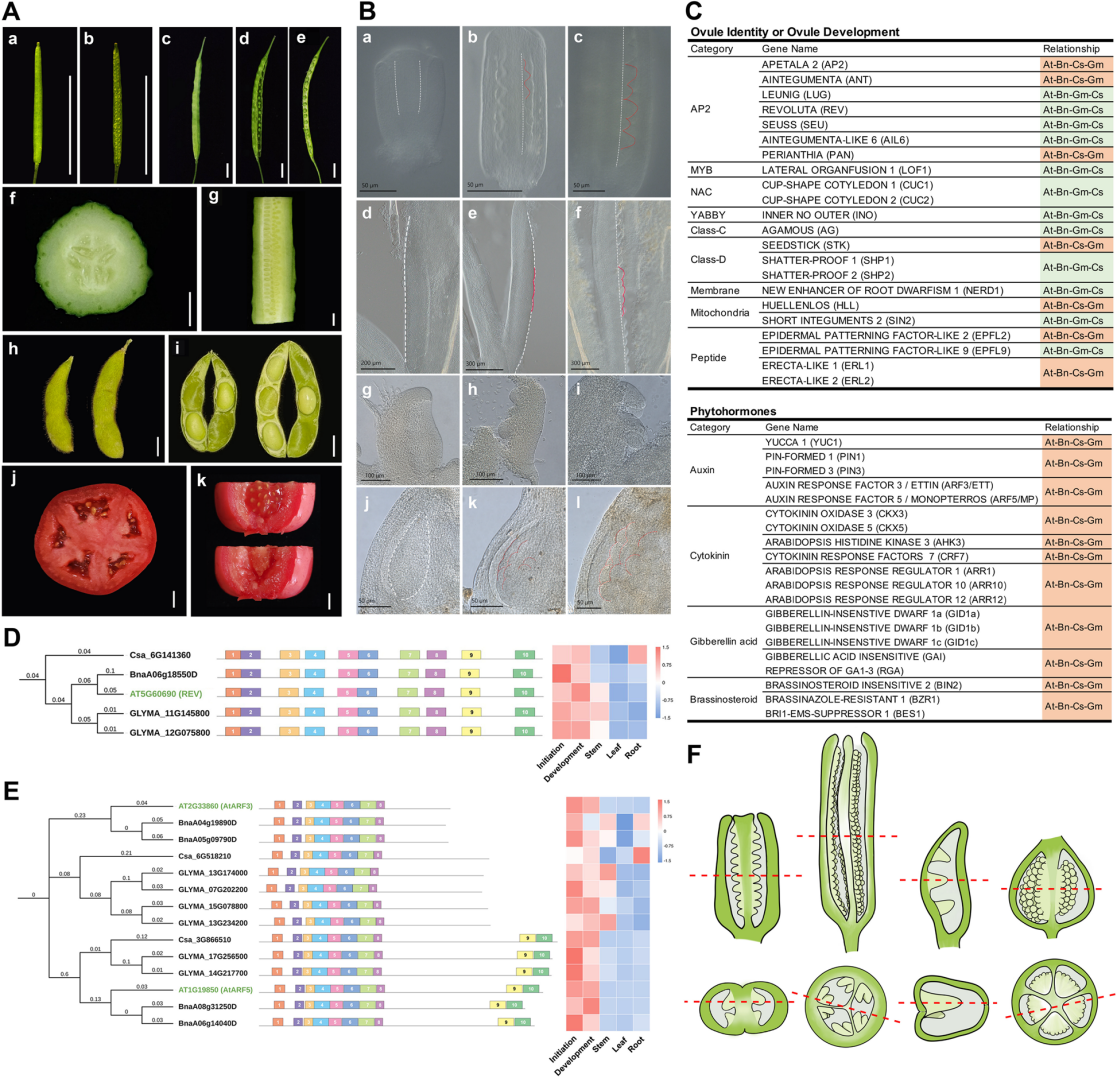
**A** Fruits anatomy of Arabidopsis (a and b), rapeseed (c to e), cucumber (f and g), soybean (h and i) and tomato (j and k) (Bars = 10 mm). **B** DIC observation of pistils of rapeseed, cucumber, soybean and tomato at different stages of ovule initiation and development. From left to right, the developmental stages range from early to late, with the white dashed line depicting the position of the placenta and the red line highlighting a few ovules that differ in developmental stages. For rapeseed, a. empty placenta without ovule primordia, which corresponds to Arabidopsis floral development stage 8. b. ovule primordia initiated, large and small ovules co-existed in the same placenta, suggesting the asynchronous initiation, which corresponds to Arabidopsis floral development stage 9b. c. large and small ovules arranged interval on one placenta, which corresponds to Arabidopsis floral development stage 9c (Bars = 100 μm). For cucumber, d. empty placenta before ovule initiation (Bar = 200 μm). e. earlier stage of ovule initiation, ovule primordia started to protrude from the placenta (Bar = 300 μm). f. large and small ovules coexisted in the same placenta, suggesting the asynchronous initiation (Bar = 300 μm). For soybean, g. empty placenta before ovule initiation. h. earlier stage of ovule initiation, ovule primordia protrude from the placenta. i. ovule primordia grow to finger shape (Bars = 100 μm). For tomato, j. empty placenta before ovule initiation. k. ovule primordia initiated from the placenta. l. ovule primordia growth (Bars = 100 μm). **C** The phylogenetic relationships of genes related to ovule initiation among four species. At, Bn, Cs, and Gm represent Arabidopsis, rapeseed, cucumber, and soybean, respectively. **D** The phylogenetic tree, conserved domains, and expression analysis of REVOLUTA (REV) proteins among Arabidopsis and three other species. Numbers within domains are sorted from left to right, and expression values are FPKM values, with elevated expression indicated from blue to red. **E** The phylogenetic tree, conserved domains, and expression analysis of AUXIN RESPONSE TRANSCRIPTION FACTOR 3/5 (ARF3/5) proteins among Arabidopsis and other three species. Numbers within domains are sorted from left to right, and expression levels are FPKM values, with elevated expression indicated from blue to red. **F** Models of ovule initiation in four crops. Transverse and vertical sections of pistils of rapeseed, cucumber, soybean and tomato from left to right. The red dotted line represents the cut position

Tomato exhibits an axial placentation (Brukhin et al. 2003). Its pistil consists of three to five carpels, which form three to five ventricles, each containing a placenta (Fig. 1A and Supplementary Fig. 1). The tomato placenta presents a curved surface, with numerous rows of ovules on each placenta. We observed that the ovules in each row are staggered in arrangement relative to the ovules in neighboring rows, illustrating an efficient utilization of ventricle space. Due to the presence of multiple rows of ovules on a single placenta, it is challenging to discern whether ovules at different developmental stages exist within each row, thus making it difficult to demonstrate asynchronous ovule initiation. Based on the aforementioned morphological observations, it is evident that the ovules of Arabidopsis, rapeseed, cucumber, and tomato are uniformly and densely arranged on each placenta or within each row of a placenta. This strategy maximizes the utilization of the space within the fruit, enabling the same-sized ovary to contain the maximum number of seeds.

In contrast to the four species mentioned above, the soybean pod exhibits considerable residual space on the placenta, suggesting that soybean may follow different rules of ovule initiation. Rapeseed and cucumber exhibit asynchronous ovule initiation similar to Arabidopsis, likely due to their shared type of placenta. Although soybean also possesses a linear placenta, we were unable to observe asynchronous ovule initiation due to the presence of fewer ovules. The pattern of placentation in tomato has undergone significant diversification from the other three species over the course of evolution. However, we hypothesize that ovule initiation in tomato likely shares some similar features with other species, enabling tomato to efficiently utilize ovary space and produce a greater number of ovules.

To further investigate the shared regulatory mechanisms of ovule initiation in rapeseed, cucumber, and soybean, which exhibit placenta patterns similar to Arabidopsis, we identified the corresponding homologous genes involved in ovule initiation in these three species (Supplementary Table 1) using the coding sequences from Arabidopsis as queries (Yu et al. 2022). We constructed phylogenetic trees based on the amino acid sequences of the homologous genes in the four species and analyzed their identities (Fig. 1C-E, and Supplementary Tables 1–3). The analysis of identities revealed that all homologous genes in rapeseed were the closest relatives to those in Arabidopsis. However, the identity of homologous genes in cucumber and soybean showed differential relationships to Arabidopsis. The genes regulating ovule initiation are primarily categorized into two groups: most genes directly affecting ovule identity or placenta development (*AGAMOUS* (*AG*), *PERIANTHIA* (*PAN*), *REVOLUTA* (*REV*), *LATERAL ORGAN FUSION 1* (*LOF1*), *CUP-SHAPED COTYLEDON 1/2* (*CUC1/2*), *ERECTA-LIKE 1/2* (*ERL1/2*), etc.) followed the "Arabidopsis-rapeseed-soybean-cucumber" relationship, while all phytohormone-related genes influencing placenta size or ovule number (*YUCCA 1* (*YUC1*), *AUXIN RESPONSE FACTOR 3/5* (*ARF3/5*), *BRASSINOSTEROID-INSENSITIVE 2* (*BIN2*), etc.) exhibited the "Arabidopsis-rapeseed-cucumber-soybean" relationship (Fig. 1C and Supplementary Figs. 2–3 and Table 1). The fact that a few ovule identity and development genes illustrated the same relationship as phytohormone-related genes might be attributed to their additional role in regulating placenta size (*ANT*, *SEEDSTICK* (*STK*), etc.). Our findings (Supplementary Fig. 4A) and previous studies (Bartrina et al. 2011) indicate a positive correlation between ovule number and placenta size. For example, phytohormones such as CK and BR can positively regulate ovule number by increasing placenta size (Supplementary Fig. 4B and 4C; Bartrina et al. 2011).

Therefore, we performed RNA-Sequencing (RNA-Seq) analysis to detect the expression level of the aforementioned genes at the stage of ovule initiation and development in rapeseed, cucumber, and soybean. We compared the RNA-Seq data with our Arabidopsis RNA-Seq. Additionally, we compared the expression of these genes in different tissues of four species (RNA-Seq sources were described in the ‘Materials and Methods’). The RNA-Seq results demonstrated that most genes involved in ovule identity and initiation were specifically expressed in young buds during ovule initiation (Fig. 1D and Supplementary Fig. 2), while phytohormone-related genes were expressed not only in young buds during ovule initiation but also in other tissues during vegetative development (Fig. 1E and Supplementary Fig. 3). However, the expression of phytohormone-related genes during ovule initiation (Supplementary Figs. 2–3 and Table 2) indicates that phytohormones likely regulate ovule initiation directly. For instance, Arabidopsis PIN1 is expressed in the placenta and ovule primordia, and enhanced BR signal increases PIN1 level and promotes ovule initiation (Supplementary Fig. 5). Some phytohormone-related genes in these crops (such as *BnCKX3*/5, *CsPIN1*, and *SlARF8A*/*B*) have also been reported to be expressed and function in the placenta (Supplementary Table 1; Hua et al. 2024). It is reasonable to hypothesize that phytohormones regulate cell division and meristematic tissue expansion, thereby affecting placenta size and ovule number (Bartrina et al. 2011; Galbiati et al. 2013). Arabidopsis, rapeseed, and cucumber have closely spaced ovules on the placenta and fully utilize the placental area. Enhanced placenta size increases ovule number (Supplementary Fig. 4A). However, in soybean, only a few ovules are sparsely arranged on the placenta, indicating that placenta size might not be a key factor limiting ovule number in this species. Therefore, we hypothesize that this may explain why phytohormone-related genes in soybean are evolutionarily distant from the remaining three species. Previous research has reported that the evolutionary trend of the fruit is towards increased fleshiness, where the seeds do not occupy all the space within the fruit and can have more space to store nutrients (Zhang and Ma 2024). The correlation between ovary space, placenta formation, and ovule number warrants further investigation.

Some mechanisms regulating ovule initiation by phytohormones are conserved between Arabidopsis and other species. CK significantly increases ovule number in both Arabidopsis and rapeseed. Similarly, auxin positively regulates ovule initiation in Arabidopsis and cucumber (Supplementary Table 1). BR promotes ovule initiation and increases ovule number in Arabidopsis and tomato (Supplementary Fig. 4B and 4C; Huang et al. 2013; Barro-Trastoy et al. 2020). However, there are a few differences in the regulation of phytohormone networks between Arabidopsis and tomato. In Arabidopsis, GA regulates ovule number independently of BR. In contrast, in tomato, BRs regulate ovule number by down-regulating GA biosynthesis (Barro-Trastoy et al. 2020).

In summary, based on the observation of ovule primordia initiation and analysis of the similarity of genes related to ovule initiation in Arabidopsis, rapeseed, cucumber, soybean, and tomato, we propose that asynchronous ovule initiation may be a common mode in plants with multi-ovule ovaries, such as those in the Cruciferae, Cucurbitaceae, Leguminosae, and even Solanaceae families. We hypothesize that ovule initiation in several rounds may be due to the continuous growth of the placenta. The total ovule number is determined by genetic information and environmental conditions, which are readily reflected by placenta area. In some plants characterized by multi‐ovulate ovaries with closely spaced ovules, such as Arabidopsis, rapeseed, and cucumber, increasing placenta size can effectively increase the number of ovules and seeds. Phytohormone-related genes may be involved in ovule number regulation by influencing placenta size and other mechanisms. Collectively, our study elucidates the conserved mechanisms of ovule initiation and ovule number regulation among crops characterized by multi-ovulate ovaries and provides insights for improving crop yield.

## Supplementary Information

Additional file 1. Materials and Methods.

Additional file 2: Supplementary Fig. 1. Inflorescence disassembly and flower anatomy of rapeseed, cucumber, soybean and tomato. The inflorescence disassembly and flower anatomy of rapeseed, cucumber (female flower), soybean and tomato are shown from top to bottom. Anatomy of rapeseed flower, with 4 petals, 4 sepals, 6 stamens and 1 pistil. The pistil is long and the ovary superior, similar to Arabidopsis. Anatomy of flower of cucumber gynoecy with 5-lobed corolla and inferior ovary. Anatomy of soybean flowers, bilaterally symmetrical, with 5 petals, 1 pistil and 10 stamens. Anatomy of tomato flower with sepals and corolla that have 5 lobes each but united around them, and with the stamens united with each other to enclose the pistil, which is long, and with a sub globular ovary (Bars = 10 mm). Supplementary Fig. 2. The expression patterns of genes involving in ovule identity and development in Arabidopsis and their homologues in other three species. The expression levels are FPKM values, with elevated expression indicated from blue to red. Supplementary Fig. 3. The expression patterns of phytohormones-related genes which regulating ovule initiation and ovule number in Arabidopsis and their homologues in other three species. The expression levels are FPKM values, with elevated expression indicated from blue to red. Supplementary Fig. 4. Ovule numbers and placenta length in wild type (WT) and BR-relevant mutants. (A) Ovule number is relevant to placenta length. The X axis represents different developmental stages of Arabidopsis WT flowers. The Y axises of the left and right side correspond to the placenta length and ovule number, respectively. (*n* = 10). (B) The placenta length of Arabidopsis *det2* (BR-deficient) and *bzr1-1D* (BR-signal-enhanced) mutants at floral stage 13 (*p* < 0.05, *n* = 10, one-way ANOVA, Tukey’s test). (C) The ovule number of Arabidopsis *det2* and *bzr1-1D* mutants at floral stage 13 (*p* < 0.05, *n* = 10, one-way ANOVA, Tukey’s test). Supplementary Fig. 5. BR signals promote ovule initiation by increasing the expression of PIN1. (A) The comparison of inflorescence between WT and *bzr1-1D* mutant (Bar = 2 mm). (B) PIN1 expression in placenta and ovule primordia and PIN1 protein level in WT and *bzr1-1D* mutant. (Bars = 30 μm).

Additional file 3: Supplementary Table 1. The genes involved in the regulation of ovule initiation and their homologs in three other species. Supplementary Table 2. Transcription profiles of genes related to ovule initiation in four species. Supplementary Table 3. Motif sequences.

Additional file 4. The sequences of genes related to ovule initiation in four species.

## Data Availability

The original data used to support the findings of this study are available from the corresponding author upon reasonable request. All data supporting the conclusions of this research are included within the article and its supplementary materials.
