# Supplementary material for: Ovule initiation in crops characterized by multi-ovulate ovaries
Source: Mol Hortic. 2024 Oct 18;4:39. doi: 10.1186/s43897-024-00116-0 (PMC11488239; doi:10.1186/s43897-024-00116-0)
Supplement: Supplementary file 1 — Additional file 1. Materials and Methods. [file 43897_2024_116_MOESM1_ESM.docx]

**Materials and Methods**

**Plant materials and growth conditions**

Arabidopsis (*Arabidopsis thaliana*) and rapeseed (*Brassica napus*) were grown in an environmentally controlled room (22℃, 16 h light/8 h dark photoperiod, 70% humidity). Soybean (*Glycine max* cv. William 82) were grown in an environmentally controlled room (28℃ 16 h light / 23℃ 8 h dark photoperiod, 70% humidity). Cucumber (*Cucumis sativus*) and tomato (*Solanum lycopersicum* cv. Micro-Tom) were grown in an environmentally controlled room (25℃, 16h light / 8h dark photoperiod, 70% humidity). The pistils of five species were respectively collected at ovule initiation and ovule development stages, and instantly frozen in the liquid nitrogen. The samples were stored at -80℃ for later RNA-Sequencing (RNA-Seq) analysis.

**Pistil observation**

For the four plants studied, pistils were dissected from fresh flowers around the time of ovule initiation under a stereoscope microscope. For DIC observations, pistils were fixed in 4% PFA for 1h, then cleared in ClearSee reagent for 1 week as described previously (Kurihara et al., 2015). Cleared pistils were observed under a microscope (Zeiss Axio Imager M2) with DIC optics.

**Fluorescent and Confocal Microscopy**

For confocal fluorescence microscopy, the observation were performed as described in previous study (Hu et al., 2022). The excitation and emission wavelengths were 488 nm and 500-560 nm, respectively. The fluorescence intensity was analyzed using the Image J software.

**Sequence identification and phylogenetic tree construction**

The genome sequences annotation files of Arabidopsis, rapeseed, cucumber and soybean were download from EnsemblPlants datasets (https://plants.ensembl.org/index.html/). The amino acid sequences of genes related to ovule initiation in Arabidopsis were downloaded from The Arabidopsis Information Resource (TAIR) database (https://www.arabidopsis.org/). These sequences were used as queries, the genomes of rapeseed, cucumber and soybean were blasted by the ‘Blast Zone’ plugin of TBtools with the default parameters. Phylogenetic trees of members in four species were constructed by using MEGA 7.0 software (http://www.megasoftware.net) with the Neighbor-Joining (NJ) method and 1000 bootstrap replicates. The visualization of output were performed by the online tool iTOL (https://itol.embl.de/tree/).

**Domain prediction**

The amino acid sequences of homologs are uploaded to the Multiple Em for Motif Elicitation (MEME) tool (https://meme-suite.org/meme/tools/meme/) for prediction of conserved domains between homologous sequences. The number of motifs for scanning is set to 10.

**RNA sequencing and Expression analysis**

RNA-sequencing of rapeseed, cucumber and soybean were performed in Novogene (Beijing, China). The FPKM value of pistils at different Arabidopsis flower stage were retrieved from our previous study (Yu et al., 2020). The fragment per kilobase of transcript, per million mapped reads (FPKM) value of leave, stem and root in Arabidopsis were downloaded from online tool Arabidopsis Electronic Fluorescent Pictograph (eFP) Brower (http://bar.utoronto.ca/efp/cgi-bin/efpWeb.cgi/) (Winter et al., 2007). The FPKM value of leave, stem and root in rapeseed were downloaded from BrassicaEDB database (https://brassica.biodb.org/) (Chao et al., 2020). The FPKM value of leave, stem and root in cucumber were obtained from previous study (Wei et al., 2016). The FPKM value of leave, stem and root in soybean were downloaded from Phytozome database (https://data.jgi.doe.gov/refine-download/phytozome/). The heatmaps were constructed for above data using R. Data in heatmaps were normalized for each row, and the default parameters were used.

**References**

Chao HY, Li T, Luo C, Huang H, Ruan Y, Li X, Niu Y, Fan Y, Sun W, Zhang K, Li J, Qu C, and Lu K. BrassicaEDB: a gene expression database for Brassica crops. Int. J. Mol. Sci. 2020; 21, 5831.

Hu LQ, Chang JH, Yu SX, Jiang YT, Li RH, Zheng JX, Zhang YJ, Xue HW, and Lin WH. *PIN3* positively regulates the late initiation of ovule primordia in *Arabidopsis* *thaliana*. Plos Genet. 2022; 18, e1010077. http://doi.org/10.1371/journal.pgen.1010077.

Kurihara D, Mizuta Y, Sato Y, and Higashiyama T. ClearSee: a rapid optical clearing reagent for whole-plant fluorescence imaging. Development 2015; 142, 4168-4179.

Wei G, Tian P, Zhang F, Qin H, Miao H, Chen Q, Hu Z, Cao L, Wang M, Gu X, Huang S, Chen M, Wang G. Integrative analyses of nontargeted volatile profiling and transcriptome data provide molecular insight into VOC diversity in cucumber plants (*Cucumis* *sativus*). Plant Physiol. 2016; 172, 603-618.

Winter D, Vinegar B, Nahal H, Ammar R, Wilson G, and Provart N. An “electronic fluorescent pictograph” browser for exploring and analyzing large-scale biological data sets. Plos One 2007; 2, e718.

Yu SX, Zhou LW, Hu LQ, Jiang YT, Zhang YJ, Feng SL, Jiao YL, Xu L, and Lin WH. Asynchrony of ovule primordia initiation in *Arabidopsis*. Development 2020; 147. http://doi.org/10.1242/dev.196618.
