# Supplementary figures and images for: Ovule initiation in crops characterized by multi-ovulate ovaries
Source: Mol Hortic. 2024 Oct 18;4:39. doi: 10.1186/s43897-024-00116-0 (PMC11488239; doi:10.1186/s43897-024-00116-0)

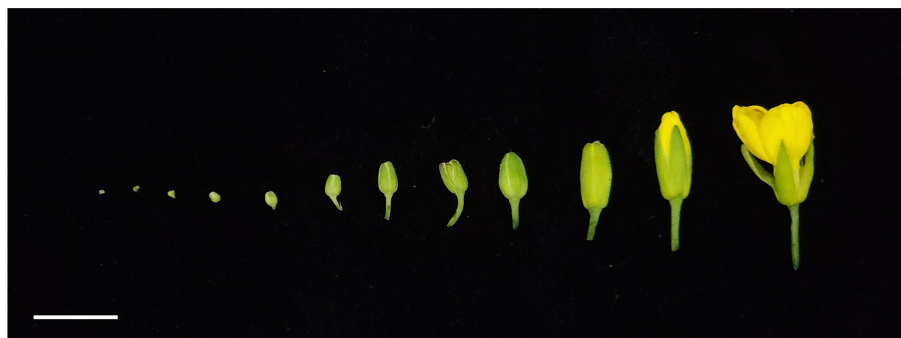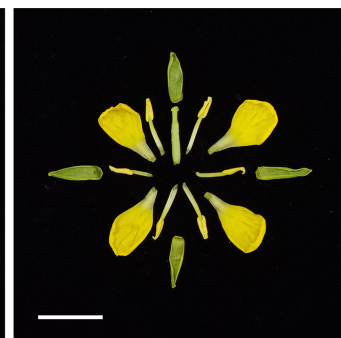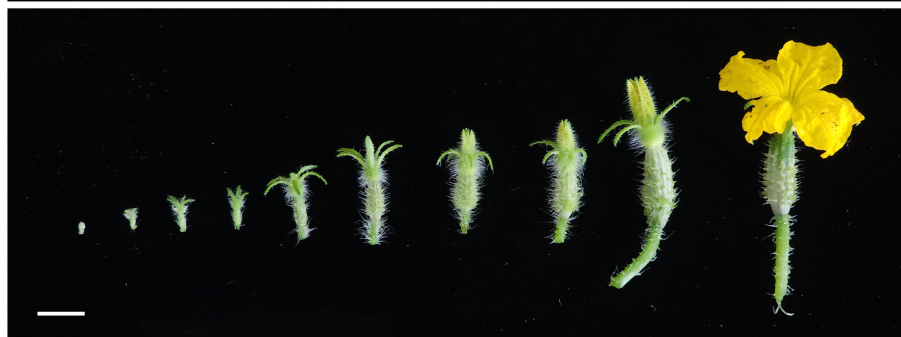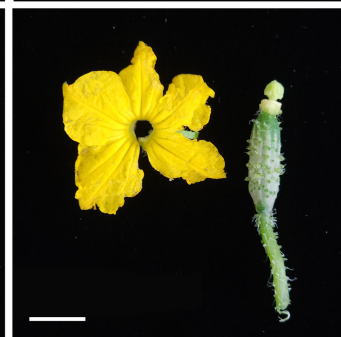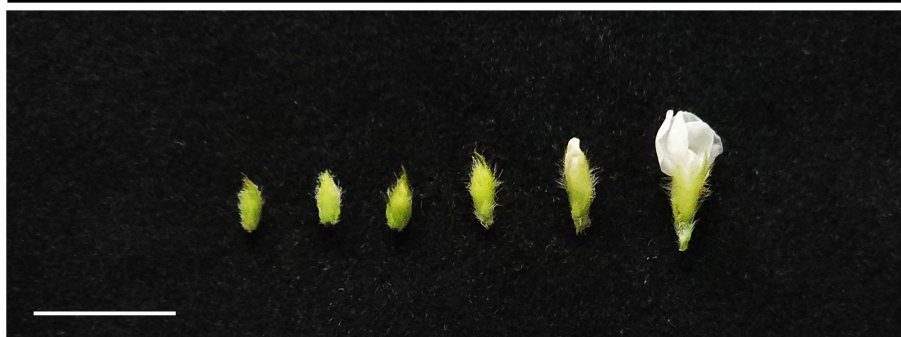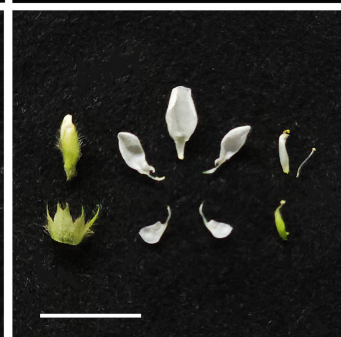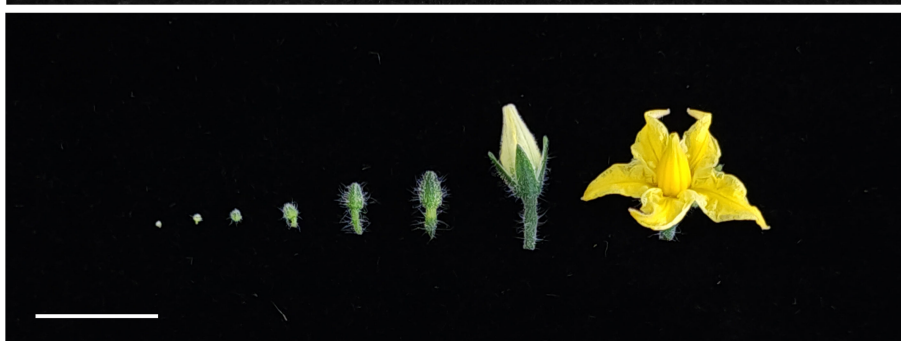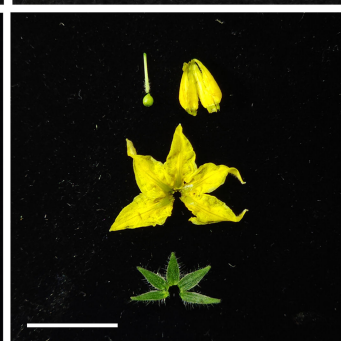

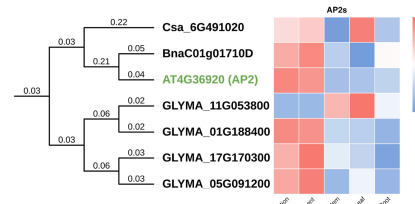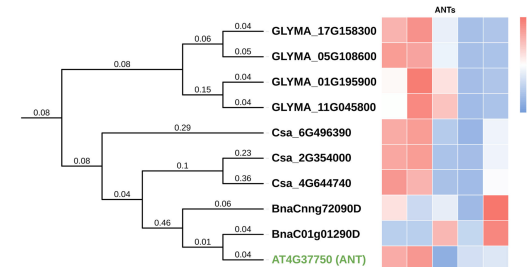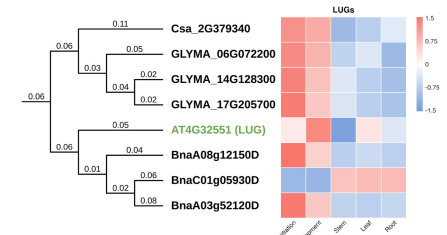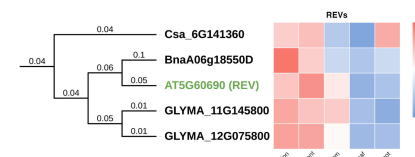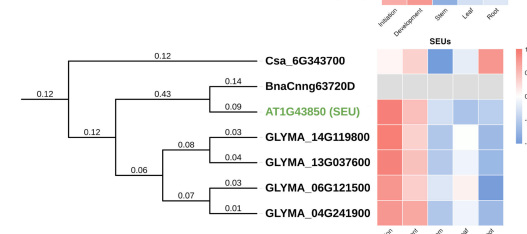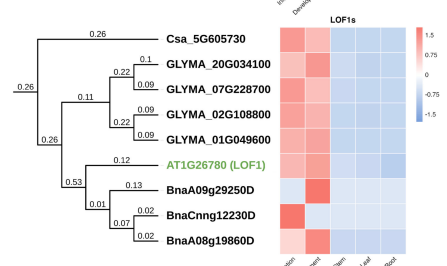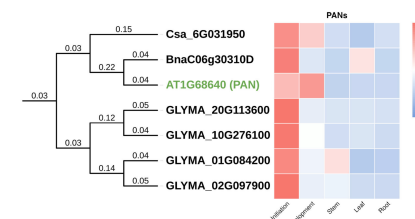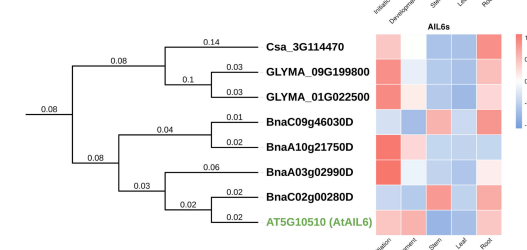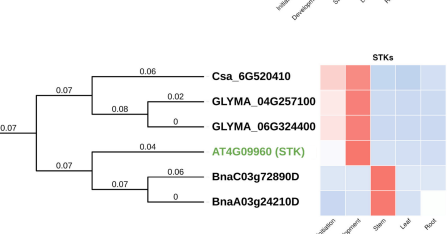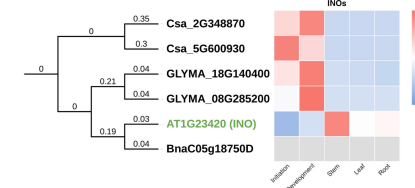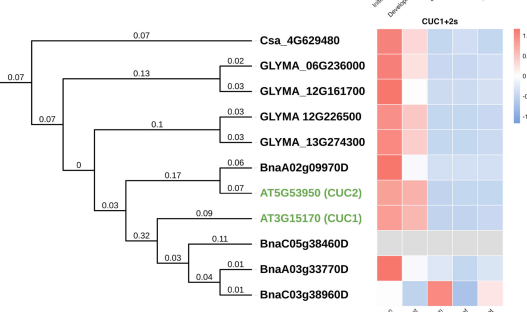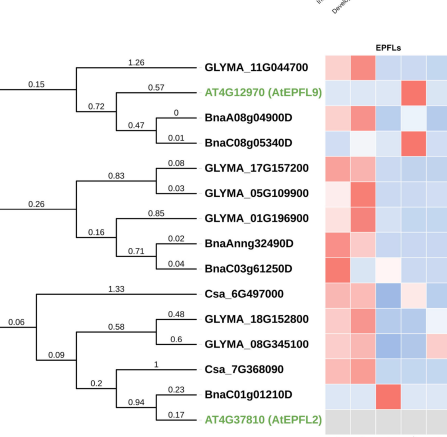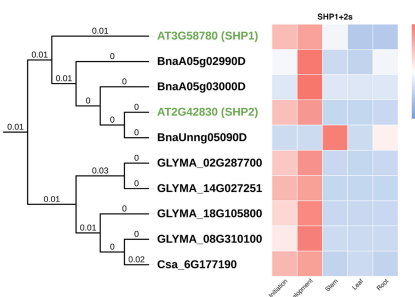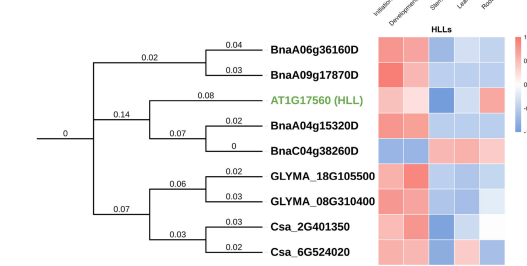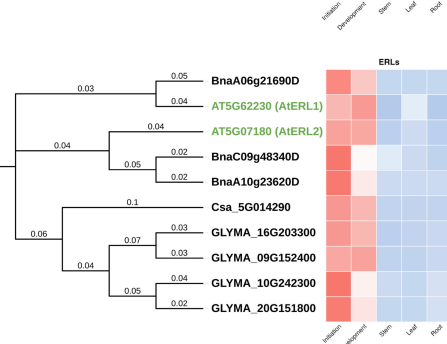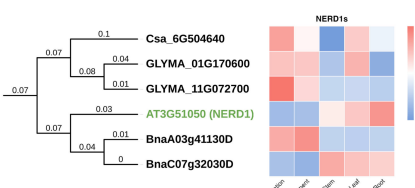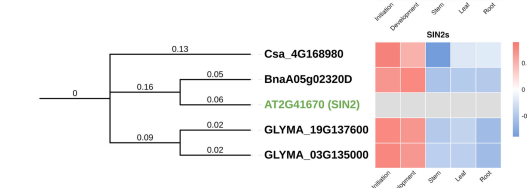

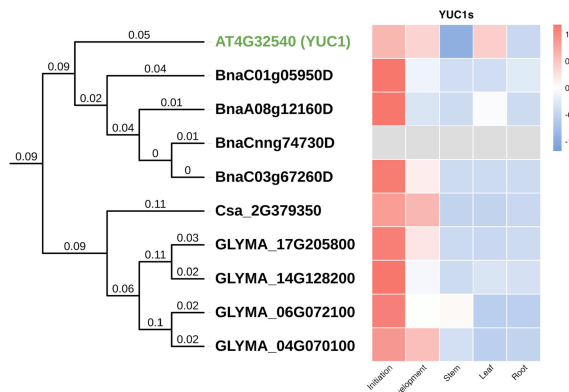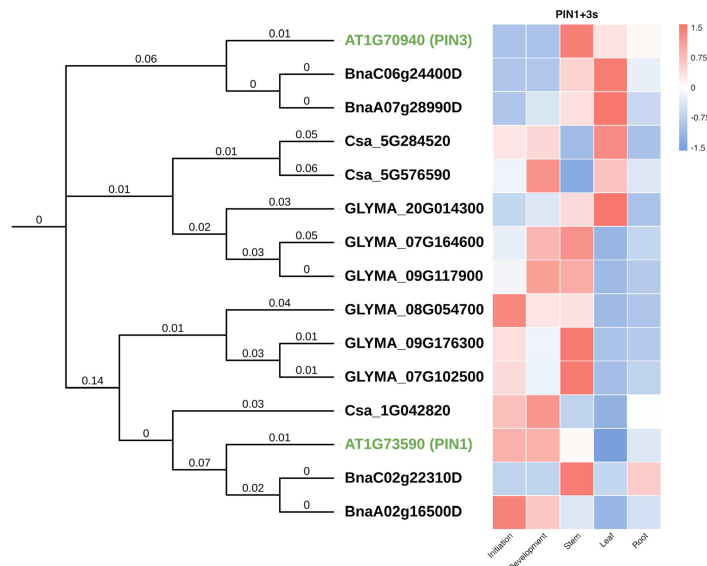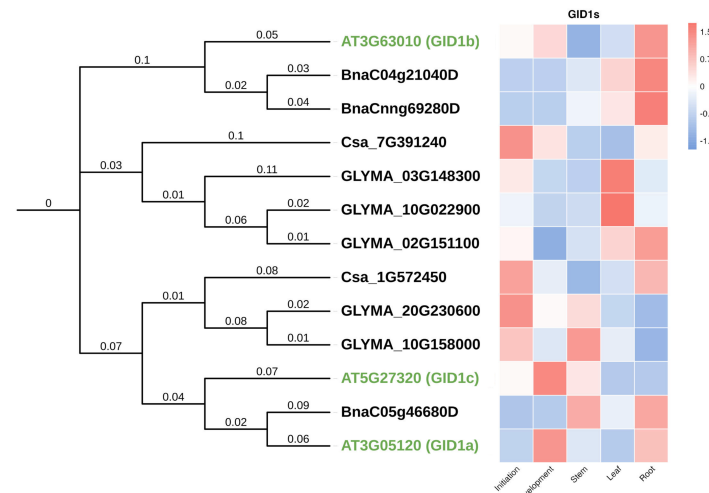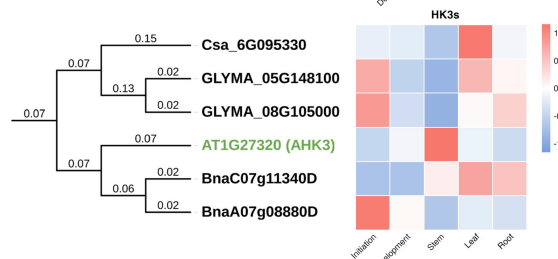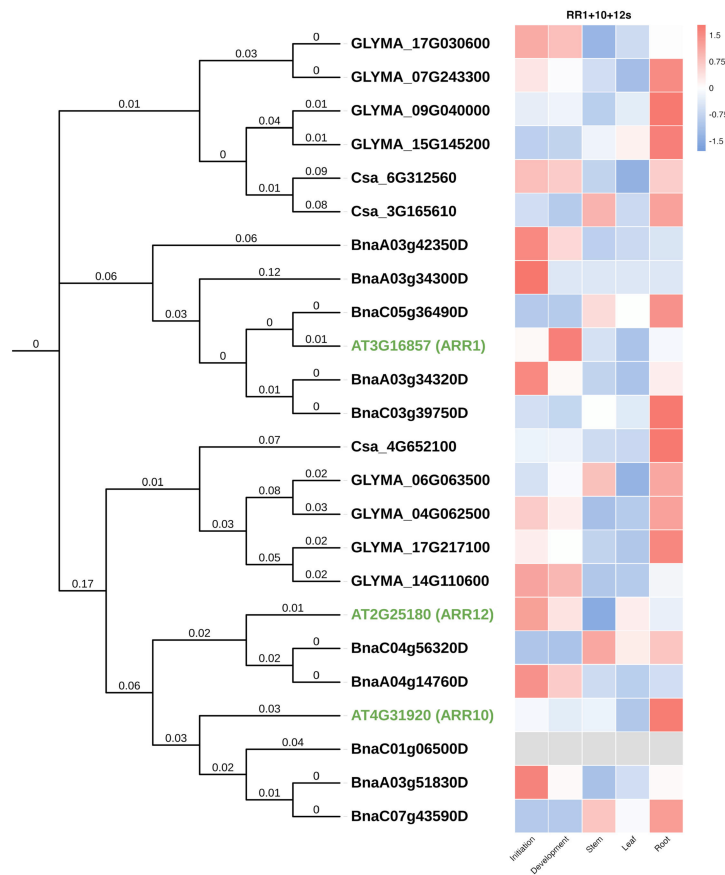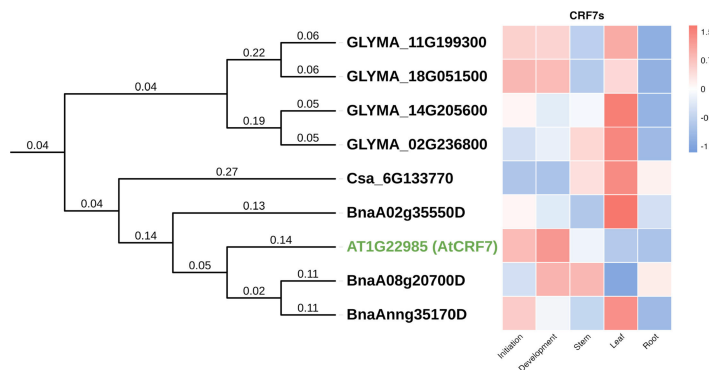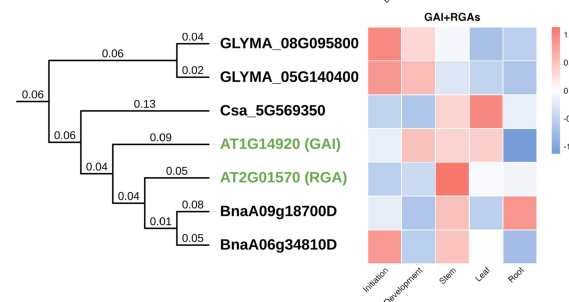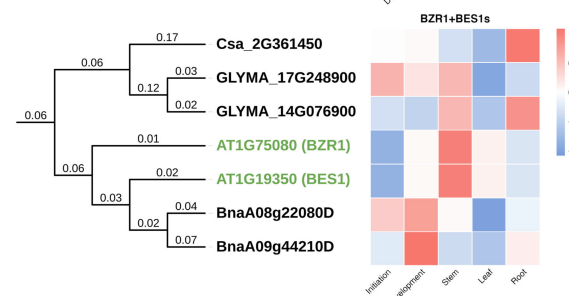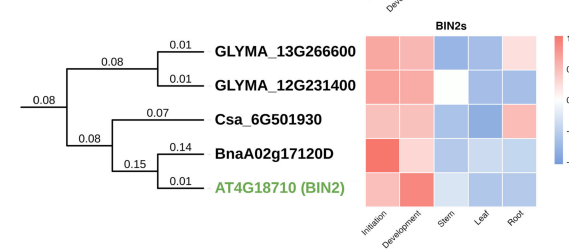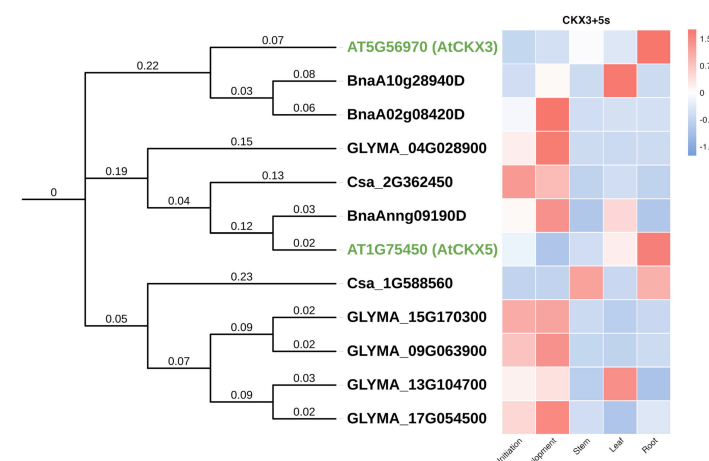

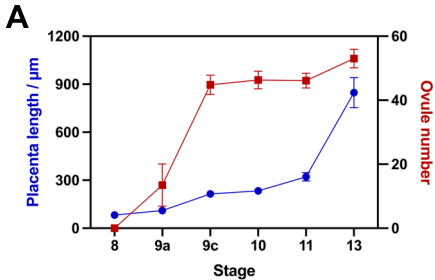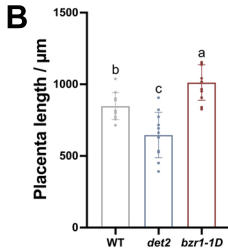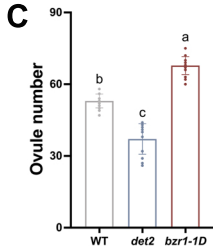

**A**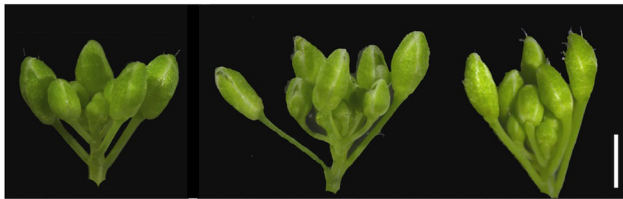**WT*****bzt1-1D*****B**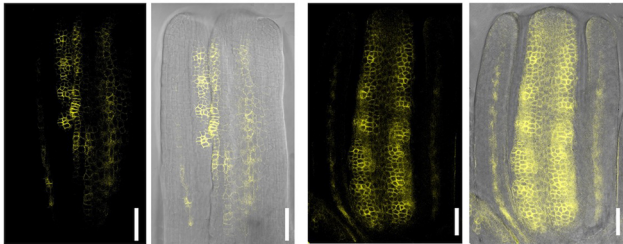**WT*****bzt1-1D******PIN1-YFP***

Supplement: Supplementary file 2 — Additional file 2: Supplementary Fig. 1. Inflorescence disassembly and flower anatomy of rapeseed, cucumber, soybean and tomato. The inflorescence disassembly and flower anatomy of rapeseed, cucumber (female flower), soybean and tomato are shown from top to bottom. Anatomy of rapeseed flower, with 4 petals, 4 sepals, 6 stamens and 1 pistil. The pistil is long and the ovary superior, similar to Arabidopsis. Anatomy of flower of cucumber gynoecy with 5-lobed corolla and inferior ovary. Anatomy of soybean flowers, bilaterally symmetrical, with 5 petals, 1 pistil and 10 stamens. Anatomy of tomato flower with sepals and corolla that have 5 lobes each but united around them, and with the stamens united with each other to enclose the pistil, which is long, and with a sub globular ovary (Bars = 10 mm). Supplementary Fig. 2. The expression patterns of genes involving in ovule identity and development in Arabidopsis and their homologues in other three species. The expression levels are FPKM values, with elevated expression indicated from blue to red. Supplementary Fig. 3. The expression patterns of phytohormones-related genes which regulating ovule initiation and ovule number in Arabidopsis and their homologues in other three species. The expression levels are FPKM values, with elevated expression indicated from blue to red. Supplementary Fig. 4. Ovule numbers and placenta length in wild type (WT) and BR-relevant mutants. (A) Ovule number is relevant to placenta length. The X axis represents different developmental stages of Arabidopsis WT flowers. The Y axises of the left and right side correspond to the placenta length and ovule number, respectively. (n = 10). (B) The placenta length of Arabidopsis det2 (BR-deficient) and bzr1-1D (BR-signal-enhanced) mutants at floral stage 13 (p < 0.05, n = 10, one-way ANOVA, Tukey’s test). (C) The ovule number of Arabidopsis det2 and bzr1-1D mutants at floral stage 13 (p < 0.05, n = 10, one-way ANOVA, Tukey’s test). Supplementary Fig. 5. [file 43897_2024_116_MOESM2_ESM.pdf]
