# Supplementary material for: Ovule initiation in crops characterized by multi-ovulate ovaries
Source: Mol Hortic. 2024 Oct 18;4:39. doi: 10.1186/s43897-024-00116-0 (PMC11488239; doi:10.1186/s43897-024-00116-0)
Supplement: Supplementary file 4 — Additional file 4. The sequences of genes related to ovule initiation in four species. [file 43897_2024_116_MOESM4_ESM.docx]

**The sequences of genes related to ovule initiation in four species**

**APETALA 2 (AP2)**

**>AT4G36920_AtAP2**

MWDLNDAPHQTQREEESEEFCYSSPSKRVGSFSNSSSSAVVIEDGSDDDELNRVRPNNPLVTHQFFPEMDSNGGGVASGFPRAHWFGVKFCQSDLATGSSAGKATNVAAAVVEPAQPLKKSRRGPRSRSSQYRGVTFYRRTGRWESHIWDCGKQVYLGGFDTAHAAARAYDRAAIKFRGVEADINFNIDDYDDDLKQMTNLTKEEFVHVLRRQSTGFPRGSSKYRGVTLHKCGRWEARMGQFLGKKYVYLGLFDTEVEAARAYDKAAIKCNGKDAVTNFDPSIYDEELNAESSGNPTTPQDHNLDLSLGNSANSKHKSQDMRLRMNQQQQDSLHSNEVLGLGQTGMLNHTPNSNHQFPGSSNIGSGGGFSLFPAAENHRFDGRASTNQVLTNAAASSGFSPHHHNQIFNSTSTPHQNWLQTNGFQPPLMRPS

**>Csa_6G491020**

MGCDAFSRLTKTSPPNKTKTKILLTQTSFPKGTRKKKMWDLNDWPDVREEDECSSAKTSIEGEGDEEKGKRVGSLSNSSSSAVVMEEEEAEVEGGSDEEEPTPMVTHQFFPLEETEIPTPLPHASAPPATAPAFPRAHWVGVKFAHPDPLAALPNNSLTPTDLSHPIKKSRRGPRSRSSQYRGVTFYRRTGRWESHIWDCGKQVYLGGFDTAHAAARAYDRAAIKFRGVEADINFSIEDYEDDLKQMGNLTKEEFVHVLRRQSTGFPRGSSKYRGVTLHKCGRWEARMGQFLGKKYVYLGLFDTEIEAARAYDKAAIKCNGKEAVTNFDPSIYENELNPTTESSSNLGDHSLDLSLGNSSSKQNDSSNNGSIGPQHHSSSSSSADWQRNHGFRPLQLNLENGGGGNKNYNNNVERRNRYLESETMQLLSQTHIQSPAQFSRPHTVGGAPDHHPHSQILLHNHNNQTFNYQIEFSGSSNGHGGRIVGSDLTLSLNDHLQSGPSQVLASAAASSGFAPQIRPSKNWLHNNGFHCLMRPS

**>BnaC01g01710D**

MWDLNDSPHQTLVEEESEELCYSSPGKRVGSFSNSSSSAVVIEDGSDDDEPNRVRPNNPLVTHQFFPEMETSVGDDGGGGPGSGFPRSHWFGVKFCQSDLATGSSAGKPATVAAVVEPAQPLKKSRRGPRSRSSQYRGVTFYRRTGRWESHIWDCGKQVYLGGFDTAHAAARAYDRAAIKFRGVEADINFTIEDYDDDLKQMTNLTKEEFVHVLRRQSTGFPRGSSKYRGVTLHKCGRWEARMGQFLGKKYVYLGLFDTEVEAARAYDKAAIKCNGKDAVTNFDPSIYDDELNAESSGNSIQQDHNLDLSLGNSVNSKQKGQDMRLKMNQQETLHPNEILGLGQTGMVNHIPNSNLQFPGSSNIGGGGGFSLFPVAENHRFDGRTTTNQVLANAAFFFFG

**>GLYMA_05G091200**

MWDLNDSPDQRINKDEESEEGCSSLNKTSFDGDDDNNNNKGKRVGSVSNSSSSAVVIGDGSEEEYEEEEEDEEGVGVGGGRSMKKRSSKIFGFSVTQNEEESMDSDHPPVTRQFFPVEDADVAVATGGGGTGVSSTFPRAHWVGVKFCQSETLGTGKSSVEVSQPMKKSRRGPRSRSSQYRGVTFYRRTGRWESHIWDCGKQVYLGGFDTAHAAARAYDRAAIKFRGVEADINFNIEDYEDDLKQMSNLTKEEFVHVLRRQSTGFPRGSSKYRGVTLHKCGRWEARMGQFLGKKYVYLGLFDTEIEAARAYDKAAIKCNGKEAVTNFDPSIYDNELNSAESTGNAPDHNLDLSLGNSTSNPGNDQALGNQAPNAVTHDQHMPSESNWRNGGIKPKLVNILPKPCYRSNNKDTHGRDVHGESETLRMLSQTHLHSPAFNEMQQRYGPYRSPGGESQMLQNFAHIHPPNFHFPRSSIGGRIGSDLSLSVADQQQWQTGPSRYLATAAASSSFQQEIRPSSQGWLQKNGLHSLMRPS

**>GLYMA_17G170300**

MWDLNDSPDQRINKDEGSEEGCSSLKTSIDGDDMINNNKGKRVGSVSNSSSSAVVIEDGSEEEVDEEEEEEEDDEEGGVGRSMKKRSSKIFGFSVTQDEESMDSDHPPVTRQFFPVEDADVAVATGGGTGGSSTFPRAHWVGVKFCQSETLGAGKSSVEVSQPMKKSRRGPRSRSSQYRGVTFYRRTGRWESHIWDCGKQVYLGGFDTAHAAARAYDRAAIKFRGVEADINFNIEDYEDDLKQMSNLTKEEFVHVLRRQSTGFPRGSSKYRGVTLHKCGRWEARMGQFLGKKYVYLGLFDTEIEAARAYDKAAIKCNGKEAVTNFDPSIYNNELNTAESTGNAPDHNLDLSLGNATSKPGNNQALGNHATNAVTHDQHLPSESNWRNGGNKPKLVNILPKPCNRSNNKDSHGRDVHGESETLRMLSQTHLHSPASNEMQLRYGPYKSHGGESQMLQNFAHIHPPNFHFPSSSIGGRIGSDLSLSMTDQQQWQTGPSHYLATAAASSSFQQQIRPSSQGWLQKNGFHILMRPS

**>GLYMA_01G188400**

MWDLNDSPDQRKDYESEGCSSSLYDDKGKRVASVSNSSSSAVVVEDGSEEEDSERGGSRTLDNKKTNKIFGFSVAHDDSDHPPVTHQFFPVEDSELPVTAAAAAAGSSFPRAHWVGVKFCQSETPGAGKAVKVSEPMKKSRRGPRSRSSQYRGVTFYRRTGRWESHIWDCGKQVYLGGFDTAHAAARAYDRAAIKFRGVEADINFNIEDYEEDLKQMTNLTKEEFVHVLRRQSTGFPRGSSKYRGVTLHKCGRWEARMGQFLGKKYVYLGLFDTEIEAARAYDKAAIKCNGKEAVTNFDPSIYDGELNSESSGGVAADHNLDLSLGNLISKHSNSQSSRNHFPNSATDQHMPPESNWQSGGSKPKVHLVNILPKPCGRSNMEAYGQGESETLRLLSQTHLQSPTTKEMHRYGPYRSPAEPQMPHNFAHHLHQPSFHVPSSSSNRGRIGSDLSLSMSDQQQWQQAGPPHLLATAAASSGFPQQIRPSQGWLQKNGFHSLMRPS

**>GLYMA_11G053800**

MWNLNDSPDQRKDYESEGCSSSLNDDKGKRVGSASNSSSSAVVVEDGSEEEDSERGGSRTLDKKTNKIFGFAVAHDDSDHPPVTHQFFPVEDSELPVSAAAAGSGGGGGGSSFPRAHWVGVKFCQSETPGAGKAVKVSEPMKKSRRGPRSRSSQYRGVTFYRRTGRWESHIWDCGKQVYLGGFDTAHAAARAYDRAAIKFRGVEADINFNIEDYEEDLKQMTNLTKEEFVHVLRRQSTGFPRGSSKYRGVTLHKCGRWEARMGQFLGKKYVYLGLFDTEIEAARAYDKAAIKCNGKEAVTNFDPSIYDSELNSESSGGVAADHNLDLSLGNSSSKHNNSQTSGNHFANSATDQHMPPESNWRNGGSKPKVHLVNILPKPCGRSNIEAYGHGESETLRLLSQTHLQSPTNKEMHRYGPYRSPAEPQKPHNFAHHLHQPSFHVPSSSSNGGRIGSDLSLSMSDQQQWQAGPPHLLATAAASSGFPQQIRPSQGWLQKNGFHSLMRPS

**AINTEGUMENTA (ANT)**

**>AT4G37750 (AtANT)**

MKSFCDNDDNNHSNTTNLLGFSLSSNMMKMGGRGGREAIYSSSTSSAATSSSSVPPQLVVGDNTSNFGVCYGSNPNGGIYSHMSVMPLRSDGSLCLMEALNRSSHSNHHQDSSPKVEDFFGTHHNNTSHKEAMDLSLDSLFYNTTHEPNTTTNFQEFFSFPQTRNHEEETRNYGNDPSLTHGGSFNVGVYGEFQQSLSLSMSPGSQSSCITGSHHHQQNQNQNHQSQNHQQISEALVETSVGFETTTMAAAKKKRGQEDVVVVGQKQIVHRKSIDTFGQRTSQYRGVTRHRWTGRYEAHLWDNSFKKEGHSRKGRQVYLGGYDMEEKAARAYDLAALKYWGPSTHTNFSAENYQKEIEDMKNMTRQEYVAHLRRKSSGFSRGASIYRGVTRHHQHGRWQARIGRVAGNKDLYLGTFGTQEEAAEAYDVAAIKFRGTNAVTNFDITRYDVDRIMSSNTLLSGELARRNNNSIVVRNTEDQTALNAVVEGGSNKEVSTPERLLSFPAIFALPQVNQKMFGSNMGGNMSPWTSNPNAELKTVALTLPQMPVFAAWADS

**>BnaC01g01290D**

MKSFCDNDDSNTTNLLGFSLSSNMLKMGGGEALYSSSSSSAATSSVPPQLVVGDNSSNYGVCYGSNSAAREIYSQREMYSQMSVMPLRSDGSLCLMEALNRSSHSNHHHHTQVSSPKMEDFFGTHHHNTSHKEAMDLSLDSLFYNTTHSPNNNTNFQEFFSFPQTRNHHEEETRNYENDPGLTHGGRSFNVGVYGEFQQSLSLSMSPGSQSSCITASHHHQNQNQTQNHHQISEALVETSAGFETTTMAAASKKKRGQEVVVGQKQLVHRKSIDTFGQRTSQYRGVTRHRWTGRYEAHLWDNSFKKEGHSRKGRQVYLGGYDMEEKAARAYDLAALKYWGPSTHTNFSVENYQKEIDDMKNMTRQEYVAHLRRKSSGFSRGASIYRGVTRHHQHGRWQARIGRVAGNKDLYLGTFGTQEEAAEAYDVAAIKFRGTNAVTNFDITRYDVDRIMASNTLLSGEMARRNSNSIVVRNISDEETALTAVVDGGSTKEVGSPERVLSFPTIFALPQVGPKMFGANVVGNMSSWTTNPNADLKTVSLTLPQMPVFAAWADS

**>BnaCnng72090D**

MKSFCDNDDNNHGNTTNLLGFSLSSNMLKMGGGGGEEALYSSSSSAATSSSVPPQLVVGDNSSNYGVCFGSNSAAGGMYSQMSVMPLRSDGSLCLMEALNRSSHSNQHHHTQVSSPKMEDFFGTHQNNTSNKEAMDLSLDSLFYNTTHEPNNNTNFQEFFSFPQARNHHEEETRSYQNDPGLTHGGGSFNVGVFGEFQQSLSLSMSPGSQSSCITGTHHHQNQNHQAQNHHQISEALGVETSVGFETTTMAAAAKKKRGQEEVMVVGQKQIVHRKSIDTFGQRTSQYRGVTRHRWTGRYEAHLWDNSFKKEGHSRKGRQGLEHYLF

**>Csa_6G496390**

MNNVNGNNCNWLGFSVSPNVNMELSSSAATSVSPSIPANLFHSPSQFNYGICYGVDGEHGAFYSPLSAMPLKSDGSICSMEALSRQHPQVVSSSTPKLEDFFGGATMGSHHYESNDREAMALSLDSIFCHQNPTHEPNNQSFAHFSSLRSRELMLQDSKVILPDGCNLQQQQQHPGVAQSDISGMKNWTVPRNYAATNNGSFEQKMVSCMSENGGESGSINAMAYGDLQSLSLSMTMSPSSQSSCVTATQHVSPAMTDCSAMDTKKRGHEKVDQKQIVHRKSLDTFGQRTSQYRGVTRHRWTGRYEAHLWDNSCKKEGQSRKGRQVYLGGYDMEEKAARAYDLAALKYWGPSTHINFPLENYQKELEEMKNMSRQEYVAHLRRRSSGFSRGASIYRGVTRSNKSQLKALHHQHGRWQARIGRVAGNKDLYLGTFSTQEEAAEAYDIAAIKFRGMNAVTNFDITRYDVERIIASNTLLSGDLAKRKQQPEFDNESLRQSPPTHNSNSEAMTLPSQSSSQSESDWKMALYHSSQQLIPKPRMLSAINDDGSQLGVEDSARMGAHFSNASSMVTSCSLSSSREESPDKTSLSMVFGMPQSTSKPFATSANNMNTSWIASAQQIRAANCMSQLPVFAAWTDT

**>Csa_4G644740**

MAMEASSEPQQHSHYHHRGQVTSAVAVSNTMSNSFLASHHYNGDFLSVVPLKSDGSLCIMDSINTSQPQDILPNISPKLEDFLGGATHGKETTALSLDSIYYDQNAEVGSERQHSLNLLNQQEQQQHILFHSQRYYSAMYQYPAENPDNESHIASYCSEIRQDMEQRINGCCFGGMNCQDLKPLSLSMSPGSQSSCVTTPSQISQPGPSTMEIKKRALASQKQPVHRKSIDTFGQRTSQYRGVTRHRWTGRYEAHLWDNSCKKEGQTRKGRQVYLGGYDMEEKAARAYDLAALKYWGSSTHLNFPLKNYELEIEEMKNMNRQEYVAHLRRKSSGFSRGASIYRGVTRHHQHGRWQARIGRVAGNKDLYLGTFGTQEEAAEAYDIAAIKFRGANAVTNFDTSRYDVERIIASSSLLSGEFARRKKEHKPTNTIERKEPKQNVTQTDEGLEMSTNLDWRAVFHDNLLLNPSASVESIDQKSMTSSRYVNHVIGVVETESSNQETVNDSRKYKTHFSNASSVVSSLSSSRETSPDKSNGSSSVLFAKSPFGSNGSNWLPSPQMRLAPISLPVWNDA

**>Csa_2G354000**

MKSMSDNEDSNNNNNNNNNSWLGFSLSPHMKMEVSSSDHPYNQHHSLHSASNPFYLSPHFNNNNTEIFYGIPDNSSLHHHSAAASLSVMPLKSDGSLCIMEALSRSQTEGMVPSSSPKLEDFLGGATMGGRGGYFNQNAESESDREHSFDLLQRPIRQNQQILIQNSNQYYSGLLPSSIGIGTCDPQILPPDDDGIPCFRNWVSRSHYSATHNTLEHHITGGDGGGGGTLMNESNGGGSASIGGMSCGELQSLSLSMSPGSQSSSFTTSGQISPTGGDGTAVETKKRGPGKLCQKQPVHRKSIDTFGQRTSQYRGVTRHRWTGRYEAHLWDNSCKKEGQTRKGRQVYLGGYDMEEKAARAYDLAALKYWGPSTHINFPLENYQTELEEMKNMSRQEYVAHLRRKSSGFSRGASVFRGVTRHHQHGRWQARIGRVAGNKDLYLGTFSTQEEAAEAYDIAAIKFRGVNAVTNFDISRYDVEKIMASNTLLAGELARRNKDVEPSNDSSIVPYDSSIVSNNNGGIGIGMEINPDANTANGNANDWKMALYQNPSHHQQQAAAATCVADSLDNHQNKSMAVSGGYRNTSFSMALQDLIGIESLSANTHGIEDDVSKQVTHFSNSSSLVTSLSSSREGSPDKTNVSMPFGKAPPLMASKLIGATNGVGVGSWYPSPQQLRPTAAAAISMAHLPVFATWNDT

**>GLYMA_11G045800**

MKPIENGNTVNINYQNSWLGFSLSPQMINIGVPSHLHQTQPSPAAVETVPPSFYHHTPLHSYGSYYGLEGEHVGLSSSLPIMPLKSNVSLSGIEALSRSQAQATITTSAMHPRLNNMLYNQLLCHGLNNPNNLNHIQENISQQQFSYYSTLRNHDMILEGSKHQLPCIAEDENPGLKSWFSRDFPARHGEESRMIVPLEDNNGGESGSIGSITYGDLHSLNLSVSPTSGSSCVTSSPAITNTVATDTKKRGLQMVDQNQKQIGHRKSIDTFGQRTSQYRGVTRHRWTGRYEAHLWDNSCKKEGQGRKGRQVYLGGYDMEEKAARAYDMAALKYWGPSSHINFPLENYQNELEEMKNMTRQEYVAHLRRKSSGFSRGASMYRGVTRSHRLAFTHRHHQHGRWQARIGRVAGNKDLYLGTFSTQEEAAEAYDIAAIKFRGVNAVTNFDITRYDVERIMESNNLLSSEQAKRKREMDDGTRSEATVNQKPCTTQETVLMQKRCRSQSEWKMVQYPSSQQLDQNPQRIECSRTESLSTALDNMFHHQVEESSNMGTHLSNPSSLVTSLSSSREESPDKTSLPMLFGMPSTVSKLLADVDSLDLSSNLRPSLSIPQMPIFAAWTDA

**>GLYMA_05G108600**

MKSMENDDNADLNNQNNWLGFSLSPQMHNIGVSSHSQPSSAAEVVPTSFYHHTAPLSSYGFYYGLEAENVGLYSALPIMPLKSDGSLYGLETLSRSQAQAMATTSTPKLENFLGGEAMGTPHHYECSATETMPLSLDSVFYIQPSRRDPNNNQTYQNHVQHISTNQQQQQQELQAYYSTLRNHDMILEGSKQSQTSDNNNLHVQNMGGDDAVPVPGLKSWEVRNFQASHAHESKMIVPHVEENAGESGSIGSMAYGDLQSLSLSMSPSSQSSSVTSSHRASPAVVDSVAMDTKKRGPEKVDQKQIVHRKSIDTFGQRTSQYRGVTRHRWTGRYEAHLWDNSCKKEGQSRKGRQVYLGGYDMEEKAARAYDLAALKYWGPSTHINFPLENYQNELEEMKNMTRQEYVAHLRRKSSGFSRGASMYRGVTRSHRPTTTHRHHQHGRWQARIGRVAGNKDLYLGTFSTQEEAAEAYDIAAIKFRGANAVTNFDITRYDVEKIMASSNLLSSELARRNRETDNETQCIDQNHNKPSAYEDTQEAILMHQKSCESENDQWKMVLYQSSQQLEQNPPTIESDRTNQSFAVALDNMFHQEVEESSKARTHVSNPSSLATSLSSSREGSPDRTSLPMLSGMPSTASKLLATNPNNVNSWDPSPHLRPALTLPQMPVFAAWTDA

**>GLYMA_17G158300**

MKSMENDDTVDLNNQNNNWLGFSLSPQMNNNNIGVSTHTQPSSAAAEVVPTSFYHTTPLSSYGFYYGLEAENVGLYSALPIMPLKSDGSLYGMEAVSRSQAQAMATTSTPKLENFLGGEAMGTPHHHYECSATETMPLSLDSVFYNQPSRRDQNNNQTYQNHVQHISTQQQQQQELQAYYSTLRNHDMMLEGSKQSQTSENNNNNLQVQNMGDDAVSVPVAGLKSWGVRNFQASHAHESKMIVPHHVEENGGESGSIGSMAYGDLQSLSLSMSPSSQSSCVTSSHRASSAVIDSAAMDTKKRGSEKVDQKQIVHRKSIDTFGQRTSQYRGVTRHRWTGRYEAHLWDNSCKKEGQSRKGRQVYLGGYDMEEKAARAYDLAALKYWGPSTHINFPLENYQNELEEMKNMTRQEYVAHLRRKSSGFSRGASMYRGVTRSHRPTTTHRHHQHGRWQARIGRVAGNKDLYLGTFSTQEEAAEAYDIAAIKFRGANAVTNFDITRYDVEKIMASSNLLSSELARRNQETTDNGTQYIDQNHNKPSAYEDTQEAAILMHQKSCETQNDQWKMVLYQQSSQQLEQNIPPRIESDRTNQSFSVALENMFHQEVEESSKVRTHVSNPSSLATSLSSSRECSPDRTSLPMLSGVPSTSSKLLATNPNNVNSWDPSPHLRPALTLPQMPVFAAWTDA

**>GLYMA_01G195900**

MKPIENGNTVSINYQNSWLGFSLSPQMNIGVPSHLHQTQPSSAAVEAVPPNFYHHTPLHNYGLYYELEGEHVGMSSSLPIMPLKSNASLSGIEALSRSQAQATTTISAMHSSLNSMLINELLCHGLNNPNNLNHVQEDISQQQFSYYSTLRNQDVILEGSKHQLPCIAEDENPGLKSWFSRDFHARHAEESRMIVPLECNGGESGSIGSITYGDLHSSNLSVSPTSGSSSVTSSPALTNTVATNTKKRWLEMVDQNQKQIVHRKSIDTFGQRTSQYRGVTRHRWTGRYEAHLWDNSCKKEGQGRKGRQVYLGGYDMEEKAARAYDMAALKYWGPSSHINFPLENYQNELEEMKNMTRQEYVAHLRRKSSGFSRGASMYRGVTRSQRLAFTQHHQHGRWQARIGRVAGNKDLYLGTFSTQEEAAEAYDIAAIKFRGVNAVTNFDITRYDVEKIMESNNLLSSEQAKRKREMDDGTRSEATVNQKPSTYDHTQETILMQKRCKNQSEWKMVQFPCPQQLDQNQRIESCRTQPFSTDLDNMFRHQVEERSNMGTHLSNPSSLVTSLSSSREESPDKTSMPMLFGMPSTVSKLLANVDSWDLSSNLRTALSMPQMPIFAAWTDA

**LEUNIG (LUG)**

**>AT4G32551 (AtLUG)**

MSQTNWEADKMLDVYIHDYLVKRDLKATAQAFQAEGKVSSDPVAIDAPGGFLFEWWSVFWDIFIARTNEKHSEVAASYIETQMIKAREQQLQQSQHPQVSQQQQQQQQQQIQMQQLLLQRAQQQQQQQQQQHHHHQQQQQQQQQQQQQQQQQQQQHQNQPPSQQQQQQSTPQHQQQPTPQQQPQRRDGSHLANGSANGLVGNNSEPVMRQNPGSGSSLASKAYEERVKMPTQRESLDEAAMKRFGDNVGQLLDPSHASILKSAAASGQPAGQVLHSTSGGMSPQVQTRNQQLPGSAVDIKSEINPVLTPRTAVPEGSLIGIPGRFAVLSVSFQLVKRVKCSISTSAHKNLLRINDWFPVSGSNQGSNNLTLKGWPLTGFDQLRSGLLQQQKPFMQSQSFHQLNMLTPQHQQQLMLAQQNLNSQSVSEENRRLKMLLNNRSMTLGKDGLGSSVGDVLPNVGSSLQPGGSLLPRGDTDMLLKLKMALLQQQQQNQQQGGGNPPQPQPQPQPLNQLALTNPQPQSSNHSIHQQEKLGGGGSITMDGSISNSFRGNEQVLKNQSGRKRKQPVSSSGPANSSGTANTAGPSPSSAPSTPSTHTPGDVISMPNLPHSGGSSKSMMMFGTEGTGTLTSPSNQLADMDRFVEDGSLDDNVESFLSQEDGDQRDAVTRCMDVSKGFTFTEVNSVRASTTKVTCCHFSSDGKMLASAGHDKKAVLWYTDTMKPKTTLEEHTAMITDIRFSPSQLRLATSSFDKTVRVWDADNKGYSLRTFMGHSSMVTSLDFHPIKDDLICSCDNDNEIRYWSINNGSCTRVYKGGSTQIRFQPRVGKYLAASSANLVNVLDVETQAIRHSLQGHANPINSVCWDPSGDFLASVSEDMVKVWTLGTGSEGECVHELSCNGNKFQSCVFHPAYPSLLVIGCYQSLELWNMSENKTMTLPAHEGLITSLAVSTATGLVASAS

HDKLVKLWK*

**>BnaA03g52120D**

MSQTNWEADKMLDVYIYDYLVKRDLKATAQAFQAEGKVSSDPVAIDAPGGFLFEWWSVFWDIFIARTNEKHSEVAASYIEVPFFVLHLCKAVVTTTTAAANTDATTLVATCPTTTATATAATTSTSTAKPTTFSTAAAASSSAPTARRDGSLHSNGSANGLVGNNGDPVMRQNPGSGSALANNKTYEERVKMATQRESLDEAAMKRFGDNAGQLLDPNHASMLKSSVASGQHAGQVLHGASGGMSPQVQARNQQLPGSAVDIKNEINPVLTPRTAVPEGSLIGIPGSNQGSNLTLKGWPLTGFDQLRSGLLQQQKPFMQSPQSFHQLNMLTPQHQQQLMMAQQNLNSQSVNEENRRLKMLLNNRSMSLGKDGLGSSVGDVLPNVGSSLQPVPGGPLLPRGDTDMLLKLKMALLHQQQQQGGGSIPQPQTLNQQALSNQQSQSSNHNIHQQDKLGGVGSITMDGSMSNSYRGNEQVLKNQSGRKRKQPVSSSGPANSTGTANTTGPSPSSAPSTPSTHTPGDVMPNLPHSGGSSKPMIMFGSDGAGTLTSPSNQLPDMDRFVEDGSLDDNVESFLSHEDGDQRDAVGRCMDVSKGFTFTEVNSVRASTGKVICCHFSSDGKMLASAGHDKKAVIWHTDTMKPKTTLEEHTAMITDVRFSPSLPRLATSSFDKTVRVWDADNKGYSLRNFIGHSSMVTSLDFHPNKDDLICSCDNDGEIRYWSINNGSCTRVYKGGSTQMRFQPRVGKYLAASSSNVVSVLDVETQACRHSLQGHTNPINSVCWDPSGDFLATVSEDMVKVWTLGTGSEGECVHELSCNGNKFQSCVFHPTYPSLLVIGCYQSLELWNMSENKTMALPAHEGLIASLAVSTATGLVASASHDKLVKLWK

**>BnaC01g05930D**

MSQTNWEADKMLDVYIHDYLVKRDLKATAQAFQAEGKISLDPVAIDAPGGFLFEWWSVFWDIFIARTNEKHSEVAASYIETQMMKARDQQMQQASSTTTTANTNATTLQHQQQQHLNQPPSQQQQQQQQQQQQQQAAPQHQQQPTPQQQQQQQQQQQQQPQRRDGSHLANGSANGLVGNNSDPVMRQNSGSASALANNKAYEERIKMPTQRDSLDEAAMKRFGENGGQMLDPNHASMLKSAGASGQPAGQVLHGASSGMSPQVQARNQQLPGSAVDMKSDINTVLTPRTAVPEGSLIGIPGSNQGNNLTLKGWPLTGFDQLRSGLLQPQKPFMQSPQQSFHGLNMLTPQHQQQLMMAQQNLNSQSVNDENRRLKMLLNNRNMSLGKDGLEGGSVGDVLPNNVGSSLQPGGSLLPRGDTDMLLKLKMALLQQQQHQQQGGGNLAQPQALNQHALSNQQTQSSNHNIQQQDKLGGGGSITMDGGMSNSFRGNEQVLKNQTGRKRKQPVSSSGPANSTGTANTTGPSPSSAPSTPSTHTPGDAVSMPNLPHGGGSTKPVIMFGTEGTTGTLTSPSNQLTDMDRFVEDGSLDDNVESFLSHEDGDQRDTVGRCMDVSKGFTFAEVNSVRASTSKVTCCHFSLDGKMLASAGHDKKAVIWHTDTMKPKTTLEEHTAMITDVRFSPNLPRLATSSFDKTVRVWDADNKGYSLRNFIGHSSMITSVDFHPNKDDLICSCDDDGEIRYWSINNGTCSRVYKGGSTQTRFQPRVGKYLAASSANVVSVLDVETQACRHSLQGHTNQINSVCWDASGDFLATVSEDMVKVWTFGTGNEGECVHELSCNGNKFQSCVFHPTYPSLLVVGCYQSLELWNMSQNKTMTLPAHDGLITSLAVSTATGLVASASHDKLVKLWK

**>BnaA08g12150D**

MSQTNWEADKMLDVYIYDYLVKRDLKATAQAFQAEGKVSSDPVAIDAPGGFLFEWWSVFWDIFIARTNEKHSEVAASYIETQMIKAREQQLQQSQHPQISQQQQQQQQQQIQMQQLLLQRAQQQQQQQHNQQQQQQHQNQPPSQQQQQQQQQQQPAPQHQQQSAPQQLPQRRAAPHLANGSANGLVGSNSDPVMRQNSVSGSALANNNKAYEERVKIPTQRDSLDETAMKRFGDNVGQLLDPNHASMLKSAGASGQPAGQVLHGASAGMSPQVQARNQQLSGSAMDIKSEINPVLTPRTPVPEGSLIGIPGSNQGSNLTLKGWPLTGFDQLRSGLLQQQKPFMQSPQSFHQLNMLTPQHQQQLMMAQQNLNSQTVNEENRRLKMLLNNRSMSLGKDGLGGSVGDVLPNVGSSLQPGGTLLPRGDTDMLLKLKMALLQQQHQQQGGGNLPQPQSLNQHALSNQQSQSSNHNINQQDKLGGGGSITMDGSMSNSFRGNEQVLKNQTGRKRKQPVSSSGPANSTGTANTTGPSPGSAPSTPSTHTPGDAISMPNLPRSGSSKAITMFGTDGTATLTSPSNQLADMDRFVEDGSLDDNVESFLSNEDGDQRDAVGRCMDVSKGFTFTEVNSVRASTSKVTCCHFSSDGKMLASAGHDKKAVLWHTDTMKPKTTLEEHTAMITDVRFSPSLPRLATSSFDKTVRVWDADNKGYSLRTFMGHSSMVTSLDFHPNKDDLICSCDTDGEIRYWSINNGSCTRVYKGGSTQLRFQPRVGKYLAASSANVVSVLDVETQACRHSLQGHANQINSVCWDPSGDFLASVSEDMVKVWTLGTGSEGECVHELSCNGNKFQSCVFHPTYPSLLVIGCYQSLELWNMSENKTMTLPAHEGLIASLAVSTATGLVASASHDKLVKLWK

**>Csa_2G379340**

MSQTNWEADKMLDVYIHDYLVKRDLKATAQAFQAEGKVSSDPVAIDAPGGFLFEWWSVFWDIFIARTNEKHSDVAASYIETQLIKAREQQQHQQQQQQQTQPQQQQPQHMQMLLMQRHAQQQQQQQQQQQHQQQQQPQQQQQQSQQQQQQRRDGAQLLNGSSNGFVGNDPLMRQNPGSVNALATKMYEDRLKLPLQRDSLDDGAMKQRYGDNVGQLLDPNHASILKSAAATSQSSGQVLHGSTGGMSPQVQPRSQQLPGSTPDIKTEINPVLNPRAAGPEGSLMGIPGSNHGGNNLTLKGWPLTGLDQLRSGILQQQKPFIQAPQSFPQLQMLTPQHQQQLMLAQQNLTSPSVNDDGRRLRMLLNTRMAKDGLSNSVGDVVPNVGSPLQAGSPLLPRGDNTDMILKIKMAQLQQQQQQQQSSSQQQQQQQLQQHALSNQQSQSSNHNMHQQEKIGGAGSVTMDGSMSNSFRGNDQVSKNQTGRKRKQPVSSSGPANSSGTANTAGPSPSSAPSTPSTHTPGDAISMPALPHSGSSSKPLTMMFNSDGTGTFTSPSNQLWDDKELELQADMDRYVEDGSLDDNVDSFLSHDDGDPRDPVGRCMDGSKGFTFTEVNSVRASTSKVSSCHFSSDGKLLVSGGHDKKAVLWYTENLKPKTSLEEHAAIVTDVRFSPSMPRLATSSFDRTVRVWDADNHCYSLRTFTGHSASVMSLDFHPKKDDFICSCDGDGEIRYWNITNGSCAAVFKGGTGPMRFQPRLGRYFSAVVDNIVTIFDVETQARVHSLRGHTKTVQSLCWDPSGEFLASVSEDSVRVWTLASGNEGESIHELSCNGNKFHSCVFHPTYSTLLVIGCYESLELWNTTENKTMTLSAHEGLVSSLAVSAASGLVASASHDRFIKLWK

**>GLYMA_17G205700**

MSQTNWEADKMLDVYIHDYLVKRDLKASAQAFQAEGKVSSDPVAIDAPGGFLFEWWSVFWDIFIARTNEKHSEVAASYIETQLIKAREQQQQQQQQQQQQQQGQQQPQPQKLQHQQQQQQQQHMQMQQILLQRAQQQQQQQQQQQQQQQQPQQQQQQQQPQQQHQQQQQPQQQGRDRAHLLNGGTNGLVGNPSTANALATKMYEERLKLPLQRDSLEDAAMKQRFGDQILDPNHASILKSSAATGQPSGQVLHGAAGAMSPQVQARSQQLPGSTPDIKSEINPVLNPRAAGPEGSLIAMPGSNQGSNNLTLKGWPLTGLEQLRSGLLQQQKPPFIQSPQQFHQLPMLTPQHQQQLMLAQQNLASPSASDDSRRIRMLLNNRNMGVTKDGLSNPVGDIVSNVGSPLQAGGPAFPRSDTDMLMKLKLAQLQHQQQQNANPPQQQLQQHTLSNQQSQTSNHSMHQQDKMGGGGGSSVNVDGSMSNSFRGNDQVSKNQTGRKRKQPASSGPANSSGTANTAGPSPSSAPSTPSTHTPGDVMSMPVLPHSGSSSKPLMMFSADGAGTLTSPSNQLWDDKDLELQADVDRFVEDGSLDDNVESFLSPDDTDLRDTVGRCMDVSKGFTFSEINSVRASTTKVGCCHFSSDGKLLASGGHDKKAVLWFTDSLKQKATLEEHASLITDVRFSPSMPRLATSSHDKTVRVWDVENPGYSLRTFTGHSSPVMSLDFHPNKDDLICSCDADGEIRYWSINNGNCARVSKGGAVQMRFQPRLGRYLAAAAENVVSILDVETQASRYSLKGHTKSIRSVCWDPSGEFLASVSEDSVRVWTLGSGSEGECVHELSCNGNKFHSCVFHPTYSSLLVVGCYQSLELWNMTENKTMTLSAHEGLIAALAVSTVNGLVASASHDKFVKLWK

**>GLYMA_06G072200**

MSQTNWEADKMLDVYIHDYLVKRDLKASAQAFQAEGKVSSDPVAIDAPGGFLFEWWSVFWDIFIARTNEKHSEVAASYIETQLIKAREQQQQQNQQQPQPQQSQHQQQHMQMQQLLLQRAQQQQQQQQQQQQQPQSQQQQSQPQQQQQSRDRPHLLNGSANGLVGNPGTANALATKMYEERLKLPLQRDSLDDAATKQRFGENMGQLLDPNHAPILKSAAAPGQPSGQVLHGAAGGMSPQVQARTQQLPGSTLDIKGEISPVLNPRAVGPEGSLMGMPGSNPGSNNLTLKGWPLTGLEQLRSGLLQQQKPFMQAPQPFHQLQMLTPQHQQQLMLAQQNLASPSASEESRRLRMLLNNRNI

GLNKDGLSNPVGDVVSNVGSPLQGGGPPFPRGDTDMLVKLKLAQLQQQQQQQSSTNAQQQQLQQHTLSNQQSQTSNHSMHQQDKVGGGGGGSVTVDGSMSNSFRGNDQVSKNQIGRKRKQPGSSSGPANSSGTANTTGPSPSSAPSTPSTHTPGDVISMPALPHSGSSSKPLMMFSTDGTGTLTSPSNQLWDDKDLELQADVDRFVEDGSLDENVESFLSHDDTDPRDTVGRCMDVSKGFTFSDVNSVRASTSKVSCCHFSSDGKLLASGGHDKKVVLWYTDSLKQKATLEEHSSLITDVRFSPSMPRLATSSFDKTVRVWDVDNPGYSLRTFTGHSTSVMSLDFHPNKDDLICSCDGDGEIRYWSINNGSCARVSKGGTTQMRFQPRLGRYLAAAAENIVSIFDVETQVCRYSLKGHTKPVVCVCWDPSGELLASVSEDSVRVWTLGSGSDGECVHELSCNGNKFHKSVFHPTYPSLLVIGCYQSLELWNMSENKTMTLSAHDGLITSLAVSTVNGLVASASHDKFLKLWK

**>GLYMA_14G128300**

MSQTNWEADKMLDVYIHDYLVKRDLKASAQAFQAEGKVSSDPVAIDAPGGFLFEWWSVFWDIFIARTNEKHSEVAASYIETQLIKAREQQQQQQQPQPQQSQHQQQQQQQQQQQQQQHMQMQQILLQRAQQQQQQQQQQQQQQPQQQQLQQPQLQHQQQPPQQQQGRDRAHLLNGGTNGLVGNPGTANALATKMYEERLKLPLQRDSLEDAAMKQRYGDQLLDPNHASILKSSAATGQPSGQVLHGAAGAMSSQVQARSQQLPGSTPDIKSEINPVLNPRAAAPEGSLIAMPGSNQGSNNLTLKGWPLTGLEQLRSGLLQQQKPPFIQSPQPFHQLPMLTPQHQQQLMLAQQNLASPSASDDNRRLRMLLNNRNIGVTKDGLSNPVGDIVSNLGSPLQAGGPAFPRSDTDMLMKLKLAQLQHQQQNANPQQQQLQQHTLSNQQSQTSNHSMHQQDKMGGGGGSVNVDGSMSNSFRGNDQVSKNQTGRKRKQPASSGPANSSGTANTAGPSPSSAPSTPSTHTPGDVMSMPALPHSGSSSKPLMMFSADGSGTLTSPSNQLWDDKDLELQADVDRFVEDGSLDDNVESFLSHDDTDPRDTVGRCMDVSKGFTFSEINSVRASTNKVVCCHFSSDGKLLASGGHDKKAVLWFTDSLKQKATLEEHAYLITDVRFSPSMPRLATSSYDKTVRVWDVENPGYSLRTFTGHSSSVMSLDFHPNKDDLICSCDVDGEIRYWSINNGSCARVSKGGTAQMRFQPRLGRYLAAAAENVVSILDVETQACRYSLKGHTKSIHSVCWDPSGEFLASVSEDSVRVWTLGSGSEGECVHELSCNGNKFHSCVFHPTYSSLLVVGCYQSLELWNMTENKTMTLSAHEGLIAALAVSTVNGLVASASHDKFVKLWK

**REVOLUTA (REV)**

**>AT5G60690 (AtREV)**

MEMAVANHRERSSDSMNRHLDSSGKYVRYTAEQVEALERVYAECPKPSSLRRQQLIRECSILANIEPKQIKVWFQNRRCRDKQRKEASRLQSVNRKLSAMNKLLMEENDRLQKQVSQLVCENGYMKQQLTTVVNDPSCESVVTTPQHSLRDANSPAGLLSIAEETLAEFLSKATGTAVDWVQMPGMKPGPDSVGIFAISQRCNGVAARACGLVSLEPMKIAEILKDRPSWFRDCRSLEVFTMFPAGNGGTIELVYMQTYAPTTLAPARDFWTLRYTTSLDNGSFVVCERSLSGSGAGPNAASASQFVRAEMLSSGYLIRPCDGGGSIIHIVDHLNLEAWSVPDVLRPLYESSKVVAQKMTISALRYIRQLAQESNGEVVYGLGRQPAVLRTFSQRLSRGFNDAVNGFGDDGWSTMHCDGAEDIIVAINSTKHLNNISNSLSFLGGVLCAKASMLLQNVPPAVLIRFLREHRSEWADFNVDAYSAATLKAGSFAYPGMRPTRFTGSQIIMPLGHTIEHEEMLEVVRLEGHSLAQEDAFMSRDVHLLQICTGIDENAVGACSELIFAPINEMFPDDAPLVPSGFRVIPVDAKTGDVQDLLTANHRTLDLTSSLEVGPSPENASGNSFSSSSSRCILTIAFQFPFENNLQENVAGMACQYVRSVISSVQRVAMAISPSGISPSLGSKLSPGSPEAVTLAQWISQSYSHHLGSELLTIDSLGSDDSVLKLLWDHQDAILCCSLKPQPVFMFANQAGLDMLETTLVALQDITLEKIFDESGRKAICSDFAKLMQQGFACLPSGICVSTMGRHVSYEQAVAWKVFAASEENNNNLHCLAFSFVNWSFV*

**>BnaA06g18550D**

MAVAVGNRHESGENINRHLDSSGKYVRYTGEQVEALERVYSECPKPTSLRRQQLIRECPFLANIEPKQIKVWFQNRRCRDKQRKEASRLQSVNQKLSAMNKLLMEENDRLQKQVSHLVSENGYMQQQLTLTTLGTDASCDSVDPTPPLHPLRDANSPAGLMAIAEETLAEFLSKATGTAVDWVQMPGMKPGPDSVGIFAISQKCYGVAARACGLVSLEPMKIVEILKDRPSWFRDCRSIEVFTMFPAGNGGTIELIYMQTYAPTTLAPARDFWTLRYTTSLEKGSIVVCERSLSGSGAGPNATSAAQFVRAEMLPSGYLIRPCDGGGSIIHIVDHINFEGWSVPDVLRLLYESSKVVAQRMTIAALRYVRQVAHETNGEVVYGLGRQPAVLRTFSQRLSRGFSDAVNGFNDDGWSIMHCNGAEDITVAVNSTKHLNSFSDPLSFLGGVLCAKASMLLQNVCPAVLVRFLREHRSEWADFNVDAYSAATLKAGAFAYSGMRPTTFTGSQIIMPLGNTIEKEEMLEVVRLEGHSLVPEDSFLSRDVHLLQICTGIDEDVVGACSELVFAPVNEMFPDDAPLVPSGFRVIPVDSKTGDAQDLLTANHRTLDLTSSQDVGSTPETGSSPSSRCILTIAFQFPFENNLQENVANMACQYVRSVISSVQRVAVALSPSGLIPIPGSKLSPGSPEAVSLAIWICQSYKQHFGSDLLRTDSLGGDALLRQLWDHQDAILCCSLKPQPVFMFANQAGLDMLETTLVALQDIALEKIFDESGRKALCPDFAKLMQQGFACLPSGMCVSTMGRHVSYEQAVSWKVFSDCEDNNNNRIHCLAFLFANWSFL

**>Csa_6G141360**

MAMAIAHHRESSTGSITRHLDSSGKYVRYTSEQVEALERVYAECPKPSSLRRQQLVRDCPILSNIEPKQIKVWFQNRRCREKQRKEASRLQTVNRKLNAMNKLLMEENDRLQKQVSQLVCENGFMRQQLHTVPAAATADASCDSVVTTPQPSRRDANNPAGLLSIAEETLAEFLSKATGTAVDWVQMPGMKPGPDSVGIFAISQSCGGVAARACGLVSLEPSKIAEILKDRPSWFRDCRSLEVFTMFPAGNGGTIELVYTQVYAPTTLAPARDFWTLRYTITLENGSLVVCERSLSGSGAGPSEAAAAQFVRAEMLPSGYLIRPCEGGGSIIHIVDHLNLEAWHVPEVLRPLYESSKVVAQKMTIAALRYVRQIAQETSGEVVYGLGRQPAVLRTFSQRLSRGFNDAVNGFNDNGWSLINCEGAEDVVLTVNSTKNFGTTSNPANSLTYPGGVLCAKASMLLQNVPPAVLVRFLREHRSEWADFNIDAYSAATLKANSYTYPGMRPTRFTGSQIIMPLGHTIEHEELLEVIRLEGHPMVQEDAFVSRDIHLLQICSGIDENAVGACSELIFAPIDEMFPDDAPLLPSGFRIIPLDSRTSDAKGSQRTLDLTSSLEVGSGTSNTAGDASSSQSARSVLTIAFQFPFESSMQDNVANMAHQYVRSVISSVQRVAMAISPSGGGPALGPKLSPGSPEALTLAHWICKSYSLQLGTELIKSDSLEGDSLLKNLWNHQDAILCCSLKQSLPVFLFANQAGLDMLETTLVALQDITLDKIFDESGRKALCADFPKLMQQGFAYLPGGICASTMGRHVSYEQAVAWKVLEADETTVHCLAFSFINWSFV

**>GLYMA_12G075800**

MAMAVAQHRESSSSGSIDKHLDSGKYVRYTAEQVEALERVYAECPKPSSLRRQQLIRECPILSNIEPKQIKVWFQNRRCREKQRKEASRLQTVNRKLTAMNKLLMEENDRLQKQVSQLVCENGFMRQQLHTPSAATTDASCDSVVTTPQHTMRDANNPAGLLSIAEETLTEFLSKATGTAVDWVQMPGMKPGPDSVGIFAISQSCSGVAARACGLVSLEPTKIAEILKDRPSWFRDCRSLEVFTMFPAGNGGTIELVYTQTYAPTTLAPARDFWTLRYTTSLENGSLVVCERSLSGSGTGPNPAAAAQFVRAETLPSGYLIRPCEGGGSIIHIVDHLNLEAWSVPEVLRPLYESSKVVAQKMTIAALRYIRQIAQETSGEVVYGLGRQPAVLRTFSQRLSRGFNDAVNGFNDDGWTVLNCDGAEDVFIAVNSTKNLSGTSNPASSLTFLGGILCAKASMLLQNVPPAVLVRFLREHRSEWADFSVDAYSAASLKAGTYAYPGMRPTRFTGSQIIMPLGHTIEHEEMLEVIRLEGHSLAQEDAFVSRDIHLLQICSGIDENAVGACSELVFAPIDEMFPDDAPLIPSGFRIIPLDSKPGDKKEVATNRTLDLTSGFEVGPATTAGTDASSSQNTRSVLTIAFQFPFDSSLQDNVAVMARQYVRSVISSVQRVAMAISPSGISPSVGAKLSPGSPEAVTLAHWICQSYSYYIGSDLLRSDSLVGDMMLKQLWHHQDAILCCSLKPLPVFIFANQAGLDMLETTLVALQDITLDKIFDEAGRKALCTDFAKLMEQGFAYLPAGICMSTMGRHVSYDQAIAWKVLTGEDNTVHCLAFSFINWSV

**>GLYMA_11G145800**

MAMVVAQHRESSSSGSIDKHLDSGKYVRYTAEQVEALERVYAECPKPSSLRRQQLIRECPILSNIEPKQIKVWFQNRRCREKQRKEASRLQTVNRKLTAMNKLLMEENDRLQKQVSQLVCENGFMRQQLHTPSATTTDASCDSVVTTPQHTLRDASNPAGLLSIAEETLTEFLSKATGTAVDWVQMPGMKPGPDSVGIFAISQSCSGVAARACGLVSLEPTKIAEILKDRPSWFRDCRSLEVFTMFPAGNGGTIELVYTQTYAPTTLAPARDFWTLRYTTSLENGSLVVCERSLSGSGTGPNPAAAAQFVRAETLPSGYLIRPCEGGGSIIHIVDHLNLEAWSVPEVLRPLYESSKVVAQKMTIAALRYIRQIAQETSGEVVYGLGRQPAVLRTFSQRLSRGFNDAVNGFNDDGWTVLNCDGAEDVIIAVNSTKNLSGTSNPASSLTFLGGILCAKASMLLQNVPPAVLVRFLREHRSEWADFNVDAYSAASLKAGTYAYPGMRPTRFTGSQIIMPLGHTIEHEEMLEVIRLEGHSLAQEDAFVSRDIHLLQICSGIDENAVGACSELVFAPIDEMFPDDAPLVPSGFRIIPLDSKPGDKKDAVATNRTLDLTSGFEVGPATTAGADASSSQNTRSVLTIAFQFPFDSSLQDNVAVMARQYVRSVISSVQRVAMAISPSGINPSIGAKLSPGSPEAVTLAHWICQSYSYYLGSDLLRSDSLVGDMMLKQLWHHQDAILCCSLKSLPVFIFANQAGLDMLETTLVALQDITLDKIFDEAGRKALCTDFAKLMEQGFAYLPAGICMSTMGRHVSYDQAIAWKVLTGEDNTVHCLAFSFINWSFV

**SEUSS (SEU)**

**>AT1G43850 (AtSEU)**

MVPSEPPNPVGGGENVPPSILGGQGGAPLPSQPAFPSLVSPRTQFGNNMSMSMLGNAPNISSLLNNQSFVNGIPGSMISMDTSGAESDPMSNVGFSGLSSFNASSMVSPRSSGQVQGQQFSNVSANQLLAEQQRNKKMETQSFQHGQQQSMQQQFSTVRGGGLAGVGPVKMEPGQVSNDQQHGQVQQQQQKMLRNLGSVKLEPQQIQAMRNLAQVKMEPQHSEQSLFLQQQQRQQQQQQQQQFLQMPGQSPQAQMNIFQQQRLMQLQQQQLLKSMPQQRPQLPQQFQQQNLPLRPPLKPVYEPGMGAQRLTQYMYRQQHRPEDNNIEFWRKFVAEYFAPNAKKRWCVSMYGSGRQTTGVFPQDVWHCEICNRKPGRGFEATAEVLPRLFKIKYESGTLEELLYVDMPRESQNSSGQIVLEYAKATQESVFEHLRVVRDGQLRIVFSPDLKIFSWEFCARRHEELIPRRLLIPQVSQLGSAAQKYQQAAQNATTDSALPELQNNCNMFVASARQLAKALEVPLVNDLGYTKRYVRCLQISEVVNSMKDLIDYSRETRTGPIESLAKFPRRTGPSSALPGPSPQQASDQLRQQQQQQQQQQQQQQQQQQQQQQQQTVSQNTNSDQSSRQVALMQGNPSNGVNYAFNAASASTSTSSIAGLIHQNSMKGRHQNAAYNPPNSPYGGNSVQMQSPSSSGTMVPSSSQQQHNLPTFQSPTSSSNNNNPSQNGIPSVNHMGSTNSPAMQQAGEVDGNESSSVQKILNEILMNNQAHNNSSGGSMVGHGSFGNDGKGQANVNSSGVLLMNGQVNNNNNTNIGGAGGFGGGIGQSMAANGINNINGNNSLMNGRVGMMVRDPNGQQDLGNQLLGAVNGFNNFDWNA*

**>BnaCnng63720D**

MFGTESLAKFPRRTGPSSALPGPSAQQPNEQPRQQQHQSVAQIANKDQSCGQSSLNYAFNAASASTSTGSIHQNSMKQRNQNAAYKTPSSPYGGISIQMQSPSNSGTMAPSSQQQHNLPSFQSPTSSSNNNYPSQNGITSINNHMGSTNLPAIQQAAADEANESSSVQKILNEILMNNNQTHNTSGGGGHESFGNDGKGGRNVSSSGVLMMNNGQVNTSIGGFGMTNINGNNGLMNGRAGMMVRDPNVQQDVGNQRLGGAVNAFNNFQCDWNV

**>Csa_6G343700**

MVTSGPPTPMGGGAQSVSPSLLRSNSGLLGVQGGMLPSQAAFSSLVSPRNQFNNMNMLGNMSNVSSLLNQSFGNGAPNSGLPCPGNNHPGAEPDPLSAVGNGMSFNNPSSSFVASNMANPVSSVQGQNPQFSNLSSNQLLSDQQQSQQLEPQNFQHSQQSMEQFSALQSNQQPQFQAIRGLPGVGPVKLEPQVTSNDQHGQQQQQQQHLQTLRNLGSVKLESQRLQSMRGLAPVKMEPQQSDQSLFQQQQQQQQQQQQQQQQQHQHPHPHQQQQQSQQFLHMSRQSSQVAAAQINLMHQQRILQLQQHQQLLKSMPPQRPQLQQHYQQQNLSLRSPVKPGYEPGMCARRLTHYMYHQQHRPEDNNIDFWRKFVNEYFAPHAKKKWCVSMYGSGRQTTGVFPQDVWHCEICNRKPGRGFEATAEVLPRLFKIKYESGTMEELLYLDMPREYHNASGQIVLDYAKAIQESVFEQLRVVRDGQLRIVFSPDLKICSWEFCARRHEELIPRRLLIPQVSHLGAAAQKFQSAIQNTSSNLSTPELQNNCNMFVASARQLAKALEVPLVNDLGYTKRYVRCLQISEVVNSMKDLIDYSKETGIGPMDSLAKFPRRTSSSSGVTNQAPISDEQQQQQSSIAQRSNNNQSSVQASAVQQLTASNGVSSVNNTANQPSTSNSASTIAGLLHQNSMNSRQQNSMPNASNSYGGSSVQIPSPGSSSTVPPTQPNPSTFQPPTPSSSNSLSQPSHAVAKNPNQMSAANSPANISMQQQPALSGDADPSETQSSVQKILQEMMMNNQMNGPNSLVGVGSVVNDMKNMNGVLPTSSTGLNNGNCIGGNGAANGGSGMGGGGYGSMGSGLGQPVMVNGMRTAMGNNTIMNRRIGMASLALEQSMNGQPQDMGNQLLGGLGAVNGYSNLQFDWKPSP

**>GLYMA_04G241900**

MVPPGPPTPIGGAQSVSPSLLRSNSGMLGAQGGPMPPQSSFPSLVSPRTQFNNMNILGNMSNVTSILNQSFPNGVPNPGLSGPGNSQRGAIDTGAEKDPVSSVGNGMNFNNSSSTFVQSSIVNAASSGQGQGQQFSNPSSNQLLQDQQHSQQLEPQNFQHGQQSMQQFSAPLNTQQPPQPQQHFQSIRGGMGGMGPVKLEQVSNDQLGQQQQQQLQSLRNLASVKLEPQQMQTMRTLGPVKMEPQHSDQPLFMQQQQQQQQQQQFLHMSNQSSQAAAAQINLLRHHRLLQLQQQHQQQQLLKAMPQQRSQLPQQFQQQNMPMRSPVKPAYEPGMCARRLTHYMYQQQHRPEDNNIDFWRKFVAEYFAPNAKKKWCVSMYGSGRQTTGVFPQDVWHCEICNRKPGRGFEATVEVLPRLFKIKYESGTLEELLYVDMPREYHNSSGQIVLDYAKAIQESVFEQLRVVRDGQLRIVFSPDLKICSWEFCARRHEELIPRRLLIPQVSQLGTVAQKYQSFTQNATPNVSVPELQNNCNMFVASARQLAKALEVPLVNDLGYTKRYVRCLQISEVVNSMKDLIDYSRETGTGPMESLAKFPRRTSGSSGPRGQAQQHEEQLQQQQQQQMVAHNSNGDQNSVQAAAMQIASSNGMVSVNNTVNPASTLTSTSTIVGLLHQNSMNSRQPNSMNNASSPYGGSSVQIPSPGSSSTVPQAQPNSSPFQSPTPSSSNNPPQTSHPALTSANHMSTTNSPANISMQQQQPSISGEPDPSDAQSSVQKIIHEMMMSSQINGNGGMVGVGSLGNDVKNVNGILPVSANTGLNGGNGLVGNGTMNSNSGVGVGNYGTMGLGQSAMPNGIRSAMVNNSIMNGRGGMASLARDQAMNHQQDMSNQLLSGLGAVGGFSNLQFDWKPSP

**>GLYMA_06G121500**

MRIQKPQVESSSLFVPSQFVDQFPDSVTVQFRNFQLSVSHGDCLKTVISKKMVPPGPPTPIGGAQSVSPSLLRSNSGMLGAQGGPMPPQSSFPSLVSPRTQFNNMNILGNMSNVTSILNQSFPNGVPNPGLSGPGSSQRGAIDTGAETDPLSSVGNGMSFNNSSSTFVQSSIVNAASSGQGQGQQFSNPSSNQLLPDQQHSQQLEPQNFQHGQQSMQQFSAPLNTQQPPQPQPHFQSIRGGIGGMGPVKLEQVSNDQLGQQQQQQLQSLRNLASVKLEPQQMQTMRTLGPVKMEPQHSDQPLFLQQQQQQQQQQFLHMSSQSSQAAAAQINLLRHHRLLQLQQQHQQQQLLKAMPQQRSQLPQQFQQQNMSMRSPAKPAYEPGMCARRLTHYMYQQQHRPEDNNIEFWRKFVAEYFAPNAKKKWCVSMYGSGRQTTGVFPQDVWHCEICNRKPGRGFEATVEVLPRLFKIKYESGTLEELLYVDMPREYHNSSGQIVLDYAKAIQESVFEQLRVVRDGQLRIVFSPDLKICSWEFCARRHEELIPRRLLIPQVSQLGAVAQKYQSFTQNATPNVSVPELQNNCNMFVASARQLVKALEVPLVNDLGYTKRYVRCLQISEVVNSMKDLIDYSRETGTGPMESLAKFPRRTSGSAGPRGQAQQHEEQLQQQQQQQMVAHNSNGDQNSVRAAAMQIASSNGMVSVNNSVNPASTSTTTSTIVGLLHQNSMNSRQQNSMNNASSPYGGSSVQIPSPGSSSTVPQGQPNSSPFQSPTPSSSNNPPQTSHPALTSANHTSTTNSPANISMQQQQSSISGEPDPSDAQSSVQKIIHEMMMSSQINGNGGMVGVGSLGNDVKNVSGILPVSANTGLNGGNGLVGNGPMNSNSGVGVGNYGTMGLGQSAMPNGIRTAMVNNSIMNGRGGMASLARDQAMNHQQDLSNQLLSGLGAVGGFNNLQFDWKPSP

**>GLYMA_13G037600**

MVPPGPPTPIGGAQSVPLSLLRSNSGMLAGQGGGAVPSQTSFPSLVGQRNQFNNMNMLGNMSNVTSLLNQSFPNGIPNSGLGGPGSSQRSGGIDAGAEADPLSGVGNGMNFGNQLQSNLMNPGSSGQGQGPQFSNASGSQMLQDQQHSQQLPQNFQQHSQPSMQQFSGPLNAQQQQQQQQHFQSIRGGMGGVGQVKLEPQVNIDQFGQQQQLPSRNLAQVKLEPQQLQTLRNMAPVKMEPQHNDQQFLHQQQQQQQQQQQQQQQQQLLHMSRQSSQAAAAQMNHLLQQQRLLQYQQHQQQQQQLLKAMPQQRSQLPQQFQQQNMPMRSPVKPAYEPGMCARRLTHYMYQQQHRPDDNNIEFWRKFVSEYFAPNAKKKWCVSMYGNGRQTTGVFPQDVWHCEICNRKPGRGFEATAEVLPRLFKIKYESGTLEELLYVDMPREYHNSSGQIVLDYAKAIQESVFEQLRVVRDGQLRIVFSPDLKICSWEFCARRHEELIPRRLLIPQVSQLGAVAQKYQAITQNATPNLSVPELQNNCNMVVASARQLAKALEVPLVNDLGYTKRYVRCLQISEVVNSMKDLIDYSRETGTGPMDSLAKFPRRTSGSSGLHSQGQQSEDQLQQQSQPQLPPQHMVPHSSNGDQNSVQTAAMQIASSNGVTSVNNSVNAASASTSTSTIVGLLHQNSMNSRQNSMNSRQNSMNNASSPYGGSSVQIASPGSSGNMPQAQPNASPFQSPTPSSSNIPQTSHPALTSANHMGTANSPANISLQQQQQTSLPAEADPSDAQSSVQKIIHEMMMSSQMNGPGGMAGAGSLGNDMKNVNGILPGSNNTGLNSGSVSGLVGNVAVNSNSGVGVGGYGTIGLGPAGMTNGMRPVMGHNSIMNGRGGMASLARDQVMNHQQDLSSQLLSGLGGVNGFSNLQFDWKPSP

**>GLYMA_14G119800**

MVPPGPPTPIGGAQPVPPSLLRSNSGMLGGQGGPVPSQTSFPSLVAQRNQFNNMNMLGNMSNVTSLLNQSFPNGIPNSGHGGPGNSQRSGGIDARAEADPLSGVGSGMNFGNQLQSNLMNPGSSGQGQGQQFSNASGSQMLPDQQHSQQLEPQNFQQHSQPSMQQFSAPLNAQQQQQQHFQSIRGGMGGVGQVKLESQVNNDQFGHQQQLPSRNLAQVKLEPQQLQTLRNMAPVKLEPQHNDQQFLHQQQQQQQQHQQQQQQQLLHMSRQSSQAAAAQMNHLLQQQRLLQYQQHQQQQQQLLKTMPQQRSPLSQQFQQQNMPMRSPVKPAYEPGMCARRLTHYMYQQQHRPEDNNIEFWRKFVAEYFAPNAKKKWCVSMYGSGRQTTGVFPQDVWHCEICNCKPGRGFEATAEVLPRLFKIKYESGTLEELLYVDMPREYHNSSGQIVLDYAKAIQESVFEQLRVVRDGQLRIVFSPDLKICSWEFCARRHEELIPRRLLIPQVSQLGVVAQKYQAFTQNATPNLSVPELQNNCNLFVASARQLAKALEVPLVNDLGYTKRYVRCLQISEVVNSMKDLIDYSRETRTGPMDSLAKFPRRTSGSSGLHSQAQQSEDQLQQQSQPPQHMVPHTSNGDQNSVQTAAMQIASSNGVTSVNNSVNAASASASNTTSTIVGLLHQNSMNSRQNSMNNASSPYGGSSVQIPSPGSSGNVPQAQPNQSPFQSPTPSSSNNPQTSHPAITSANHMGTANSPANITLQQQQTSLPAEADPSDAQSSVQKIIHEMMISSQMNGPGGMAGTGLLGNDMKNVNGILPGSNSTGLNSGSGLAGNGAVNSSNSGVGVGGYGTMGLGPSGMTNGMRPVMGHNSIMNGRGGMASLARDQVMNHQQDLSSQLLSGLGGVNGFSNLQFDWKPSP

**AINTEGUMENTA‐LIKE 6 (AIL6)**

**>AT5G10510 (AtAIL6)**

MMAPMTNWLTFSLSPMEMLRSSDQSQFVSYDASSAASSSPYLLDNFYGWSNQKPQEFFKEEAQLAAAASMADSTILTTFVDPQSHHSQNHIPKLEDFLGDSSSIVRYSDNSQTDTQDSSLTQIYDPRHHHNQTGFYSDHHDFKTMAGFQSAFSTNSGSEVDDSASIGRTHLAGDYLGHVVESSGPELGFHGGSTGALSLGVNVNNNTNHRNDNDNHYRGNNNGERINNNNNNDNEKTDSEKEKAVVAVETSDCSNKKIADTFGQRTSIYRGVTRHRWTGRYEAHLWDNSCRREGQARKGRQVFYSFFGMCYLIWGCILALLKINSGYDKEDKAARAYDLAALKYWNATATTNFPITNYSKEVEEMKHMTKQEFIASLRRKSSGFSRGASIYRGVTRHHQQGRWQARIGRVAGNKDLYLGTFATEEEAAEAYDIAAIKFRGINAVTNFEMNRYDVEAIMKSALPIGGAAKRLKLSLEAAASSEQKPILGHHQLHHFQQQQQQQQLQLQSSPNHSSINFALCPNSAVQSQQIIPCGIPFEAAALYHHHQQQQQHQQQQQQQNFFQHFPANAASDSTGSNNNSNVQGTMGLMAPNPAEFFLWPNQSY*

**>BnaC02g00280D**

MAPMTNWLTFSLSPMDMLRSSDQSQFVSYDASSAASSSPYLLDNFYGWTNQKPQEFFKDEAQIAASMADSTILATFVDPQTHSHNHIPKLEDFLGEVRYSDNSQTETQDSSSLTHIYDPRHHQNQNQTGFYTDHNHEFKTMAGFQTAFSTNSGSEVEDSASIGRTHLAGEYLGHVVESSGGPELGFHGGANNGGALSLGVNVNNSNHRTSDDHTQITEHHYRGNNNGERTNNEKTVSEKEKPVVAVETSDCSNKKIADTFGQRTSIYRGVTRHRWTGRYEAHLWDNSCRREGQARKGRQVYLGGYDKEDKAARAYDLAALKYWNATATTNFPITNYSKELEEMKHMTKQEFIASLRRKSSGFSRGASIYRGVTRHHQQGRWQARIGRVAGNKDLYLGTFATEEEAAEAYDIAAIKFRGINAVTNFEMNRYDVEAIMKSALPIGGAAKQQKPILGHQHQLHHFQQQQQQQIQSSPNHSSINFAQSQMIPCGIPFEAAALYHHQQQQQQQQQQNFFQHFPANVRATDSTGSNNNSNVQGSMGLMVPNQAEFFLWPNQSY

**>BnaA10g21750D**

MAPMTNWLTFSLSPMEMLRSSDDQSQFVSYDASSAASSSPYLLDNFYGWTNQKPQELFFKEAAAASMADSTILTTFVDSQTHSQSHIPKLEDFLGGDVRYSDNSQSETQDSSSLSQIYDPRHHQNQNQNQTGFYSDHNPDFNKTMAGFQTAFSTNSGSEVDDSASIARTHLAGEYLGHVVESSGPELGFHGGASTGGALSLGVNINNANHRTSNDTNQITEHHYNKPVVTVETSDCSNKKVADTFGQRTSIYRGVTRHRWTGRYEAHLWDNSCRREGQARKGRQGIHFFFFFNLISILLLQTFLNILIYLGGYDKEDKAARAYDLAALKYWNAAATTNFPIANYSKELEEMKHMTKLEFIASLRRKSSGFSRGASIYRGVTRHHQQGRWQARIGRVAGNKDLYLGTFATEEEAAEAYDIAAIKFRGINAVTNFEMNRYDVEAIMKSALPIGGAAKRLKLSLESAASAEQKPNSSTNNSSINFALCPNSDVQSQMIPCGIPFDAAALYHHQQQQQQQNFFQHFHAASDSAASNNNNSNVQGSMGLMAPNAAEFFLWPNQSY

**>BnaC09g46030D**

MAGFQTAFSTNSGSEVDDSASIARTHLAGEYLGHVVESSGPELGFHGGASTGGALSLGVNINNNANQRISSDNNQIKRDSEKEKPVVAVETSDCSNKKVADTFGQRTSIYRGVTRHRWTGRYEAHLWDNSCRREGQARKGRQAKLIIFAFFLHGPLAFTKINSGYDKEDKAARAYDLAALKYWNAAATTNFPITNYSKELEEMKHMTKLEFIASLRRKSSGFSRGASIYRGVTRHHQQGRWQARIGRVAGNKDLYLGTFATEEEAAEAYDIAAIKFRGINAVTNFEMNRYDVEAIMKSALPIGGAAKRLKLSLESAASAEQKPNSSHNNSSINFALCPNSDVQSQMIPCGIPFDAAALYHHQQQQNFFQHFHAASDSTASNNNNSNVQGSMGLMAPNAAEFFLWPNQSY

**>BnaA03g02990D**

MSRTHLAGEYLGHVVESSGQELGFIHGGANTGGALSLGVNINNTNNHASNDNNKISEYNYREKPVVAVERSDSSNKKVADTFGQRTSIYRGVTRHRWTGRYEAHLWDNSCRREGQARKGRQDFHSFSFCLYLFKFSHFCFLTIMTVYLGGYDKEDKAARAYDLAALKYWNTAATTNFPITNYSKELEEMKHMTKQEFIASLRRKSSGFSRGASMYRGVTRHHQQGRWQARIGRVAGNKDLYLGTFATEEEAAEAYDIAAIKFRGINAVTNFEMNRYDVEAIMKSALPIGGAAKRLKLSLESEQKPIIGHHQLHHFQQQHQQLQSSPNDTAQSQMIPCGIPFEAASLYHHQQQQQQQNFFQHFPANAASDSTDSNNNSNVQSSMGLMAPNAAAEFFLWPNQSY

**>Csa_3G114470**

MAPATNWLSFSLSPIEMLRSSDSPFLPFDSSSSSPSPHYLLDNFYHGWSNNNAANSHSKSSQLFFNNQEEEAAAAAVKDDQTTIFLHPQTQTHHHHHHHHHQPKLEDFLGDSSPMVRYSDSQTDTQDSSLTHIYDHASAPYFPHDQQDLKTIAAFQAFSANSGSEVDDSASIPTTHIPSAHSIDSSLTNNDFPSFSTGALSLAVAQSSDTAPAPVVVAVDSDSSKKIADTFGQRTSIYRGVTRHRWTGRYEAHLWDNSCRREGQARKGRQVYLGGYDKEEKAARAYDLAALKYWGPTATTNFPVSNYAKELEEMKQVTRQEFIASLRRKSSGFSRGASIYRGVTRHHQQGRWQARIGRVAGNKDLYLGTFATEEEAAEAYDIAAIKFRGLNAVTNFEMSRYDVEAIAKSALPIGGAAKRLKLCLESDQKPIPNHDQATQCSSGSNNINFGTAMQAVPPIPCGIPYDTAAVLYHHNYFHHLQPNAIGSSESTSPGIAVPGTVGPHQAAEFFVWPHQSY

**>GLYMA_01G022500**

MARATNWLSFSLSPMEMLRTSEPQFLQYDAASATSSHHYYLDNLYTNGWGNGSLKFEQNLNHSDVSFVESSSQSVGHVPPPPPKLEDFLGDSSAVMRYSDSQTETQDSSLTHIYDHHHHHHHHHGSTSYFGGDQQDLKAITGFQAFSTNSGSEVDDSASIGKAQASEFGTHSIESSGNEFAAFSGGTTGTLSLAVALSSEKAVVAAESNSSKKIVDTFGQRTSIYRGVTRHRWTGRYEAHLWDNSCRREGQARKGRQVYLGGYDKEEKAARAYDLAALKYWGPTATTNFPVSNYSKEVEEMKHVTKQEFIASLRRKSSGFSRGASIYRGVTRHHQQGRWQARIGRVAGNKDLYLGTFATEEEAAEAYDIAAIKFRGANAVTNFEMNRYDVEAIMKSSLPVGGAAKRLRLSLESEQKAPPVNSSSQQQNPQCGNVSGSINFSAIHQPIASIPCGIPFDSTTAYYPHNLFQHFHPTNAGAAASAVTSANATALTALPASAATEFFIWPHQSY

**>GLYMA_09G199800**

MARASTNWLSFSLSPMDMLRTPEPQFVQYDAASDTSSHHYYLDNLYTNGWGNGSLKFEQNLNHSDVSFVQSSSQSVSHAPPKLEDFLGDSSAVMRYSDSQTETQDSSLTHIYDHHHHHHHGSSAYFGGDHQDLKAITGFQAFSTNSGSEVDDSASIGKAQGSEFGTHSIESSVNEFAAFSGGTNTGGTLSLAVAQSSEKAVAAAAESDRSKKVVDTFGQRTSIYRGVTRHRWTGRYEAHLWDNSCRREGQARKGRQVYLGGYDKEEKAARSYDLAALKYWGPTATTNFPVSNYSKEVEEMKHVTKQEFIASLRRKSSGFSRGASIYRGVTRHHQQGRWQARIGRVAGNKDLYLGTFATEEEAAEAYDIAAIKFRGANAVTNFEMNRYDVEAIMKSSLPVGGAAKRLKLSLESEQKALPVSSSSSSSQQQNPQCGNVSASINFSSIHQPIASIPCGIPFDSTTAYYHHNLFQHFHPTNAGTAASAVTSANANALTALPPTAAAEFFIWPHQSY

**PERIANTHIA (PAN)**

**>AT1G68640 (AtPAN)**

MQSSFKTVPFTPDFYSQSSYFFRGDSCLEEFHQPVNGFHHEEAIDLSPNVTIASANLHYTTFDTVMDCGGGGGGGLRERLEGGEEECLDTGQLVYQKGTRLVGGGVGEVNSSWCDSVSAMADNSQHTDTSTDIDTDDKTQLNGGHQGMLLATNCSDQSNVKSSDQRTLRRLAQNREAARKSRLRKKAYVQQLENSRIRLAQLEEELKRARQQGSLVERGVSADHTHLAAGNGVFSFELEYTRWKEEHQRMINDLRSGVNSQLGDNDLRVLVDAVMSHYDEIFRLKGIGTKVDVFHMLSGMWKTPAERFFMWLGGFRSSELLKILGNHVDPLTDQQLIGICNLQQSSQQAEDALSQGMEALQQSLLETLSSASMGPNSSANVADYMGHMAMAMGKLGTLENFLRQADLLRQQTLQQLHRILTTRQAARAFLVIHDYISRLRALSSLWLARPRD*

**>BnaC06g30310D**

MQSSFKTVPFNPDFYSQASFFFRGDSCLDEFHQPINGFHHDEAVGLSPNVTVAASNNLHYTTFDTVMDYGMRERLEGEDECLDTGELMYQRGTRLVGGGGGEVNSSLDKWCDSVSAMADNSQHTDTSTDIDTDDKSQLNGVHQGMLLATNCSDQSKTLRRLAQNREAARKSRLRKKAYVQQLENSRIRLAQLEEELKRARQQGCSSERGVSRENTHVAAGNGVFSFELEYARWMEEHQRLINDLRAGVHSQLGDNELRVLVDAVMSHYDEIFRLKGIGTKVDVFHMLSGMWQTPAERFFMWLGGFRSSELLKILGNHVDPLTDQQLIGICNLQQSSQQAEDALSQGMEALQQSLLETLSSASMGPNSSANVADYMGHMAMAMGKLGTLENFLRQADLLRQQTLQQLHRILTTRQAARAFLVIHDYICRLRALSSLWLARPRD

**>Csa_6G031950**

MQSFENPEQFYAHSSSSSIFLRGDDSGRFHTRFLPDIEELQQSAVAIAAFHQDDAVDLSSSSVFGLKSTHNTAFPIHLPYGNTDVVSIGRTGYLDTGQELMRLKRVAPPHSLAVTVAASSSLGNGSFENWGESAMADNSQQTDTSTDIDNDERNQFQGAVHGALMAVDSMDQSKAKSADQKTLRRLAQNREAARKSRLRKKAYVQQLENSRQRLAQLEQDLHRARQQQGIFVASGVGDHCASMAGNGALAFDLDYARWLDEHQRLINDLRALANSQLGDDELRFLVDGVMTHYDELFRLKSVGAKADVFHILSGMWKTPAERCFMWLGGFRSSELLKIVGSHLEPLTDQQLMGICNLQQSSQQAEDALSQGIEALQQSLVETLSSASLGPASSGNVADYMGQMAIAMSKLTTLENFLHQADLLRQQTLQQMHRILTTRQAARALLVISDYISRLRALSSLWLARPKE

**>GLYMA_02G097900**

MKNMQGFKTAQQPSSQQQLYCHSSFLLRGNDPNRNTTRFSDLGELHHSSSVFHHEDAADLSSSSMFSVKSNNVVGGSNIQYGTLNTLSTNVGSAEIGTSVRGCMDTGQQLMYHKGVTTAALPLGNGQVENWDDSGLADNSQQTDHTSTDIDTDDIIQCNRVKNGTRMVVHSKDATKVKPGDQKTLRRLAQNREAARKSRLRKKAYVQQLESSRVKLVHLEQELQRARQQGIFIATPGDQGHLAVGNGALAFDIDYAHWVDEHQRLLNDLRTAINSQMSDSDLHILVDSVMAHYNELFRLKSIGAKADVLHIHNGMWKTPVERCFMWLGGLRSSELLKIIKNHLEPLTDQQLMGICNLQQSSQQAEDALNQGMEALQQSLVEILSSTSLGPNGSGNVADYMGQMALAMGKLAVLGSFLHKADLLKQETLEQLQRILTTRQTARALLVQNDYISRLRALSSLWLARPRE

**>GLYMA_01G084200**

MPFSSQRHSRIFLPSHTSLFLFSLMKNMQGFKTAQPSSQQLYCHSSFLLRGNDPNQNPARFSDLGEHHHFSAVFHQEDAADLSSSSMFSVKSSNVVGGSNMQYGTLNTNVGHAEIGSSGGGCMDTRQQLMYHKGVTMAALPLGNGQVENWADSGKADNSQQTDDTSTDIDTDDIIPCNRVKNGTRMVVHSKDETKVKPGDQKTLRRLAQNREAARKSRLRKKAYVQQLESSRVKLVQLEQELQRARQQGIFIATPGDQGHLAVGNGALAFDIDYAHWVDEHQRLLNDLRTAVNSQMSDSDLHILVDSVMAHYNELFRLKSLGTKADVLHIHNGMWKTPVERCFMWLGGFRSSELLKIIKNHLEPLTDQQLMGIYNLQQSSQQAEDALSQGLEALQQSLVETLSSISLGPTDSGNVVDYMGQMALAMGKLADLESFVRQADLLKQQTLQQLQRILTTRQTARALLVQTDYISRLRALSSLWLARPRE

**>GLYMA_10G276100**

MQSFNTTESESTSPLYSHSPFYHRGNDPSRNPTRFSDLQHSFRQHDAVDLSSSSVFGAKSSNVAVVASNLQCGTFNTNLGCAEFGSIEQRGMFQRGTNSATTVSMGNRHVENWAEDSQHTEDTCTDIDTDDKNQCFSTVSWCNGVGDGALVVVDSQDQSKTKVKAEDQKTVRRLAQNREAARKSRLRKKAYVQQLETSRVRLAQLEQELQRARQQGAFIATGNQGDRSHSAVGNGALAFDMDYARWFDEHQRLINDIRSAINSQMDENELHLLVDGVMAHYDELFRLKSIGAKADVFHILSGMWKTPAERCFIWLGGFRSSELLKIVRNQLEPLTEQQLMGIYNLQQSSQQAEDALSQGMDALQQSLSETLSSSSLGPSGSGNVAEYMGQMAIALGKLATLENFLHQADLLRQQTLQQMRRILTTFQAARALLVINDYVSRLRALNSLWLACPREY

**>GLYMA_20G113600**

MQSFNTTTESESISPLYCHSPFFHRGNDTSRNPSRFSDLQHSFRQHDAVDLSSSSVFGGKSSNVAVVASNLQCGTFNTNLGCAEFGSTEQGPMFQRGTTVSMATGNQHVENWADDSHPTEDTCTDIDTDDKNQCFSTVSWCHGVGDGALVVVDSHDQSKTKVKAEDQKTVRRLAQNREAAKKSRLRKKAYVQQLENSRVRLAQLEQELQRARQQGAFIATGIPGDRGHSSVANGALAFDMDYARWVDEHQRLIIDIRSAINSQMGENELHLLVDGAMAHYDELFRLKSIGAKVDVFHILSGMWKTPAERCFIWLGGFRSSELLKIVRNQLEPLTEQQLMGIYNLQQSSQQAEDALSQGMEALQQSLSETLSSSSLGPSGSENVAEYMGQMAIALGKLATLENFLHQADLLRQQTLQQMRRILTTCQAARALLVINDYVTRLRALNSLWLACPREY

**LATERAL ORGAN FUSION 1 (LOF1)**

**>AT1G26780 (AtLOF1)**

MFITEKQVWMDEIVARRASSSWDFPFNDINIHQHHHRHCNTSHEFEILKSPLGDVAVHEEESNNNNPNFSNSESGKKETTDSGQSWSSSSSKPSVLGRGHWRPAEDVKLKELVSIYGPQNWNLIAEKLQGRSGKSCRLRWFNQLDPRINRRAFTEEEEERLMQAHRLYGNKWAMIARLFPGRTDNSVKNHWHVVMARKYREHSSAYRRRKLMSNNPLKPHLTNNHHPNPNPNYHSFISTNHYFAQPFPEFNLTHHLVNNAPITSDHNQLVLPFHCFQGYENNEPPMVVSMFGNQMMVGDNVGATSDALCNIPHIDPSNQEKPEPNDAMHWIGMDAVDEEVFEKAKQQPHFFDFLGLGTA*

**>BnaA08g19860D**

MFISEKQRWTDEVAAARREASSWDFPFNDINVQHHRRCNTSHEFDILKSPLGDNVEGSNNHNPNYSNNESGKKETTDSGQSWSSSSSKPSVLGRGHWRPAEDVKLKELVAIYGPQNWNLIAEKLQGRSGKSCRLRWFNQLDPRINRRAFTEDEEERLMQAHRLYGNKWAMIARLFPGRTDNSVKNHWHVIMARKYREHSSAYRRRKLMINNPLKPQLSNHPSPNPNHHSFISTHHYFTQPFHEFNLTHHLVNNASITPDHNQLVLPIHCFQGYESNETPMVVSMFGNQIMGGDNVDVMSEGLYEYPYIDSTSHEKGVPNEPMSWIGMEGEDEEVVEKAKQQPHFFDFLGLGTA

**>BnaCnng12230D**

MFILEKQWRTDEVAAARREASPWDFPFNDINVQHHRRCNTSHEFDILKSPVGDNVEGSNNHNPNYSNNESGKKETTNSGQSWSSSSSKPSVLGRGHWRPAEDVKLKELVAIYGPQNWNLIAEKLQGRSGKSCRLRWFNQLDPRINRRAFTEDEEERLMQAHRLYGNKWAMIARLFPGRTDNSVKNHWHVIMARKYREHSSAYRRRKLMINNPLKPHLSNHPSPNPNHHSFISTHHYFTQPFHEFNLTHHLVNNASITPDHNQLVLPFHCFQGYESNETPMVVSMFGNQIMGGDNVGVMSEGLYDYPYIDSTSHEKGVPNEPRSWIGMEGEDEEVVETAKQQPHFFDFLGLGTS

**>BnaA09g29250D**

MIARLFPGRTDNSVKNHWHVIMARKYREHSSAYRRRKLMINNPLKPHLPNHRSPNCPSFISTKHYFTRPFHEFNLTHHLVNHATMTADQNHLVLPFHCFQGYESTEAPTVVSMFGNQMMIEDNVGATSEALYNFPYVDSMSQEKGVPNEAMNLIGMEVVDEEVAELKGKAATTFF

**>Csa_5G605730**

MGMFIDEDEESFNLDLNSAAIFSSSSQESCEEINGRAFWNFPFSCESDIVNNNNNNNNGGSEFSDGLNQNAANNPTSCSNNTPSSSTGAQSRLCARGHWRPAEDTKLRELVALYGPQNWNLIAEKLEGRSGKSCRLRWFNQLDPRINRRAFSEEEEERLMQAHRIYGNKWAMIARLFPGRTDNAVKNHWHVIMARKYREQSRCYRRRKLSQSVYRKMEEDLSFLNIPKDHNHHDTITTAAAATTITTTSSSSFGNFEGCVDYGFLRQMVIGGGGETLSSNYNTTPNNPYFNSCAHLSTLNVLPDPKSRFWEGTSNGFLVPRSHGHHQYETYNTAVPPSNGGVEGSSSVTVEGRKQSPRFIDFLGVGATT

**>GLYMA_01G049600**

MASSKCCMISPQQNQNAPTCYPSPIGVVFADMMGSLSLATVSNNASSSQESNVYGYGYGYASGVGNGSTRWSFPFMREFLSSNFEDCSDLVGAGESNNSNEKTNHNNGKFSEEESNPNENHANGKEVDSGHSKLCARGHWRPAEDSKLKELVALYGPQNWNLIAEKLEGRSGKSCRLRWFNQLDPRINRRAFSEEEEERLMQAHRIYGNKWAMIARLFPGRTDNAVKNHWHVIMARKYREQSSAYRRRRMSQSVHRRVEQNPTFVCSSSRDNASTTEQEPEPSPSYYPNLIHHGVALTNNMASFPPFHGVAACNGVVVEFGSNGSPQNMTSGREAMPNTTHVGLSAQAQQQAPFDFFSGGGSNDIVLESISHMRSRERTNGSHNHHCQLSGCYPHYPQQYLMAMQQQLDNNNNFYSFLNSSPAASTAREPSSSPCVAEIRDKVENSDPPDGVPPPFFDFLGVGAT

**>GLYMA_07G228700**

MGMVYADMDSLSLCSNYGVVSSHQDNCYVSNGGTNWGFPFMRECPNFEDHPNNSDDVAEEGKGSYSSGEDSDKVNHNANNFNDENSPNENSNSGGSGHSKLCARGHWRPAEDSKLKELVALYGPQNWNLIAEKLEGRSGKSCRLRWFNQLDPRINRRAFSEEEEERLMQAHRIYGNKWAMIARLFPGRTDNAVKNHWHVIMARKYREQSSAYRRRLSQSSSVYRRVEELNTTTTTSSTLVSRNTAAAEQQPPPPYCLNLPNNGGLANNINMPSSPYAATPFHGGVVAVAPSSGVEFGLLNGSPHMTAAKEAFSTPKLVPHIALYPQQTPLDFFSGVRSSNDMVGEYFGQNDHHQPSFGFNPQYPQYVMLMQQHQQNNHNYGFSNSTAQILGHSEASLSSVAAEHRDHQNVSSDQCPPDATTTIPPPFIDFLGVGAT

**>GLYMA_02G108800**

MASSKCCMISPQQNQNAPTCYPSPMGMVYADMMGSLSLATVSNNASSSQESNGYSYGYASGVENARSTRWSFPFTREFLSSNFEDCSDVVVGAGDSNNNINEKTNHNNGKFSEEESNPNENNPAGGKEVDSGHSKLCARGHWRPAEDSKLKELVALHGPQNWNLIAEKLEGRSGKSCRLRWFNQLDPRINRRAFSEEEEERLMQAHRIYGNKWAMIARLFPGRTDNAVKNHWHVIMARKYREQSSAYRRRRMSQPVYRRVDQNPTFVCSSRDNNASTTEPEPESSPSYYPNLNDRGVALTNNMASFPSLHGVAAGAGGVVEFGSNVSPLNMTRGREAVSNTTHVGLCAQAQQQAPFDFFSGGGSHDMVLESMSQMRSRERTNESYNHHCQLSGFYPHHPQQYLMAMQQQLDNNNFYSFLNSSSSASTTRTREPSSPCVAEIRDKVVNSDPPDGVPPPFFDFFGVGAT

**>GLYMA_20G034100**

MKAWPARVIYPSSQENTNSLTRYPSSSSHSSSPPSMGMVYADMDSLSLCSHYGTVVSSHHQDCYVSNGGTNWGFPFMRECPNNSDDVAEEEEGKGSDSSTGEDSDKVNHHANNSNDENNPNENNSNSGGSGHSKLCARGHWRPAEDSKLKELVALYGPQNWNLIAEKLEGRSGKSCRLRWFNQLDPRINRRAFSEEEEERLMQAHRIYGNKWAMIARLFPGRTDNAVKNHWHVIMARKYREQSSAYRRRRLSQSSSVYRRVDEINTTITTTNSIVVSRDTEQQAPPPPPYCVNLSNNGGLANNIINNNNNINMMSSFPYAAASFHGGVVPSSGGIEFGLNSSPHMTTAKEPFSTTKLAPPHIALYPQQTSLDFFSGVRSSSDMVGEYFGQNLHHQQQQPSSGFYPQYPQYVMLMNHHHQQNNHNNFYGFSNSTSQILLGHSEASLSSSVAAEEHRDHQNLSSDQCPPDATTTIPPPFIDFLGVGAT

**CUP-SHAPE COTYLEDON 1 (CUC1) and CUC2**

**>AT3G15170 (AtCUC1)**

MDVDVFNGWGRPRFEDESLMPPGFRFHPTDEELITYYLLKKVLDSNFSCAAISQVDLNKSEPWELPEKAKMGEKEWYFFTLRDRKYPTGLRTNRATEAGYWKATGKDREIKSSKTKSLLGMKKTLVFYKGRAPKGEKSCWVMHEYRLDGKFSYHYISSSAKDEWVLCKVCLKSGVVSRETNLISSSSSSAVTGEFSSAGSAIAPIINTFATEHVSCFSNNSAAHTDASFHTFLPAPPPSLPPRQPRHVGDGVAFGQFLDLGSSGQIDFDAAAAAFFPNLPSLPPTVLPPPPSFAMYGGGSPAVSVWPFTL*

**>AT5G53950 (AtCUC2)**

MDIPYYHYDHGGDSQYLPPGFRFHPTDEELITHYLLRKVLDGCFSSRAIAEVDLNKCEPWQLPGRAKMGEKEWYFFSLRDRKYPTGLRTNRATEAGYWKATGKDREIFSSKTCALVGMKKTLVFYKGRAPKGEKSNWVMHEYRLEGKFSYHFISRSSKDEWVISRVFQKTTLASTGAVSEGGGGGGATVSVSSGTGPSKKTKVPSTISRNYQEQPSSPSSVSLPPLLDPTTTLGYTDSSCSYDSRSTNTTVTASAITEHVSCFSTVPTTTTALGLDVNSFSRLPPPLGFDFDPFPRFVSRNVSTQSNFRSFQENFNQFPYFGSSSASTMTSAVNLPSFQGGGGVSGMNYWLPATAEENESKVGVLHAGLDCIWNY*

**>BnaC03g38960D**

MDMDVFNGWERSRYEDETVMPPGFRFHPTDEELITYYLLKKVLDSSFSCAAISQVDLNKSEPWELPEKAKMGEKEWYFFTLRDRKYPTGLRTNRATEAGYWKATGKDREIKSSKTNSLLGMKKTLVFYKGRAPKGEKSCWVMHEYRLDGKFSYHYITSSAKDEWVLSKVCLKSSVVSRETKLISSSGGVNCSSSSASAGSLIAPMIDAYATEHVSCFSNTSAAHADASFPPTYLPAPPPPSLPRQPRRFGDDVAFGQFMDVGASGQFSIDAAFLPNLPSLPPTVFTAPSQPFGVYGGGSAVSSWPFAL

**>BnaA03g33770D**

MDMDVFNGWERSRYEDETVMPPGFRFHPTDEELITYYLLKKVLDSSFSCAAISQVDLNKSEPWELPEKAKMGEKEWYFFTLRDRKYPTGLRTNRATEAGYWKATGKDREIKSSKTNSLLGMKKTLVFYKGRAPKGEKSCWVMHEYRLDGKFSYHYITSSAKDEWVLSKVCLKSSVVSRETKLISSSGGVNCSSSSSAAGSLIAPMIDAYATEHVSCFSNTSAAHADASFPPAYLPAPPPPPSLPRQPRCFGNDVAFGQFMDVGASGQFSIDAAFLPNLPSLPPTVFTTPSQPFGMYGGGSAVSSWPFAL

**>BnaC05g38460D**

MKSSSLTSSLRKSLTPTSPVPPFLKLISKPWELPEKAKMGEKEWHFFTRRDRKYPTGLRTNRATEAGYWKATGKDREIKSSKTNSLLGMKKTLVFYKGRAPKGEKSCWVMHEYRLDGKFSYHYITSSAKDEWVLSKVCLKSSVVSRETKLVSSSSVSVTSISCSSSTGSLIAPVIDAFATEHVSCFSNTSASHVDASFPIYLPAPPQSLPRQPRRIDDVAFGQFMGLGSTGQFNIDAAFLPNLPSLPPTFLHPPPQYYAPYGGAAVSSWPFAL

**>BnaA02g09970D**

MDIPYYHYDHGGDSQYLPPGFRFHPTDEELITHYLLRKVIEGCFSSRAIAEVDLNKSEPWQLPGKAKMGEKEWYFFSLRDRKYPTGLRTNRATEAGYWKATGKDREIYSSKTCALVGMKKTLVFYKGRAPKGEKSSWVMHEYRLEGKFSYHFISRSSKDEWVISRVFKKPGLANTGGSAEASISVSNGTGTSKKTKIPSNISTNYREQPSSPSSVSLPPLLDPTTTLGYTDSSWSYDSRSTNTPVITTAITEHVSCFSTATTTTALGLDVDVDSFNHLLPPVPPGFDPFPRFVSRNVSSLSNFRSFQENFNHFPYYGSSSASTMTTPVNLPSSHGGTGMNYWLQTTAEENETKAGLLNGGLDCVWNY

**>Csa_4G629480**

MDVSSFSHYDSAASDNSNHHLPPGFRFHPTDEELITYYLLKKVLDTNFTGRAIAEVDLNKCEPWELPEKAKMGEKEWYFFSLRDRKYPTGLRTNRATEAGYWKATGKDREIYSVKTCSLVGMKKTLVFYRGRAPKGEKSNWVMHEYRLEGKFAYHYLSGSSKDEWVISRVFQKSGSGCGGATSSNGGSSKKARFIPGSINVYPEPSSPSSVSLPPLLDSSLYSAPATGTAASGNITDRDSCSYNSPTAREHVSCFSTTTAGGSFNMPNYDFATPSPLLAADPSPRFHRNIGLSAFPSLRSLQENLQLPFFYSPVNLCSPPVPVLNNGNDVAGCSSGGNWGVSDEPKVMNSTELDCMWSY

**>GLYMA_13G274300**

MDNSSYHHLDHTEAHLPPGFRFHPTDEELITYYLLKKVLDSTFTGRAIAEVDLNKSEPWELPEKAKMGEKEWYFFSLRDRKYPTGLRTNRATEAGYWKATGKDREIYSSKTCSLVGMKKTLVFYRGRAPKGEKSNWVMHEYRLEGKFAYHYLSRNSKDEWVISRVFQKSNTATNNGGSVMSASSNSKKTRMNSTTSLIHEPSSPSSVFLPPLLDTSPYTNTANFTDRHNGSYDSITKKEHVSCFSTIAAATTAVVSPNNFNNAGFDLSPSQPLATDPFARFQRNVDFSAFPSLRSLQDNLQFPFVFSTAAPPFSGGGSGDFLSWPVPEEQRLIDGVSNMPLGVSELDCMWSY

**>GLYMA_12G226500**

MDHTEAHLPPGFRFHPTDEELITYYLLKKVLDSTFTGRAIAEVDLNKSEPWELPEKAKMGEKEWYFFSLRDRKYPTGLRTNRATEAGYWKATGKDREIYSSKTCSLVGMKKTLVFYRGRAPKGEKSNWVMHEYRLEGKFAYHYLSRNSEDEWVISRVFRKSNTTPITNGGSTMSASTNSKKTRINNTTSLIHEPGSPSSVFLPPLLDSSPYTNTTTNTFTDHHNSSYDSATKKEHVSCFSTIAAATAVVSPNNNFNNASFDLPPSQPLATDPFARFQRNVGLSAFPSLRSLQDNLQLPFFFSTAAAPPFSGGGSGDFLSWPVPEDGVSNMPLGVSELDCMWGY

**>GLYMA_12G161700**

MDSYYHQHHNPHFDNNNEPHLPPGFRFHPTDEELITYYLLKKVLDSSFTGRAIVEVDLNKCEPWELPEKAKMGEKEWYFYSLRDRKYPTGLRTNRATEAGYWKATGKDREIYSSKTCSLVGMKKTLVFYRGRAPKGEKSNWVMHEYRLEGKFAYHYLSRSSKEEWVISRVFQKNTTGGGSTVSSASAATTGGSSKKTRMTTSNNSSNMSLCPEPGSPSSIYLPPLLESSPYAAAAATFNDRERCSFDSAANNNQREHVSCFSTISGAAFDHLVPSPEPPLDPSARFHRNNNNNVGVGISTFPCLRSLHDNLNLPFFFSPTGHISSAEVASFGAVGNWTAAPEEHRMADSGSGMTIVPSELDCMWDY

**>GLYMA_06G236000**

MDNYYHQHHHPHFDNSNNNEPHLPPGFRFHPTDEELITYYLLKKVLDSSFTGRAIVEVDLNKCEPWELPEKAKMGEKEWYFYSLRDRKYPTGLRTNRATEAGYWKATGKDREIYSSKTCSLVGMKKTLVFYRGRAPKGEKSNWVMHEYRLEGKFAYHYLSRSSKDEWVISRVFQKNTTGGGSTVSAAAAATSGGSSKKTRMTTSNTSSNMSLCPEPGSPSSIYLPPLLESSPYAAASTTTATPAAAAFNDHESCSFNSAVSNNNQREHVSCFSTISAAAFDHLVPPPEPPLDPFARFHCNNNNVGVGVSTFPCLRSLHDNLNLPFFFSPMGHVSSADVASFGAVTNWPAPEEQRMADGGSGMGIVPSELDCMWGY

**INNER NO OUTER (INO)**

**>AT1G23420 (AtINO)**

MNINKTYLSVYIYTHTHTHISPIIHRTHTLSMTKLPNMTTTLNHLFDLPGQICHVQCGFCTTILLVSVPFTSLSMVVTVRCGHCTSLLSVNLMKASFIPLHLLASLSHLDETGKEEVAATDGVEEEAWKVNQEKENSPTTLVSSSDNEDEDVSRVYQVVNKPPEKRQRAPSAYNCFIKEEIRRLKAQNPSMAHKEAFSLAAKNWAHFPPAHNKRAASDQCFCEEDNNAILPCNVFEDHEESNNGFRERKAQRHSIWGKSPFE*

**>BnaC05g18750D**

MPISLTLYLYKLTHTHSHISPIIHRLSLYFSELKSLYMTKIPNMTTLNQLFDLPGQVCHVQCGFCTTILLVSVPFTSLSMVVTVRCGHCTSLLSVNLMKASFIPLHLLTSLSHMDEKGNEEVAATTDGGVEEEAWKVNQEKENSPATLVTSSDSEDEDRDVSRVYQVVNKPPEKRQRAPSAYNCFIKEEIRRLKAQNPSMAHKEAFSLAAKNWANFPPVQNRRAASDQCFCEDDNNALLSCNALGDHDESNNGFRERKAQRHSIWGKSPFD

**>Csa_5G600930**

MPKLLHITLTFHSLYISSVLSYNSWLSFQLTEICLFFCSKTMNHLFDLPEKICYVQCGICTTILLVSVPCSSLSMAVTVTCGHCSSLLSVNMMKATLVPLHFLSSLSHNVPKETYREMNSGKFFDSFKRSNLKFSEYEVEDDLIPVTTPFVNKPPERRQRAPSAYNCFIKDEIRRLKTQNPEMTHKEAFRTAAKNWANFPPIQEKDDKEKCNQIEENGSWNTQIPEVHKEGIHFLT

**>Csa_2G348870**

MSTVEQLLADSSEQIRYVQCGLCSTILLVNVPYSNLSMVVSVRCGNCAGLLSVNMAKPSFIPFDLLTSLSHNLPKEGFGQEFNVQKHYCLDSLSNSSSSSDLLTSYHNIIQNHEDDDVIIIPPTPVVNKPPEKKQRAPSAYNQFIKEEIRRLKAENPAMAHKEAFKTAAKNWAHLPPVNAEGADHMMLRKKNVM

**>GLYMA_08G285200**

MSTLNHLFDLPEQICYVQCGFCTTILMVSVPCSILSTVVTVRCGHCTSLLSVNMKKASFVPFHLLASLTHLEPKEGASDDGANKSLNSYNNASIITTNSDCEEENVTQISNVVHKPPEKRQRTPSAYNRFIKEEIKRLKSENPNMAHKEAFSTAAKNWANFPPSQCDGEADSCNGTEQLGDLDSHEEPRDAIEVHKEGQGFRGRKAPRNSIWERAPFE

**>GLYMA_18G140400**

MSTLNHLFDLPEQICYVQCGFCTTILMVSVPCSILSTVVTVRCGHCTSLLSVNMKKASLVPFHLLASLTHLEPKEGASEDGANKSLSSYNTSTMTNSDCEEENVTQISDFVHKPPEKRQRTPSAYNRFIKEEIKRLKAENPNMAHKEAFSTAAKNWANFPPSPSDGEADSCNGTEQLVDLDSHQEPRDAIEVDKEGQGFRGRKAPRNSIWERTPFEIKEEIKRPKVENPEMAHKEAFSTTTKNWANFPQTQWCKGDEESCSQTKQLVDLD

**AGAMOUS (AG)**

**>AT4G18960 (AtAG)**

TAYQSELGGDSSPLRKSGRGKIEIKRIENTTNRQVTFCKRRNGLLKKAYELSVLCDAEVALIVFSSRGRLYEYSNNSVKGTIERYKKAISDNSNTGSVAEINAQYYQQESAKLRQQIISIQNSNRQLMGETIGSMSPKELRNLEGRLERSITRIRSKKNELLFSEIDYMQKREVDLHNDNQILRAKIAENERNNPSISLMPGGSNYEQLMPPPQTQSQPFDSRNYFQVAALQPNNHHYSSAGRQDQTALQLV*

**>BnaC09g24200D**

MFHFVLTQKNRCLLLRFRIKITQLSNVKGTIERYKKAISDNSNTGSVAEINAQYYQQESAKLRQQIISIQNSNRQLMGETIGSMSPKELRNLEGRLDRSVNRIRSKKNELLFAEIDYMQKREVDLHNDNQLLRAKIAENERNNPSMSLMPGGSNYEQIMPPPQTQPQPFDSRNYFQVAALQPNNHHYSSAGREDQTALQLV

**>Csa_6G520410**

MFQNQEEKMSDSPQRKMGRGKIEIKRIENTTNRQVTFCKRRNGLLKKAYELSVLCDAEVALIVFSSRGRLYEYANNSVKATIDRYKKASSDSSNTGSTSEANTQFYQQEAAKLRVQIGNLQNSNRNMLGESLSSLTAKDLKGLETKLEKGISRIRSKKNELLFAEIEYMRKREIDLHNNNQMLRAKIAESERNVNMMGGEFELMQSHPYDPRDFFQVNGLQHNHQYPRQDNMALQLV

**>GLYMA_15G088600**

MVFPNPSMSVSPQKKMGGGKIEIKRIENTTNRQVTFCKRRNGLLKKAYELSVLCDAEVALIVFSSRGRLYEYANNSVKATIERYKKACSDSSGAGSASEANAQFYQQEADKLRAQISSLQNNNRQMMGESLGPLTAKELKNLETKLEKGISRIRSKKNELLFAEIEYMQKREIDLHNNNQLLRAKIAEGERNHHNLAVLPGGSNYDSLQTSQQQFDSRGYFQVTGLQPNNQYARQDQMSLQLV

**>GLYMA_08G120600**

MAFPNQSMSSESPQRKMGRGKIEIKRIENTTSRQVTFCKRRNGLLKKAYELSVLCDAEVALIVFSNRGRLYEYANNSVKASIERYKKASSDSSSGGRSASEANAQFYQQEAAKLRVQISNLQNHNRQMMGEGLSTMNGKDLKNLETKLEKGISRIRSKKNEMLFAEIEHMKKREIYLHNDNQLLRAKIGEGERSHHNVNGLSGTTSYESMQSQFDSRGFFQVTGLQPNNNNQYAGQDMSLQFV

**SEEDSTICK (STK)**

**>AT4G09960 (AtSTK)**

MGRGKIEIKRIENSTNRQVTFCKRRNGLLKKAYELSVLCDAEVALIVFSTRGRLYEYANNNIRSTIERYKKACSDSTNTSTVQEINAAYYQQESAKLRQQIQTIQNSNRNLMGDSLSSLSVKELKQVENRLEKAISRIRSKKHELLLVEIENAQKREIELDNENIYLRTKVAEVERYQQHHHQMVSGSEINAIEALASRNYFAHSIMTAGSGSGNGGSYSDPDKKILHLG*

**>BnaA03g24210D**

MGRGKIEIKRIENSTNRQVTFCKRRNGLLKKAYELAVLCDAEVALIVFSTRGRLYEYGNNNIRATIERYKKASSDNANTHSVQEINAAYYQQESAKLRQQIQTIQNSNRNLMGDSLSALNVKELKQVENRLEKAISRIRSKKHELLLAEIENLHKREIKLDNESIYLRTKIAEVERFQQHHHQMVSGTEMTAIEALASRNYFAHNIMTIGSGSGAGHGCSYFDPDKKTHLG

**>BnaC03g72890D**

MRSNRTIMGEGEWQFRTTKNRFHVITGENTVVRKVTTTAPCNYYGFKEALHILCTLIGRGKIEIKRIENSTNRQVTFCKRRNGLLKKAYELAVLCDAEVALIVFSTRGRLYEYANDNIRATFERYKNSSSGNANTHSVQEINAAYYQQESAKLRQQIQTIQNSNRLAAFSGIFINSLSALNVKELKQVENRLEKAISRIRSKKHELLLAEIENLHKREIKLDNESIYLRTKIAEVERFQQHHHQMVSGTEMTAIEALASRNYFAHNIMTIGSGSGAGHGCSYSDPDKKTHLG

**>Csa_6G520410**

MGRGKIEIKRIENTTNRQVTFCKRRNGLLKKAYELSVLCDAEVALIVFSSRGRLYEYSNNSIKTTIERYKKACSDSSATSSVTELNTQYYQQESAKLRQQIQMLQNSNRHLMGDSLSALTVKELKQLENRLERGITRIRSKKHEMLLAEIEYLQKREIELENENVCIRTKIAEVERVQQANMVSGQELNAIQALANSRNFFSPNIMEPAGPVSYSHQDKKMLHLG

**>GLYMA_06G324400**

MGRGKIEIKRIENTTNRQVTFCKRRNGLLKKAYELSVLCDAEVALIVFSSRGRLYEYSNNNIRSTIERYKKACSDHSSASTTTEINAQYYQQESAKLRQQIQMLQNSNRHLMGDALSTLTVKELKQLENRLERGITRIRSKKHEMLLAEIEYFQKREIELENENLCLRTKITDVERIQQVNMVSGPELNAIQALASRNFFNPNMLEGGTVYPHSDKKILHLG

**>GLYMA_04G257100**

MGRGKIEIKRIENTTNRQVTFCKRRNGLLKKAYELSVLCDAEVSLIVFSSRGRLYEYSNNNIRSTIERYKKACSDHSSASTTTEINAQYYQQESAKLRQQIQMLQNSNRHLMGDALSTLTVKELKQLENRLERGITRIRSKKHEMLLAEIEYFQKREIELENENLCLRTKITDVERIQQVNMVSGPELNVIQALASRNFFNPNMLDGGTVYPQTDKKILHLG

**SHATTER-PROOF 1 (SHP1) and SHP2**

**>AT3G58780 (AtSHP1)**

MEEGGSSHDAESSKKLGRGKIEIKRIENTTNRQVTFCKRRNGLLKKAYELSVLCDAEVALVIFSTRGRLYEYANNSVRGTIERYKKACSDAVNPPSVTEANTQYYQQEASKLRRQIRDIQNSNRHIVGESLGSLNFKELKNLEGRLEKGISRVRSKKNELLVAEIEYMQKREMELQHNNMYLRAKIAEGARLNPDQQESSVIQGTTVYESGVSSHDQSQHYNRNYIPVNLLEPNQQFSGQDQPPLQLV*

**>AT2G42830 (AtSHP2)**

MEGGASNEVAESSKKIGRGKIEIKRIENTTNRQVTFCKRRNGLLKKAYELSVLCDAEVALVIFSTRGRLYEYANNSVRGTIERYKKACSDAVNPPTITEANTQYYQQEASKLRRQIRDIQNLNRHILGESLGSLNFKELKNLESRLEKGISRVRSKKHEMLVAEIEYMQKRVKEIELQNDNMYLRSKITERTGLQQQESSVIHQGTVYESGVTSSHQSGQYNRNYIAVNLLEPNQNSSNQDQPPLQLV*

**>BnaA05g02990D**

MEGGASDEVAESSKKIGRGKIEIKRIENTTNRQVTFCKRRNGLLKKAYELSVLCDAEVALVIFSTRGRLYEYANNSVRGTIERYKKACSDAVNPPSVTEANTQYYQQESSKLRRQIRDIQNLNRHILGESLGSLNLKELKNLEGRLEKGIGRVRSKKHEMLVAEIEYMQKREIELQNDNMYLRSKINERAGMQQQEASVIHQQGTVYESSSHQSEQYNRNYIPVNLLEPNQNSSDQNQPPLQLV

**>BnaA05g03000D**

MEGGASDEVAESSKKIGRGKIEIKRIENTTNRQVTFCKRRNGLLKKAYELSVLCDAEVALVIFSTRGRLYEYANNSTAYLYVSMYVSST

**>BnaUnng05090D**

MEGGASDEVAESSKKIGRGKIEIKRIENTTNRQVTFCKRRNGLLKKAYELSVLCDAEVALVIFSTRGRLYEYANN

**>Csa_6G177190**

MGRGKIEIKRIENTTNRQVTFCKRRNGLLKKAYELSVLCDAEVALIVFSTRGRLYEYANNSVRGTIERYKKAFADSSNSGLSVAEANVQFYQQEATKLKRQIREIQNSNRHILGEALSSLPLKELKSLEGRLERGISKVRAKKNETLFAEMEFMQKREMELQSHNNYLRAQIAEHERIQQQQQQQQQTNMMQRATYESVGGQYDDENRSTYGAVGALMDSDSHYAPQDHLTALQLV

**>GLYMA_14G027251**

MEFPNEAIPEGCSQKKTGRGKIEIKRIENTTNRQVTFCKRRNGLLKKAYELSVLCDAEVALVVFSSRGRLYEYANNSVRGTIDRYKKACAASTNPESVSEANTQFYQQEASKLKRQIRDIQNLNRHILGEALSSLSLKELKNLESRLEKGLSRVRSRKHETLFADIEFMQKREIELQNHNNFLRAKIAEHEKAQQRQQDMIPGNVCESTIPPQSYDRNFFPVNLIDSNNQYSNQDQTALQLVLSSGVGSLTSSYVS

**>GLYMA_02G287700**

MEFPNEAIISEGSNSQKKTGRGKIEIKRIENTTNRQVTFCKRRNGLLKKAYELSVLCDAEVALVVFSSRGRLYEYANNSVRGTIERYKKACAASTNAESVSEANTQFYQQEASKLKRQIRDIQNLNRHILGEGLSSLSLKELKNLESRLEKGLSRVRSRKHETLFADIEFMQKREIELQNHNNFLRAKIAENERAQQRQQDMIPGTECESTIPNSQSYDRNFFPVNLIDSNNNQYSRQDQTALQLV

**>GLYMA_18G105800**

MLSTMEDPNQAQEGSSQKKMGRGKIEIKRIENTTNRQVTFCKRRNGLLKKAYELSVLCDAEVALVVFSTRGRLYEYANNSVRATIERYKKANAAASNAESVSEANTQFYQQESSKLRRQIRDIQNLNRHILGEALGSLSLKELKNLEGRLEKGLSRVRSRKHETLFADVEFMQKREIELQNHNNYLRAKIAEHERAQQQQSNMMSGTLCESLPSQSYDRNFFPVNLIASDDQQQYSSQDHTALQLV

**>GLYMA_08G310100**

MLPTMEDPNQAPEASSQKKMGRGKIEIKRIENTTNRQVTFCKRRNGLLKKAYELSVLCDAEVALVVFSTRGRLYEYANNSVRATIERYKKANAAASNAESVSEANTQFYQQESSKLRRQIRDIQNLNRHILGEALGSLSLKELKNLEGRLEKGLSRVRSRKHETLFADVEFMQKREIELQNHNNYLRAKIAEHERAQQQQSNMNMSGTLCESLPSQSYDRNFFPVNLIASDDQQQYSRQDHTALQLV

**NEW ENHANCER OF ROOT DWARFISM 1 (NERD1)**

**>AT3G51050 (AtNERD1)**

MRKRDLAILMLSGFAIFFTLQHEGDFAFKEAWFHLYDEYPVKYEADRLPPPIVADLNGDGKKEVLVATNDAKIQVLEPHSRRVDEGFSEARVLAEITLLPDKIRVASGRRAVAMATGVIDRYYKNGTPQKQVVVVVTSGWSVLCFDHNLKKLWETNLQEDFPHNAHHREIAISISNYTLKHGDTGLVIVGGRMEMQPYNHMDPFEELGMTAQNADQHRRSATENQASEDSGAINLRHFSVYAFAGKTGLLRWSKKTDDVEAHTSDASQLIPQHNYKLDVHALNSRHPGEFECREFRESILSVMPHRWDRREDTLLKLAHFRRHKRKTLKKQAGSKSTAYPFHKPEEHTPAGKDLSRKIPKLIGKAARYAGSAKPKKGMQYIPTITNYTKLWWVPNVVVAHQKEGIEAIHLPTGRTLCKLSLLEGGLHADINGDGVLDHVQTVGGNVGERTVVSGSMEVLKPCWAVATSGVPIREQLFNVSICHHSPFNFLHYGGDYSRHFAQARDTSTLEIATPILIPRDDGHKHRKGSHGDVIFLTNRGEVTSYTPDVHGHDAVWQWQLQTEATWSNLPSPSGLTESGTVVPTLKPFSLRIHDNQPMILAGGDQAAVIISPGGSILASIELPSQPTHALITDDFSNDGLTDVIVMTSNGVYGFVQTRQPGALFFSSLVGCLLVVMAVIFVTQHLNSIQGKPRPSSSF*

**>BnaC07g32030D**

MKRLIRHHFHRILTQGRLVLDDSSLPHLSLAPTDANLPLIDYICSDVKTKMRKRDLAILMLSGFAIFFTLQHEGDFAFKEAWFHLYDDYPVKHESDRLPPPLVADLNGDGKKEVLVATNDAKIQVLEPHSRRVDEGFSEARVLADISLLPDKIRVASGRRAVAMATGVIDRYYKDGTPQKQVLVVVTSGWSVLCFDHNLKKLWETNLQEDFPHNAHHREISISISNYTLKHGDTGLVIVGGRMEMQPYNHMDPFEELGITEQNAEKHRRSATEKQPTEDTGGVNLRHFSVYAFAGRTGVLRWSKKTDDVEAHTSDASQLVPQHNYKLDVHSINSRHPGEFECREFRESILSVMPHHWDRREDTLLKLAHFRRHKRKTLKKQAGKSTTFPFQKPEEHTPAGKDLSRKIPKLIGKAARYAGSAKPKKGMQYIPTITNYTKLWWVPNVVVAHQKEGIEAIHLPTGRTLCKLHLLEGGLHADINGDGVLDHVQAVGGNVGERTVVSGSMEVLKPCWAVATSGVPVREQLFNVSICHHTPFNFMHYGEFSRNFAQARDTSSLEIATPILIPRDDGHKHRRGSHGDVIFLTNRGEVTSYTPDMHGREPLWQWQLQTEATWSNLPSPSGLTESGTVVPTLKPFSLRIHDNQPMILAGGDQAAVIISPGGSVLASIELPSLPTHALITDDFSNDGLTDVIVMTSNGIYGFVQTRQPGALFFSSLVGCILVVMAVIFVTQHLNSVKGKPRPSSSFI

**>BnaA03g41130D**

MRKRDLAILMLSGFAIFFTLQHEGDFAFKEAWFHLYDDYPVKHESDRLPPPLVADLNGDGKKEVLVATNDAKIQVLEPHWRRVDEGFSEARVLADISLLPDKIRVASGRRAVAMSTGVIDRYYKDGTPQKQVLVVVTSGWSVLCFDHNLKKLWETNLQEDFPHNAHHREISISISNYTLKHGDTGLVIVGGRMEMQPYNHMDPFEELGITEQNAEKHRRSATEKQPTEDSGGVNLRHFSVYAFAGRTGVLRWSKKTDDVEAHTSDASQLVPQHNYKLDVHSINSRHPGEFECREFRESILSVMPHHWDRREDTLLKLAHFRRHKRKTLKKQAGKSTTFPFHKPEEHTPAGKDLSRKIPKLIGKAARYAGSAKPKKGMQYIPTITNYTKLWWVPNVVVAHQKEGIEAIHLPTGRTLCKLHLLEGGLHADINGDGVLDHVQAVGGNVGERTVVSGSMEVLKPCWAVATSGVPVREQLFNVSICHHTPFNFMHYGEFSRNFAQARDTSSLEIATPILIPRDDGHKHRRSHGDVIFLTNRGEVTSYTPDVHGREPLWQWQLQTEATWSNLPSPSGLTESGTVVPTLKPFSLRIHDNQPMILAGGDQAAVIISPGGSVLASIELPSQPTHALITDDFSNDGLTDVIVMTSNGIYGFVQTRQPGALFFSSLVGCILVVMAVIFVTQHLNSVKVTRPTPLPPPPPFFFISFSLPSPTSPPNSSLTQEMESVELSLTNMETAVNADGAQNGDEFSVDDLLDFSSNDDVFFEDGAELKTQRNKGVSVSSNDETTPDRSNDFPTACELAVPTDDLAELEWLSNFVDDSFAPYSAPTKKPVWLTVDRRHPVTPVNVGSCFKAPLPVKIRTKRPRTGVNLWSSLTDSPSSSPTSSSSSSSGYSSPLWLSGAEFLDEKAVKRQKNKKKKEFLSWEAQSQTRRCSHCGVQKTPQWRAGPLGAKTLCNACGVRFKSGRLLPEYRPACSPTFSSELHSNHHRKVIEMRQKKETSRDADEPGMNRTVQAVQSF

**>Csa_6G504640**

MRKRDLAILMLSAFAIFFSLQHEGDFSFREAWMHLTDEYPIKYEGDRLPPPVVADLNGDGKKEVLVATHDAKILVLEPHSRRVDEGFSHARVLTEASLLPAKVRISSGRRPVAMATGVIDRHPRQGQPVTQVLVVVTSGWSVLCFDHNLNKLWEANLQEDFPHNAHHREIAISITNYTLKHGDSGLIIVGGRMEMQSHIFMDPFEEIGIAEKNAEQHRRSATEKEASENSGSIDLRHFAFYAFAGRSGLPRWSRKNEVNIEAHSSDASQLIPQHNYKLDVHSLNARHPGEFECREFRESILGVMPHHWDRREDTVLELAHFRRHKRKALKKTSGKSVNYPFHKPEENHPPGKDSSKRIPKIIGTAANIAGSAKTKKPLPYVPTITNYTKLWWLPNVVVAHQKEGIEALHLASGRTICKLHLQEGGLHADINGDGVLDHVQAVGGNGAERTVVSGSMEVIQPCWAVATSGVPVREQLFNASICHFSPFNYFQHGELSRFGRTPDMASLEVATPILISRKDGHRHRKGSHGDVVFLTNRGEVTSYSPGLHGHGADWQWQITTGATWSNLPSPSGMMDAGTVIPTLKAIDLRVGATQEMVLAAGEQEAVVISPGGSVQASIELPASPTHALITEDFSNDGLTDIILVTSTGVYGFVQTRQPGALFFSTLVGCLILVMGVIFVTQHLNSIKGKPRPSATR

**>GLYMA_11G072700**

MRKRDLAILMLSAFAIFFTLQQDGGISFKDAWMHLTDEYPIKYEAERLPPPLVADLNGDGKKEVLVATHDAKIQVLEPHSRRVDEGFSEARVLAEVSLLPDKVRVMTGRRPVAMATGYIDRYKIGQPQKQVLVVVTSGWSVMCFDSNLQKLWENNLQEDFPHNAHHREVAISISNYTLKHGDTGLIIVGGRMEMQPHIFMDPFEEMGMGARFAEQHQRSAAEKEASGTVDLRHFAFYAFAGRSGDERWSRKNENIEAHSSDASQLLPQHNYKLDVHALNTRQPGEFECREFRESILGVMPHQWARREDTLFKLAHFRRHKRKALKKTPGKAISYPFHKPEENHPPGKDSTKKISNIIGKAASYAGSAKSKKHLPYVPTITNYTQVWWVPNVVVSHQKEGIEALHLATGRTICKFHLQEGGLHADVNGDGVLDHVQAVGGNGAEQTVVSGSMEVLRPCWAVATSGVPVREQLFNVSICHYTHFNLFQHGELYRSYSQGSDTASLEVATPILIPRSDGHRHRKGSHGDVIFLTNRGEITSYSPGLHGHDAIWQWQQSTGVTWSNLPSPSGMMEGGLVIPTLKPLSLRLHDNQEMILAAGEQEAVIISPGGSILATIELPGPPTHVLITEDFSNDGLTDLILVTSHGVYGFVQTRQPGALFFSMLVGCLIVVMGVIFVTQHLNSTKGKPRPSSGPR

**>GLYMA_01G170600**

MRKRDLAILMLSAFAIFFTLQQDGGISFKDAWMHLTDEYPIKYEAERLPPPLVADLNGDGKKEVLVATHDAKIQVLEPHSRRVDEGFSEARVLAEVSLLPDKVRVMTGRRPVAMATGYIDRYKIGQPQKQVLVVVTSGWSVMCFDSNLQKLWENNLQEDFPHNAHHREVAISISNYTLKHGDTGLIIVGGRMEMQPHIFMDPFEEMGMGARFAEQHRRSAAEKEASENSGTVDLRHFAFYAFAGRSGVERWSRKNENIEVHSSDASQLLPQHNYKLDVHALNTRQPGEGIQRINPGSYASPMGTFINLDYPSVLRLNLNYCPFLNKYVYQARREDTLLKLAHFRRHKRKTLKKTPGKAMSYPFHKPEENHPPGKDSTKKISNIIGKAANYAGSAKSKKHLPYVPTITNYTQVWWVPNVVVAHQKEGIEALHLASGRTICKLHLQEGGLHADINGDGVLDHVQAVGGNGAEQTVVSGSMEVLRPCWAIATSGVPIREQLFNVSICHYTHFNLFQHGELYRSYSQGSDIASLEVATPILIPRSDGHRHRKGSHGDVIFLTNRGEITSYSPGLHGHDAIWQWQQSTGVTWSNLPSPSGVMEGGGLVIPTLKPLSLRLHDNQEMILAAGEQEAVIISPGGSLLATIELPGPPTHVLIAEDFSNDGLTDLILVTSNGVYGFVQTRQPGALFFSMLVGCLIVVMGVIFVTQHLNSTKGKPRPSSGSR

**HUELLENLOS (HLL)**

**>AT1G17560 (AtHLL)**

MATALASKLSKGRSLLGGLCNAFSGLMNSSSNGMMNGSILSQQQHRTFIQMGTILKCVDNSCAKEVMCIQSLRGKKGARLGDIIVGSVKEANPIVQKKVKKDAIPKGKVKKGMVVYGVVVRAAMPKGRADGSQVKFDDNAIVVVGIKEKKGQNNSHGSKRKMEYNQPTGTRVFGPVPHEMRLRKQLKILSLAQHIV*

**>BnaC04g38260D**

MAAALASRLTKGRSLLGGLTNAFSGLMSSSNGSILSQQQQRTFIQMGTTLKVVDNSGGKEVMCIQSLRGKKGARLGDIVIGSVKEAAQKKDVKKGKDDIQKGKVKKGNVVYGVVVRAAMQKGRVDGSQVRFDDNAIVILGIKEKKKDENGKEKKKKHAGGFHQPLGTRVFGPVPHEMRLKKQLKILSLAQHLV

**>BnaA04g15320D**

MAAALASRLTKGRSLLGGLTNAFSGLMSSPSGSILSQQQQQRTFIQMGTTLKVVDNSGGKEVTCIQSLRGKKGARLGDIVIGSVKEAAHKGKVKKGNVVYGVVVRAAMQKGRVDGSQVRFDDNAIKRYAGGFHQPLGTRVFGPVPHEMRLKKQLKILSLAQHLV

**>BnaA06g36160D**

MAAALASRISRAGGRSLLGGLKNDFSGSIISSNGMMNESILLSQQQQQQRRTFIQMGTVLKVVDNSGAKKVMCIQALKGKKGARLGDTIVASVKEAMPNGKVKKGAVVYGVVVRAAMQRGRVDGSEVRFDDNAVVLVDNKDKKTKTDRQPIGTRVFGPVPHELRKKKHLKILALAQHIA

**>BnaA09g17870D**

MAAAFASRLSRAGRSLLGGLKNDLSGLMNTSYGMMNEASLSQQQQQRRTFIQMGTVLKVVDNSGAKKVMCIQALKGKKGARLGDTIVASVKEAMPNGKVKKGAVVYGVVVRAAMQRGRVDGSEVRFDDNAVVLVDNKDKKTKTDRQPIGTRVFGPVPHELRKKKHLKILALAQHIA

**>Csa_6G524020**

MAAAFASKFSRVSRSLLGGLGNNLSTLLTASKESICSSFISQLQQQRTFIQMRTVLKVVDNSGAKKVMCIQALKGKKGARLGDTIVASVKEAHPNGKVKKGKVVYGVVVRAAMQKGRCDGSEVKFDDNAVVLVDKQGQPIGTRVFGPVPHELRKKKHVKILTLAEHIA

**>Csa_2G401350**

MASAFASKFSRVGRSLLGGLGNNLSGSLTTSNETVCNSFITQQQRTFIQMRTVLKVVDNSGAKKVMCIQALKGKKGARLGDTIVASVKEAHPNGKVKKGKVVYGVVVRAAMQKDRCDGSEVKFDDNAVVLVDKQGQPIGTRVFGPVPHELRKKKHVKILTLAEHIA

**>GLYMA_08G310400**

MAAIFASRCSRVGRSLFGGLSNSSPGLFTTLHEMTCKNMFSQQQRTFIQMRTVLKVVDNSGAKKVMCIQALKGKKGARLGDTIIASVKEAHPNGKVKKGKVVYGVVVRAAMQKGRCDGSEVKFDDNAVVLVDKQGQPIGTRVFGPVPHELRQKKHVKILTLAGHIA

**>GLYMA_18G105500**

MAAAFASRCSRVGRSLSGGLSNSSPGLFTSSHGMTCNNLFSQQQRTFIQMRTVLKVVDNSGAKKVMCIQALKGKKGARLGDTIIASVKEAHPNGKVKKGKVVYGVVVRAAMQKGRCDGSEVKFDDNAVVLVDKQGQPIGTRVFGPVPHELTQKKHVKILTLAGHIA

**SHORT INTEGUMENTS 2 (SIN2)**

**>AT2G41670 (AtSIN2)**

MVMMLKKTVKKGLIGGMSFAKDAGKINWFPGHMAAATRAIRNRLKLSDLVIEVRDARIPLSSANEDLQSQMSAKRRIIALNKKDLANPNVLNKWTRHFESSKQDCIAINAHSRSSVMKLLDLVELKLKEVIAREPTLLVMVVGVPNVGKSALINSIHQIAAARFPVQERLKRATVGPLPGVTQDIAGFKIAHRPSIYVLDSPGVLVPSIPDIETGLKLALSGSVKDSVVGEERIAQYFLAILNIRGTPLHWKYLVEGINEGPHADCIDKPSYNLKDLRHQRTKQPDSSALHYVGDMISEVQRSLYITLSEFDGDTEDENDLECLIEQQFEVLQKALKIPHKASEARLMVSKKFLTLFRTGRLGPFILDDVPETETDHPNSKRVVVL*

**>BnaA05g02320D**

MVTGVKKVAKRGLIGGMSFAKEAGAINWFPGHMAAATRAIRSRLKLSDLVIEVRDARIPLSSANEDLQPQLSAKRRIIALNKKDLANPNVLNKWTHHFESKKQDCVAINAHSRSSVKKLLDLVEFKLKEVIAREPTLLVMVVGVPNVGKSALINSVHQIAATRFPVQDKLKRATVGPLPGVTQDIAGFKIAHRPSIYVLDSPGVLVPNIPDIETGLKLALSGSVKDSVVGEERLAQYFLAILNTRGTPLHWKYLFEGRNEGSAHPDSIDKPSYNLKDLRHQRSKQPDSSAVHYVGGMISEVQRSLYTTLSEFDGDTEDENDLECLIEQQFEALQKAFKVSTRASSEARLMVSKKFLTLFRTGRLGPFILDDVPET

**>Csa_4G168980**

MSKTPNLRRRRREQEMRGITGVVKKALGDMEFTAGGGAINWFPGHMAAATRAIRHRLKLADMVIEVRDSRIPLSSANQDLQPHLASKRRVIALNKKDLANPNIMNKWVNFFDSCNQDCVPINAHSKSSVRKLLELVEFKLKEAISREPTLLVMVVGVPNVGKSALINSIHQIASERFPVQEKRKRATVGPLPGVTQDIAGYKIAHQPSIYVLDTPGVLVPSIQDIETGLKLALAGSVKDAVVGEERIAQYLLAVLNSRRTPFHWRRHLNNRRVEGIRYEPEERHKFNLNDLQPKRRAPPNKSDVVYVEDLVTEVQCTLYTTLSEFDGNVEDENGLESLIEVQFEALQKAMKVSHKAAEARLRVSKKLLTLFRAGKLGQFILDDVPITKVS

**>GLYMA_03G135000**

MSGLKELLKKGLGLGDMAFNAGGGAITWFPGHMAAATRAIRHRLKLADLVIEVRDARIPLSSANADLQPHLSAKRRVVALNKKDLANPNIMHKWTHYFETCNQNCVAINAHSKSSVKKLLEVVEFKLKEVICKEPTLLVMVVGVPNVGKSALINSIHQIAKSRFPVQEKMKRAAVGPLPGVTQDIAGFKIAHKPSIYVLDTPGVLVPSISDIETGLKLALAGSVKDSVVGEERIVQYLLAVLNTRGTPLHWKHLNNRRIDGIEYEAEENHEYSLKNLKPKRRNLPNRSDLVYVEDLVMQVQRALYSSLSEFNGNVEDESDLESLIDLQFSALQKALKIPHKASEARLMVSKKFLTLFRTGKLGPFILDDVPDVKPVS

**>GLYMA_19G137600**

MSGLKELLKKGLGLGDMAFNAGGGAITWFPGHMAAATRAIRHRLKLADLVIEVRDARIPFSSANADLQPHLSAKRRVVALNKKDLANPNIMHKWTHYFESCNQNCVAINAHSMSSVKKLLEVVEFKLKEVICREPTLLVMVVGVPNVGKSALINSIHQIAKSRFPVQEKMKRAAVGPLPGVTQDIAGFKIAHKPSIYVLDTPGVLVPSISDIETGLKLALAESVKDSVVGEERIAQYLLAVLDTRGTPLHWNHLNNRRIDGIEYEAEENPEYSLKNLKPKRRNLPNRSDLVYVEDLVMGVQRALYSTLTEFDGNVEDESDLESLIDLQFSALQKALKIPHKASEARLMVSKKFLTLFRTGKLGPFILDDVPNVKPVS

**EPIDERMAL PATTERNING FACTOR‐LIKE 2 (EPFL2) and EPFL9**

**>AT4G37810 (AtEPFL2)**

MVWSSNMSSFLLILLILNSTHFSLMANGRPEPDSVEFTKSGDQDVKMMMRGLIGSRPPRCERVRCRSCGHCEAIQVPTNPQTKLHSPLTTSSSSSSETIHLDYTRGDDSTNYKPMSWKCKCGNSIYNP*

**>AT4G12970 (AtEPFL9)**

MKHEMMNIKPRCITIFFLLFALLLGNYVVQASRPRSIENTVSLLPQVHLLNSRRRHMIGSTAPTCTYNECRGCRYKCRAEQVPVEGNDPINSAYHYRCVCHR*

**>BnaC08g05340D**

MKHEMSNMKLRCISFFFLLFGLLLGNFIVEASKARSIDDTLSLPRQVHLPYSRRHMIGSTAPTCTYNECRGCRYKCRAEQVPVEGNDPINSAYHYRCVCHR

**>BnaA08g04900D**

MKHEMRNMKLRCISFFFLLFGLLLGNFIVEASKARSIDDTLSLPRQVHLPYSRRHMIGSTAPTCTYNECRGCRYKCRAEQVPVEGNDPINSAYHYRCVCHR

**>BnaC03g61250D**

MALSILCLKMALCSKMSSCLLMLLILNSTHFSLMANGRPEPDSREFIKRGDHDQKMVMRGLIGSSPPRCERVRCHSCGHCEAIQVPTNHQTKLHSPSSFSSSEIINLDYIRGEDTTNYKPMSWKCKCGNSIYNP

**>BnaAnng32490D**

MALSILCLKMALCSKMSSCLLMLLILNSTHFSLMANGRPEPNSREFIKRGDHDQKMVMRGLIGSNPPRCERVRCHSCGHCEAIQVPTNHQTKLHSPSSSFLSSEITNLDYIRGEDTTNYKPMSWKCKCGNSIYNP

**>BnaC01g01210D**

MALCRKMSSCLLILLILISTYFSLMANGRPEPSSYETTTGGDQDLKMLMRGLIGSSPPRCERVRCRACGHCEAIQVPTNPQTKLRHSPSSEIINLDYTRGDDSTNYKPMSWKCKCGNSIYNP

**>Csa_6G497000**

MGCECINNGVIGRRSRIILCPIVSLLFFLILASTQMRFMAEGRFISRNGKTVNNSEDKMVLRGQIGSRPPKCERRCSWCGHCEAIQVPANPQKSGTKNSSTMKNIAYARDEASNYKPMSWKCKCGSLIFNP

**>Csa_7G368090**

MAKAAKPLSVLLLLLLLLLLLTLFITSLFSTMAVQGFSTRDRPVPQFSSLQPTILPLEGRKERLRKWRRVMIGSTAPTCTYNECRGCKYKCRAEQVPVEGNDPINSAYHYRCVCHRITCYSISHQEVQTTSPTKLIK

**>GLYMA_01G196900**

MGRDHHLVVCGQRLSFLSISLCFLIISSWTQMGLVTEGRKTPKQNGFYQAVHDDKAMVRAQIGSRPPKCERRCRSCGHCEAIQVPTNPQAQNGKINSSTVSTIAFTMGEGGSNYKPMSWKCKCGNRIFNP

**>GLYMA_05G109900**

MGFDHYVICGQRLGFVGICLLFLIISSLIQKGLVIEGRKTQKLSHFHQTVNEDKIMLRPRIGSRPPKCERRCRSCEHCEAIQVPTNPQAQNRKKNSSKFSSIAYARVGGSSNYKPMSWKCKCGNLIFNP

**>GLYMA_17G157200**

MGFDHYVICVQRLGFVCICLLFLIISSWIQKGLVIEGRKTPKLSNFRQTVSEDKTMLRPRIGSRPPMCERRCRSCEHCEAIQVPTNPQMQNRKKNSSKFSSIAYARVGGSSNYKPMSWKCKCGNLIFNP

**>GLYMA_11G044700**

MSFSGRFPLTSGEIGGFLLFQCSTCLIYHLFVVLTSQVVNSNANASPLHCSNYYIIFGFLFLDRGSSTEGESLILSEMGRDHHHVVCGQRLSFLSISLCFLIISSWTQKGLVTEGRKTPKQNGFYQAVHDDKAMVRAQIGSRPPRCERRCRSCGHCEAIQVPTNPQAQNGKINSSTVSTIVFTMGEGSSNYKPMSWKCKCGNRIFNP

**>GLYMA_08G345100**

MLLCLTAHFGSLLSLMVINSPQHRYLSLSLSPSTLPLIFILSTNSYLSLKYIHHQHSYLQSCYCNTKVERMRDTKLPEVVFLLLFTLILASKFTQGIRTEESVSQSPQPQREPSLEDGNEAWKMRNSRRLMIGSTAPTCTYNECRGCKYKCRAEQVPVEGNDPINSPYHYRCVCHR

**>GLYMA_18G152800**

MLLCLTAHFGSLLSLMVINSPQHRYLSLLQPXSLPIFILFTNSYLSLKYIHHQHSYPPPPPPFKVFSAIYCNTKGERRMRDTKLPEVLLLLFTLILAAKFTQGIRTEELVSQSSHPQRESSLEDVNEAWKMRNSRRLMIGSTAPTCTYNECRGCKYKCRAEQVPVEGNDPINSPYHYRCVCHR

**ERECTA‐LIKE 1 (ERL1) and ERL2**

**>AT5G62230 (AtERL1)**

MKEKMQRMVLSLAMVGFMVFGVASAMNNEGKALMAIKGSFSNLVNMLLDWDDVHNSDLCSWRGVFCDNVSYSVVSLNLSSLNLGGEISPAIGDLRNLQSIDLQGNKLAGQIPDEIGNCASLVYLDLSENLLYGDIPFSISKLKQLETLNLKNNQLTGPVPATLTQIPNLKRLDLAGNHLTGEISRLLYWNEVLQYLGLRGNMLTGTLSSDMCQLTGLWYFDVRGNNLTGTIPESIGNCTSFQILDISYNQITGEIPYNIGFLQVATLSLQGNRLTGRIPEVIGLMQALAVLDLSDNELVGPIPPILGNLSFTGKLYLHGNMLTGPIPSELGNMSRLSYLQLNDNKLVGTIPPELGKLEQLFELNLANNRLVGPIPSNISSCAALNQFNVHGNLLSGSIPLAFRNLGSLTYLNLSSNNFKGKIPVELGHIINLDKLDLSGNNFSGSIPLTLGDLEHLLILNLSRNHLSGQLPAEFGNLRSIQMIDVSFNLLSGVIPTELGQLQNLNSLILNNNKLHGKIPDQLTNCFTLVNLNVSFNNLSGIVPPMKNFSRFAPASFVGNPYLCGNWVGSICGPLPKSRVFSRGALICIVLGVITLLCMIFLAVYKSMQQKKILQGSSKQAEGLTKLVILHMDMAIHTFDDIMRVTENLNEKFIIGYGASSTVYKCALKSSRPIAIKRLYNQYPHNLREFETELETIGSIRHRNIVSLHGYALSPTGNLLFYDYMENGSLWDLLHGSLKKVKLDWETRLKIAVGAAQGLAYLHHDCTPRIIHRDIKSSNILLDENFEAHLSDFGIAKSIPASKTHASTYVLGTIGYIDPEYARTSRINEKSDIYSFGIVLLELLTGKKAVDNEANLHQLILSKADDNTVMEAVDPEVTVTCMDLGHIRKTFQLALLCTKRNPLERPTMLEVSRVLLSLVPSLQVAKKLPSLDHSTKKLQQENEVRNPDAEASQWFVQFREVISKSSI*

**>AT5G07180 (AtERL2)**

MRRIETMKGLFFCLGMVVFMLLGSVSPMNNEGKALMAIKASFSNVANMLLDWDDVHNHDFCSWRGVFCDNVSLNVVSLNLSNLNLGGEISSALGDLMNLQSIDLQGNKLGGQIPDEIGNCVSLAYVDFSTNLLFGDIPFSISKLKQLEFLNLKNNQLTGPIPATLTQIPNLKTLDLARNQLTGEIPRLLYWNEVLQYLGLRGNMLTGTLSPDMCQLTGLWYFDVRGNNLTGTIPESIGNCTSFEILDVSYNQITGVIPYNIGFLQVATLSLQGNKLTGRIPEVIGLMQALAVLDLSDNELTGPIPPILGNLSFTGKLYLHGNKLTGQIPPELGNMSRLSYLQLNDNELVGKIPPELGKLEQLFELNLANNNLVGLIPSNISSCAALNQFNVHGNFLSGAVPLEFRNLGSLTYLNLSSNSFKGKIPAELGHIINLDTLDLSGNNFSGSIPLTLGDLEHLLILNLSRNHLNGTLPAEFGNLRSIQIIDVSFNFLAGVIPTELGQLQNINSLILNNNKIHGKIPDQLTNCFSLANLNISFNNLSGIIPPMKNFTRFSPASFFGNPFLCGNWVGSICGPSLPKSQVFTRVAVICMVLGFITLICMIFIAVYKSKQQKPVLKGSSKQPEGSTKLVILHMDMAIHTFDDIMRVTENLDEKYIIGYGASSTVYKCTSKTSRPIAIKRIYNQYPSNFREFETELETIGSIRHRNIVSLHGYALSPFGNLLFYDYMENGSLWDLLHGPGKKVKLDWETRLKIAVGAAQGLAYLHHDCTPRIIHRDIKSSNILLDGNFEARLSDFGIAKSIPATKTYASTYVLGTIGYIDPEYARTSRLNEKSDIYSFGIVLLELLTGKKAVDNEANLHQMILSKADDNTVMEAVDAEVSVTCMDSGHIKKTFQLALLCTKRNPLERPTMQEVSRVLLSLVPSPPPKKLPSPAKVQEGEERRESHSSDTTTPQWFVQFREDISKSSL*

**>BnaA06g21690D**

MKEMMQLMLVFMLLLLGVASPMNDEGKALMAIKGSFSNVVNMLLDWDDVHNSDFCSWRGVFCDNVSFSVVSLNLSNLNLGGEISPAVGDLRNLQSIDLQGNKLAGQIPDEIGNCASLVYLDFSDNLLYGDIPFSISKLKQLDTLNLKNNQLTGPLPATLTQIPNLKILDLAGNHITGEIPRLLYWNEVLQYFGLRGNMLTGTLSSDMCQFTGLWYFDVRGNNLTGTIPGSIGNCTSFEILDISYNQITGEIPYNIGFLQVATLSLQGNRLTGKIPEVIGLMQALAVLDLSDNELVGPIPPILGNLSFTGKLYLHGNKLTGSIPPELGNMSRLSYLQLNDNQLVGSIPPELGKLEQLFELNLANNRLVGPIPSNISSCAALNQFNVHGNLLNGSIPLAFRNLGSLTYLNLSSNNFKGKIPAELGHIINLDKLDLSGNSFSGSIPLTLGGLEHLLILNLSRNHLNGQLPAEFGNLRSIQMIDVSFNLLSGVIPTELGQLQNLNSLILNNNKLHGKIPDQLTNCFTLINLNVSFNNLTGIIPQMKNFSRFAPASFLGNPYLCGNWVGSICGPSLPKSRVFSKAVVICIVLGIITLLCMILIVVVKSKQQKEILKGSSSKQAEVSTKLVVLHMDMAIHTFDDIMRVTENFNKKFIIGYGASSTVYKCTLKTSRPIAIKRLYNQYQDNLREFETELETIGRIRHRNIVSLHGYALSSVGNLLFYDYMENGSLWDLLHVGTSKKVKLDWETRLKIAVGAAQGLAYLHHDCTPRIIHRDIKSSNILLDENFVAHLSDFGIAKSIPTSKTHASTYVLGTIGYIDPEYARTSRLNEKSDIYSFGIVLLELLTGKKAVDNESNLHQLILAKADDNTVMEAVDPEVTVTCMDLGHIRKTFKLALLCTKRNPLERPTMLEVSRVLLSLLPSMQVAKKLPSPDPSKKPTNYGVREQQQERSEEGSQWFEQFHEVISKSSV

**>BnaA10g23620D**

MEKVKSLGMVVVFFMLCGVVSPMNDEGKALMEMKASFSNVANMLLDWDDVHNSDFCSWRGVLCDNVSLSVVSLNLSNLNLGGEISPALGDLRSLQSIDLQGNNLGGQIPDELGNCASLAYLDISTNCLVGDIPFSISKLKQLEYLNLKNNQLTGPIPATLTQIPNLKTLDLAKNQLTGEIPRLLYWNEVLQYLGLRGNMLTGTLSPDMCQLTGLWYFDVRGNNLTGTIPDNIGNCTSYEILDISYNQITGVIPYNIGFLQVATLSLQENRLTGRIPEVIGLMQALAVLDLSGNELVGPIPPILGNLSFTGKLYLHGNKLTGPIPPELGNMSRLSYLQLNDNELVGTIPPELGKLEQLFELNLANNHLVGPIPANISSCAALNQFNVHGNLLNGSIPLGFRNLGSLTYLNLSANSFKGKIPSELGHIINLDTLDLSGNSFSGPIPLTLGDLEHLLILNLSRNYLNGPLPAEFGNLRSIQIMDVSFNSLSGVIPTELGLLQNIISLILNNNKIHGKIPDQLTNCFSLVNLNISFNNLSGIIPPMKNFSHFAPASFFGNPFLCGDWVGSICGPSLPKSQVITRTSVICMVLGFITLICMILIAVYKSKQQKNVLESSPKRSEGSTTKLVILHMDMAIHTFDDIMRLTENLSEKYAIGYGASSTVYKCTSSKTSRPIAIKRIYNHHPHNLREFETELETIGSIRHRNIVSLHGYALSPPLGNLLFYDYMENGSLWDLLHGPAGKKAKLDWETRLKIAVGAAQGLAYLHHDCTPRIIHRDVKSSNILLDGNFEARLSDFGIAKSIAAAKAYASTYVLGTIGYIDPEYARTSRLNEKSDVYSFGVVLLELLTGKKAVDNEANLHQLILSKADDNTVMDAVDAEVSVTCVDSGHIKKTFQLALMCAKRNPMERPTMQEVARVLLSLLPSPPPKKPPSPQGVEERRESHSLDTASPQWFVQFREAISKSSL

**>BnaC09g48340D**

MEKVKSLALFFMLCGVVSPMNDEGRALMEMKASFSNVANMLLDWDDVHNSDFCSWRGVLCDNVSLSVVSLNLSNLNLGGEISPALGDLRSLQSIDLQGNNLGGQIPDELGNCASLAYLDFSTNCLFGDIPFSISKLKQLEFLNLKNNQLTGPIPATLTQIPNLKTLDLAKNQLTGEIPRLLYWNEVLQYLGLRGNMLTGTLSPDMCQLTGLWYFDVRGNNLTGTIPDNIGNCTSYEILDVSYNQITGVIPYNIGFLQVATLSLQGNRLTGRIPEVIGLMQALAVLDLSDNELVGPIPPILGNLSFTGKLYLHGNKLTGPIPPELGNMSRLSYLQLNDNELVGTIPPELGKLEQLFELNLANNHLVGPIPANISSCAALNQFNVHGNFLNGSIPIGFRNLGSLTYLNLSSNSFKGKIPSELGHIINLDTLDLSGNSFSGPIPLTLGDLEHLLILNLSRNYLNGPLPAEFGNLRSIQIIDVSINSLTGVIPTELGLLQNINSLILNNNKIHGKIPDQLTNCFSLVNLNISFNNLSGIIPPMKNFSHFAPASFVGNPFLCGDWVGSICGPSLPKSQVITRTSVICTVLGFIALICMILIAVYKSKQQKHVLESSPKRSEGSTTKLVILHMDMAIHTFDDIMRLTENLSEKYAIGYGASSTVYKCTSSKTSRPIAIKRIYNHHPNNLREFETELETIGSLRHRNIVSLHGYALSPPLGNLLFYDFMENGSLWDLLHGPAGKKAKLDWETRLKIAVGAAHGLAYLHHDCTPRIIHRDVKSSNILLDGNFEARLSDFGIAKSIAAAKAYASSTYVLGTIGYIDPEYARTTRLNEKSDVYSFGVVLLELLTGKKAVDDEANLHQLILSKADDNTVMDAVDPEVSVTCVDSGHIKKTFQLALLCTKRNPMERPTMQEVARVLLSLLPSPPPKKPPSPAKLQGVEERRESQSSDTTSPQWFVQFREAISKSSL

**>Csa_5G014290**

MKLIPFLPWPNLHMVLLLLFFLSSPPLLYSSPVYSLPHNEGRALMSIKASFSNVANVLLDWDDDHNHDFCSWRGVFCDNVSLSVAALNLSNLNLGGEISPSIGDLRNLQSIDFQGNKLTGQIPDEIGNCGLLVHLDLSDNLLYGDIPFTVSKLKQLEFLNMKNNQLTGPIPSTLTQIPNLKTLDLARNQLTGEIPRLIYWNEVLQYLGLRGNFLTGSLSSDMCQLTGLWYFDVRGNNLTGSIPDSIGNCTSFEILDISYNQISGEIPYNIGFLQVATLSLQGNRLTGKIPDVIGLMQALAVLDLSENELDGPIPPILGNLSYTGKLYLHGNKLTGPIPPELGNMSKLSYLQLNDNQLVGTIPSELGKLDQLFELNLANNYLEGPIPHNISSCTALNQFNVHGNNLNGSIPLGFQNLESLTYLNLSANNFKGRIPVELGRIVNLDTLDLSCNHFLGPVPASIGDLEHLLSLNLSNNQLVGPLPAEFGNLRSVQMIDMSFNNLSGSIPMELGLLQNIISLILNNNHFQGKIPDRLTNCFSLANLNLSYNNLSGILPPMKNFSRFEPNSFIGNPLLCGNWLGSICGPYMEKSRAMLSRTVVVCMSFGFIILLSMVMIAVYKSKQLVKGSGKTGQGPPNLVVLHMDMAIHTFEDIMRSTENLSEKYIIGYGASSTVYKCLLKNSRPIAIKRLYNHYAHNFREFETELGTIGSIRHRNLVSLHGYSLSPCGNLLFYDYMENGSLWDLLHGTGKKVKLDWEARLKIAVGAAQGLAYLHHDCNPRIIHRDVKSSNILLDENFEAHLSDFGIAKCIPTAKTHASTYVLGTIGYIDPEYARTSRLNEKSDVYSFGIVLLELLTGKKAVDDESNLHQLILSKINSNTVMEAVDPEVSVTCIDLAHVRKTFQLALLCTKHNPSERPTMHEVSRVLISLQPPRPTVKQTSFPTKTLDYAQYVIEKGQNRNAKGGQEEQQKSDVNTSDARWFVQFGEVMSEQHSLNQ

**>GLYMA_20G151800**

MEDNAFLLLFYVGRRNWKLLMSPLLLVLLLLSPLASPFSEEGQALMAMKASFGNMADTLLDWDDAHNDDFCSWRGVFCDNVSLTVVSLNLSSLNLGGEISPAIGDLGNLQSIDLQGSKLTGQIPDEIGNCAALVHLDLSDNQLYGDIPFSLSKLKQLEFLNLKSNQLTGPIPSTLTQIPNLKTLDLARNRLTGEIPRILYWNEVLQYLGLRGNMLSGTLSPDICQLTNLWYFDVRGNNLTGTVPDSIGNCTSFEILDISYNRITGEIPYNIGFLQVATLSLQGNRLTGEIPEVIGLMQALAILDLSENELVGPIPPILGNLTFTGKLYLHGNMLTGSIPPELGNMSKLSYLQLNDNHLEGNIPNEFGKLEHLFELNLANNHLDGTIPHNISSCTALNQFNVHGNQLSGSIPLSFRSLESLTYLNLSANNFKGIIPVELGHIINLDTLDLSSNNFSGNVPASVGFLEHLLTLNLSHNHLDGPLPAEFGNLRSIQILDLSFNNLSGIIPPEIGQLQNLMSLIMNNNDLHGKIPDQLTNCFSLTSLNLSYNNLSGVIPSMKNFSRFSADSFLGNSLLCGDWLGSICCPYVPKSREIFSRVAVVCLTLGIMILLAMVIVAFYRSSQSKRLRKGSSRTGQGMLNGPPKLVILHMDMAIHTLDDIMRSTENLNEKYIIGYGASSTVYKCVLKNSRPIAIKRLYNQQAHNLREFETELETVGSIRHRNLVTLHGYALTPYGNLLFYDYMANGSLWDLLHGPLKVKLDWETRLRIAVGAAEGLAYLHHDCNPRIVHRDIKSSNILLDETFEAHLSDFGTAKCISTTRTHASTYVLGTIGYIDPEYARTSRLNEKSDVYSFGIVLLELLTGKKAVDNESNLHQLILSKADSNTVMETVDPEVSITCIDLAHVKKTFQLALLCTKKNPSERPTMHEVARVLVSLLPSPLSKILAPPAKKFDYAHFVIEKGQQRKVEEQKPQQDNILPNAQWFVRFGDVISKST

**>GLYMA_09G152400**

MEKAVLVLAFVGKLRRHFLLLVVAMVLVLLLSSFASPLSDEGQALMKIKASFSNVADVLHDWDDLHNDDFCSWRGVLCDNVSLTVFSLNLSSLNLGGEISPAIGDLVTLQSIDLQGNKLTGQIPDEIGNCAELIYLDLSDNQLYGDLPFSISKLKQLVFLNLKSNQLTGPIPSTLTQIPNLKTLDLARNRLTGEIPRLLYWNEVLQYLGLRGNMLSGTLSSDICQLTGLWYFDVRGNNLTGTIPDSIGNCTNFAILDLSYNQISGEIPYNIGFLQVATLSLQGNRLTGKIPEVFGLMQALAILDLSENELIGPIPPILGNLSYTGKLYLHGNMLTGTIPPELGNMSRLSYLQLNDNQVVGQIPDELGKLKHLFELNLANNHLEGSIPLNISSCTAMNKFNVHGNHLSGSIPLSFSSLGSLTYLNLSANNFKGSIPVDLGHIINLDTLDLSSNNFSGYVPGSVGYLEHLLTLNLSHNSLEGPLPAEFGNLRSIQIFDMAFNYLSGSIPPEIGQLQNLASLILNNNDLSGKIPDQLTNCLSLNFLNVSYNNLSGVIPLMKNFSWFSADSFMGNPLLCGNWLGSICDPYMPKSKVVFSRAAIVCLIVGTITLLAMVIIAIYRSSQSMQLIKGSSGTGQGPPKLVILHMGLAIHTFDDIMRVTENLNAKYIVGYGASGTVYKCALKNSRPIAIKRPYNQHPHNSREFETELETIGNIRHRNLVTLHGYALTPNGNLLFYDYMENGSLWDLLHGPLKKVKLDWEARLRIAMGAAEGLAYLHHDCNPRIIHRDIKSSNILLDENFEARLSDFGIAKCLSTTRTHVSTFVLGTIGYIDPEYARTSRLNEKSDVYSFGIVLLELLTGKKAVDNDSNLHHLILSKADNNTIMETVDPEVSITCMDLTHVKKTFQLALLCTKRNPSERPTMHEVARVLASLLPAPPSKNIFVPSSNTIDYAQFVIQKVNKNSLHTPQMDQWFVRFEDVVSNNSL

**>GLYMA_16G203300**

MEVALLLAFVGKLRRHFLLLVVGMVLVLFLSPFVSPLGDEGQALMKIKSSFSNVADVLHDWDALHNDDFCSWRGVLCDNVSLSVLFLNLSSLNLGGEISPAIGDLVNLQSIDLQGNKLTGQIPDEIGNCAELIYLDLSDNQLYGDIPFSISNLKQLVFLNLKSNQLTGPIPSTLTQISNLKTLDLARNRLTGEIPRLLYWNEVLQYLGLRGNMLSGTLSSDICQLTGLWYFDVRGNNLTGTIPDSIGNCTNFAILDLSYNQISGEIPYNIGFLQVATLSLQGNRLTGKIPEVIGLMQALAILDLSDNELIGPIPPILGNLSYTGKLYLHGNMLTGPIPPELGNMSRLSYLQLNDNQLVGQIPDELGKLEHLFELNLANNHLEGSIPLNISSCTALNKFNVHGNHLSGSIPLSFSRLESLTYLNLSANNFKGSIPVELGHIINLDTLDLSSNNFSGHVPGSVGYLEHLLTLNLSHNSLQGPLPAEFGNLRSIQIIDMSFNYLLGSVPPEIGQLQNLVSLILNNNDLRGKIPDQLTNCLSLNFLNVSYNNLSGVIPLMKNFSRFSADSFIGNPLLCGNWLGSICDLYMPKSRGVFSRAAIVCLIVGTITLLAMVTIAIYRSSQSTQLIKGSSGTGQGMLNIRTAYVYCLVLLWPPKLVILHMGLAIHTFDDIMRVTDNLNEKYIVGYGASSTVYKCVLKNSRPIAIKRLYNQHPHSSREFETELETIGSIRHRNLVTLHGYALTPNGNLLFYDYMENGSLWDLLHGPSKKVKLDWEARMRIAVGTAEGLAYLHHDCNPRIIHRDIKSSNILLDENFEARLSDFGIAKCLSTARTHASTFVLGTIGYIDPEYARTSRLNEKSDVYSFGIVLLELLTGKKAVDNDSNLHHLILSKADNNTIMETVDPEVSITCMDLTHVKKTFQLALLCTKKNPSERPTMHEVARVLASLLPAPPSKNIFVPSSKTIDYAQFVIQKGKQNNLHPLQMDRLQPQQFSNDQWFVRFEDVVSNNSL

**>GLYMA_10G242300**

MAMKALFSNMADVLLDWDDAHNDDFCSWRGVFCDNVSHTVVSLNLSSLNLGGEISPAIGDLTNLQSIDLQGNKLTGQIPDEIGNCAALVHLDLSDNQLYGDIPFSLSKLKQLELLNLKSNQLTGPIPSTLSQIPNLKTLDLARNRLSGEIPRILYWNEVLQYLDISYNQITGEIPFNIGFLQVATLSLQGNRLTGKIPEVIGLMQALAILDLSENELVGSIPPILGNLTFTGKLQLNDNGLVGNIPNEFGKLEHLFELNLANNHLDGTIPHNISSCTALNQLNLSSNNFKGIIPVELGHIINLDTLNLSHNHLDGSLPAEFGNLRSIEILDLSFNNISGSIPPEIGQLQNLMSLFMNHNDLRGKIPDQLTNCFSLTSLNLSYNNLSGVIPSMKNFSWFSADSFLGNSLLCGDWLGSKCRPYIPKSREIFSRVAVVCLILGIMILLAMVFVAFYRSSQSKQLMKGTSGTGQGMLNGPPKLVILHMDMAIHTLDDIIRGTENLSEKYIIGYGASSTVYKCVLKNSRPIAIKRLYNQQPHNIREFETELETVGSIRHRNLVTLHGYALTPYGNLLFYDYMANGSLWDLLHGPLKVKLDWETRLRIAVGAAEGLAYLHHDCNPRIVHRDIKSSNILLDENFEAHLSDFGTAKCISTAKTHASTYVLGTIGYIDPEYARTSRLNEKSDVYSFGIVLLELLTGKKAVDNESNLHQLILSKADNNTVMEAVDPEVSITCTDLAHVKKTFQLALLCTKKNPSERPSMHEVARVLVSLLPSPPSKILAPPAKKFDYAHFVIEKGQQRKMEEQKPQQDNNSSNAQWFVRFGDVISKST

**YUCCA 1 (YUC1)**

**>AT4G32540 (AtYUC1)**

MESHPHNKTDQTQHIILVHGPIIIGAGPSGLATSACLSSRGVPSLILERSDSIASLWKSKTYDRLRLHLPKHFCRLPLLDFPEYYPKYPSKNEFLAYLESYASHFRIAPRFNKNVQNAAYDSSSGFWRVKTHDNTEYLSKWLIVATGENADPYFPEIPGRKKFSGGKIVHASEYKSGEEFRRQKVLVVGCGNSGMEISLDLVRHNASPHLVVRNTVHVLPREILGVSTFGVGMTLLKCLPLRLVDKFLLLMANLSFGNTDRLGLRRPKTGPLELKNVTGKSPVLDVGAMSLIRSGMIQVSIMEGVKEITKKGAKFMDGQEKDFDSIIFATGYKSNVPTWLQGGDFFTDDGMPKTPFPNGWRGGKGLYTVGFTRRGLLGTASDAVKIAGEIGDQWRDEIKGSTRNMCSSRFVFTSKS*

**>BnaC03g67260D**

MDSHSCHQTKQIILVHGPIIIGAGPSGLATSACLSSRGVPSLILERSDSVASLWKSKTYDRLKLHLPKHYCRLPLLDFPENFPKYPSKNEFLDYLESYASHFGIVPRFNENVQNASYDSSSGLWRVKTLSGAEYLSKWLVVATGENADAYVPEVPGILKFSGGRIIHASEYKSGEEFRQQKVLVVGCGNSGMEISLDLVRHNASPHLVVRNTVHVLPREILGLSTFGIGMTLLKCLPLRFVDKFLLLMANLSFGNTDRLGLRRPKTGPLELKNVTGKTPVLDVGAMTLIRSGKIQIMEGVKEITKKGAKFMDGQEKEFDSIIFATGYKSNVPTWLQGSDFFTKEGMPKTPFPNSWRGGKGLYTVGFTRRGLLGTASDAVKIAGQIADQWRDIKGATKNLCSSRFVIISKS

**>BnaA08g12160D**

MDSHSCHQTKQIILVHGPIIIGAGPSGLATSACLSSRGVSSLILERSDSIASLWKSKTYDRLKLHLPKHYCRLPLLDFPENFPKYPSKNEFLDYLESYASHFGIVPRFNENVQNASYDSSSGLWRVKTLSGAEYLSKWLVVATGENADAYVPEVPGILKFSGGRIIHASEYKSGEEFRQQKVLVVGCGNSGMEISLDLVRHNASPHLVVRNTVHVLPREILGLSTFGIGMTLLKCLPLRFVDKFLLLMANLSFGNTDRLGLRRPKIGPLELKNLTGKTPVLDVGAMTLIRSGKIQIMEGVKEITKKGAKFMDGQEKEFDSIIFATGYKSNVPTWLQGSDFFTKEGMPKTPFPNSWRGGKGLYTVGFTRRGLLGTASDAVKIAGEIADQWRDIKGATKNLCSSRFVIISKS

**>BnaC01g05950D**

MDSPPSPPLPLHQTDHQTKQIILVHGSIIIGAGPSGLATSACLSNRGVPSLILERSDSIASLWKTKTYDRLKLHLPKHFCRLPLLDFPENFPKYPSKNEFLDYLESYASHFGIVPRFNENVVNAAFDTSSGLWRVKTLNKTEYLSKWLIVATGENADAYVPETPGIVKFSGGKIIHASDYRSGEEFRQQRVLVVGCGNSGMEISLDLVRHNASPHLVVRNTVHVLPREILGLSTFGVGMTLLKCLPLRLVDKFLLLMANLSFGNTDRLGIHRPKTGPLELKNVTGKSPVLDVGAMSLIRSGKIKIMEGVKEITKNGAKFMDGQEKEFDSIIFATGYKSNVPTWLQGSDFFTKEGMPKTPFPNGWRGGKGLYTVGFTRRGLLGTASDAVNIAGEIADQWRDEIKGPIKNMCSSRFVLISKS

**>BnaCnng74730D**

MDSHSCHQTKQIILVHGPIIIGAGPSGLATSACLSSRGVPSLILERSDSVASLWKSKTYDRLKLHLPKHYCRLPLLDFPENFPKYPSKNEFLDYLESYASHFGIVPRFNENVQNASYDSSSGLWRVKTLSGAEYLSKWLVVASGENADAYAPEVPGIVKFSGGRIIHASEYKSGEEFRQQKVLVVGCGNSGMEISLDLVRHNASPHLVVRNTVHVLPREILGLSTFGIGMTLLKCLPLRFVDKFLLLMANLSFGNTDRLGLRRPKTGPLELKNVTGKTPVLDVGAMTLIRSGKIQIMEGVQEITKKGAKFMDGQEKEFDSIIFATGYKSNVPTWLQGSDFFTKEGMPKTPFPNSWRGGKGLYT

**>Csa_2G379350**

MASCKDQEDDHHKQEPPPKCIWVHGPIIVGAGPSGLAAAACLSHNQTPSLILEKSDCIASLWQYRTYDRLKLHLPKQFCELPLMGFPENFPKYPSKDQFISYMESYASHFSIHPRFNQTVLAAEFDSVSGFWKVSTQDSQYISRWIIVATGENAEPVIPEIVGIERFARTVVHTSMYKSGSEFKNQRVLVVGCGNSGMEVSLDLCRQNAIPHMVVRNTVHVLPREMFGFSTFGIAMGLMKWLPLRLVDKILLLVANLTLGNTDHLGLRRPKTGPIELKNATGKTPVLDVGALSQIRSGKIKVMEGVKEITRNGAKFIDGQEKEFDSIILATGYRSNVPSWLKQGCDFFTKDGMPKTPFPNGWKGERGLYTVGFTRRGLLGTASDAMKIANDVAEQWRMAGNKDGKHCSTSYVIVLKESIRK

**>GLYMA_04G070100**

MESLKGEEVKCVWVQGPIIVGAGPSGLAVAACLSHHGVPYVILERSHCITSLWQHRTYDRLKLHLPKHFCELPLMPFPLHFPKYPSKNQFISYLNSYASRFNIRPRFNQSVQTAEFDPSSQLWLVRTNGFQYISPWLVVATGENAEPVVPSISGMDMFHGPIVHTSVYKSGSDYNNQRVLVIGCGNSGMEVSLDLCRHNANPYMVARNTVHVLPREMFGFSTFGIAMALLKWLPIKVVDKLVLAAARLMLGDTARYGVRRPKTGPIELKLVTGKTPVLDVGQVAQIRSGNIKVMEGVKEITRNGAKFMDGQEKEFSAIILATGYKSNVPTWLKSCESFTKDGMPKTPFPMGWKGENGLYTVGFTRRGLLGTASDAVKIAKDIADQWMTVKDKSYRNSHIILLKST

**>GLYMA_14G128200**

MGRSCNKSQQQHVEGPIIIGAGPSGLAVAACLSEDKVPFVILERHNCIASLWQNKTYDRLKLHLPKQFCELPLKGFPHTFPKYPTKYQFISYMESYASHFNIHPIFNQTVKSAEFDKGSNVWVVRTEEFEYSSRWLVVATGENAEPVVPRIHGMELFGGAVAHTSVYKSGSEYRNKKVLVIGCGNSGMEVCLDLCRHNAKPYMVARNTVHVLPREMLGFSTFGIAMALYKWFPIKLVDKIILLATNLILGNTNHYGIKRPKTGPIELKLATGKTPVLDVGQVAQIKCGNIKVMEGVKEITRNGAKFMDGKEKEFDAIILATGYKSNVPTWLKGCDFFTKDGMPKTPFPHGWKGEQGMYTVGFTRRGLHGTSCDAIKIAEDIAEQWRTVEDKSHCDSHIILLNNS

**>GLYMA_17G205800**

MGSCNKPQQQQQQHVQGPIIIGAGPSGLAVAACLSEHKVPFVILERHNCIASLWQNKTYDRLKLHLPKQFCELPLKGFPHTFPKYPTKYQFISYMESYASHFNIHPIFNQTVESADFDKGSKVWVVKTQEVDYSSRWLVVATGENAEPVVPRIHGMELFNGDVAHTSVYKSGSEYRNKKVLVIGCGNSGMEVCLDLCRHNAKPYMVARNTVHVLPREMFGFSTFGVAMALYKWFPIKLVDKIILLATNFILGNTNHYGIKRPKTGPIELKLATGKTPVLDVGQVAQIKCGNIKVMEGVKEITRNGAKFMDGQEKEFDAIILATGYKSNVPTWLKGCDFFTEDGMPKTPFPHGWKGEQGLYTVGFTRRGLQGTSCDAIKIAEDIAEQWRTVEDKSHCDSHIILLNNS

**>GLYMA_06G072100**

MESLKAQEGGEEQVKCVWVHGPIIVGAGPSGLAVAACLSHHGVPYVILERTNCITSLWQHRTYDRLKLHLPKHFCELPLIPFPLHFPKYPSNSYASRFNIRPRFNQSVQTAQFDPCSQLWVVKTNGFQYISPWLVVATGENAEPVVPSISGMDKFRGPIVHTSVYKSGSDYKNQRVLVIGCGNSGMEVSLDLCRHNANPYMVARNTVHVLPMEMFGFSTFGIAMALLKWLPIKLVDKLVLAAARLMLGDTARYGVRRPKTGPIELKLVTGKTPVLDVGQVAQIRSGNIKVMEGVKEITRNGAKFMDGQEKEFSAIILATGYKSNVPTWLKSCESFTKDGMPKTPFPMGWKGENGLYTVGFTRRGLLGTASDAVKIAKDIADQWMTVKDRSYCNSHIIFLKST

**PIN-FORMED 1 (PIN1) and PIN3**

**>AT1G73590 (AtPIN1)**

MITAADFYHVMTAMVPLYVAMILAYGSVKWWKIFTPDQCSGINRFVALFAVPLLSFHFIAANNPYAMNLRFLAADSLQKVIVLSLLFLWCKLSRNGSLDWTITLFSLSTLPNTLVMGIPLLKGMYGNFSGDLMVQIVVLQCIIWYTLMLFLFEYRGAKLLISEQFPDTAGSIVSIHVDSDIMSLDGRQPLETEAEIKEDGKLHVTVRRSNASRSDIYSRRSQGLSATPRPSNLTNAEIYSLQSSRNPTPRGSSFNHTDFYSMMASGGGRNSNFGPGEAVFGSKGPTPRPSNYEEDGGPAKPTAAGTAAGAGRFHYQSGGSGGGGGAHYPAPNPGMFSPNTGGGGGTAAKGNAPVVGGKRQDGNGRDLHMFVWSSSASPVSDVFGGGGGNHHADYSTATNDHQKDVKISVPQGNSNDNQYVEREEFSFGNKDDDSKVLATDGGNNISNKTTQAKVMPPTSVMTRLILIMVWRKLIRNPNSYSSLFGITWSLISFKWNIEMPALIAKSISILSDAGLGMAMFSLGLFMALNPRIIACGNRRAAFAAAMRFVVGPAVMLVASYAVGLRGVLLHVAIIQAALPQGIVPFVFAKEYNVHPDILSTAVIFGMLIALPITLLYYILLGL*

**>AT1G70940 (AtPIN3)**

MISWHDLYTVLTAVIPLYVAMILAYGSVRWWKIFSPDQCSGINRFVAIFAVPLLSFHFISTNNPYAMNLRFIAADTLQKIIMLSLLVLWANFTRSGSLEWSITIFSLSTLPNTLVMGIPLLIAMYGEYSGSLMVQIVVLQCIIWYTLLLFLFEFRGAKMLIMEQFPETAASIVSFKVESDVVSLDGHDFLETDAEIGDDGKLHVTVRKSNASRRSFCGPNMTPRPSNLTGAEIYSLSTTPRGSNFNHSDFYNMMGFPGGRLSNFGPADMYSVQSSRGPTPRPSNFEENCAMASSPRFGYYPGGGAGSYPAPNPEFSSTTTSTANKSVNKNPKDVNTNQQTTLPTGGKSNSHDAKELHMFVWSSNGSPVSDRAGLNVFGGAPDNDQGGRSDQGAKEIRMLVPDQSHNGETKAVAHPASGDFGGEQQFSFAGKEEEAERPKDAENGLNKLAPNSTAALQSKTGLGGAEASQRKNMPPASVMTRLILIMVWRKLIRNPNTYSSLIGLIWALVAFRWHVAMPKIIQQSISILSDAGLGMAMFSLGLFMALQPKLIACGNSVATFAMAVRFLTGPAVMAVAAIAIGLRGDLLRVAIVQAALPQGIVPFVFAKEYNVHPAILSTGVIFGMLIALPITLVYYILLGL*

**>BnaA02g16500D**

MITATDFYHVMTAMVPLYVAMILAYGSVKWWKIFTPTQCSGINRFVALFAVPLLSFHFIAANNPYAMNLRFLAADSLQKVIVLSLLFLWCKLSPNGSLDWTITLFSLTTLPNTLVMGIPLLKGMYGDFSGDLMVQIVVLQCIIWYTLMLFLFEYRGAKLLISEQFPDTAGSIVSIHVDSDIMSLDGRQPLETEAEIKEDGKLHVTVRRSNASRSDIYSRRSQGLSATPRPSNLTNAEIYSLQSSRNPTPRGSSFNHTDFYSMMAAGGGRNSNFGPGEAVFGSKGPTPRPSNYEEDGGAKPAAGAGRFPYHGGGGAAAKGNDGNGRDLHMFVWSSSASPVSDVFGGGVNHHSDYAAATNEQHKDGNSNDSQYVEREDFSFGNKDDDSKVLATDNNISNKAQQQQPKVMPPTSVMTRLILIMVWRKLIRNPNSYSSLFGITWSLISFKWNIEMPAIIAKSISILSDAGLGMAMFSLGLFMALNPRIIACGNRRAAFAAGMRFLAGPAVMTVASYAVGLRGVLLRVAIIQAALPQGIVPFVFAKEYNVHPDILSTAVIFGMLIALPITLLYYILVGL

**>BnaC02g22310D**

MITATDFYHVMTAMVPLYVAMILAYGSVKWWKIFTPTQCSGINRFVALFAVPLLSFHFIAANNPYAMNLRFLAADSLQKVIVLSLLFLWCKLSPNGSLDWTITLFSLTTLPNTLVMGIPLLKGMYGDFSGDLMVQIVVLQCIIWYTLMLFLFEYRGAKLLISEQFPDTAGSIVSIHVDSDIMSLDGRQPLETEAEIKEDGKLHVTVRRSNASRSDIYSRRSQGLSATPRPSNLTNAEIYSLQSSRNPTPRGSSFNHTDFYSMMAAGTAAKGNDGNGRDLHMFVWSSSASPVSDVFGGGGNHHADYAAATNDHHKDGNSNDNQYVEREEFSFGNKDDDSKVLAADNNISNKAQQQQPKVMPPTSVMTRLILIMVWRKLIRNPNSYSSLFGITWSLISFKWNIEMPAIIAKSISILSDAGLGMAMFSLGLFMALNPRIIACGNRRAAFAAGMRFLAGPAVMTVASYAVGLRGVLLRVAIIQAALPQGIVPFVFAKEYNVHPDILSTAVIFGMLIALPITLLYYILLGL

**>BnaA07g28990D**

MISWHDLYTVLTAVIPLYVAMILAYGSVRWWKIFSPDQCSGINRFVAIFAVPLLSFHFISTNNPYAMNLRFIAADTLQKILMLALLVLWANFTRSGSLEWSITIFSLSTLPNTLVMGIPLLIAMYGEYAGSLMVQVVVLQCIIWYTLLLFLFEFRGAKMLIMEQFPETAASIVSFKVESDVVSLDGHDFLETDAEIGNDGKLHVTVRKSNASRRSFCGPNMTPRPSNLTGAEIYSLSTTPRGSNFNNSDFYSMMGFPGGRLSNFGPADMYSVQSSRGPTPRPSNFEENSAIASSPRFGYYPGGGGGAKELHMFVWSSNGSPVSDRAGLNVSDGAPEIRMLVPDQSQNGESKALARPASGDFGGERFSFAGREEEGERAKDAENGLNKHAPSSSAELQLKTGLGAAGGGEGSHVKHMPPASVMTRLILIMVWRKLIRNPNTYSSLIGLIWALVAFRWHVAMPKIIQQSISILSDAGLGMAMFSLGLFMALQPKLIACGNSVATFAMAVRFLTGPAVMAVASIAIGLHGDLLRVAIVQAALPQGIVPFVFAKEYNVHPAILSTGVIFGMLIALPITLVYYILLGL

**>BnaC06g24400D**

MISWHDLYTVLTAVIPLYVAMILAYGSVRWWKIFSPDQCSGINRFVAIFAVPLLSFHFISTNNPYAMNLLVVLQCIIWYTLLLFLFEFRGAKMLIMEQFPETAASIVSFKVESDVVSLDGHDFLETDAEIGNDGKLHVTVRKSNASRRSFCGPNMTPRPSNLTGAEIYSLSTTPRGSNFNQSDFYSMMGFPGGRPTPRPSNFEENCAMASSPKFGSKELHMFVWSSNGSPVSDRAGLNAPDLGSNNEQEIRMLVPDQSQNSENKALARPASGDFGGEPVIITRREEGERAKDAENGLNKAVPNAVLQSKTGLGAAAEGEASQGKHMPPASVMTRLILIMVWRKLIRNPNTYSSLIGLIWALVAFRWHVAMPKIIQQSISILSDAGLGMAMFSLGLFMALQPKLIACGNSVATFAMAVRFLTGPAVMAVASIAIGLRGDLLRVAIVQAALPQGIVPFVFAKEYNVHPAILSTGVIFGMLIALPITLVYYILLGL

**>Csa_5G576590**

MISWKDLYTVLTAVIPLYVAMILAYGSVRWWKIFTPDQCSGINRFVAIFAVPLLSFHFISSNDPYAVNFRFIAADTLQKIIMLFFLGIWTNFTKNGSLEWMITIFSLSTLPNTLVMGIPLLIAMYGEYSGSLMVQVVVLQCIIWYTLLLFLFEYRGAKILIMEQFPETAASIVSFKVDSDVVSLDGRDFLETDAEIGDDGKLHVTVRKSNASRRSLGPCSLPALTPRPSNLTGAEIYSLSSSRNPTPRGSNFNNSDFYSMMGFQGRLSNFGPGDLYSVQSSRGPTPRPSNFEENSAVQPQTASPRFGFYPAQTVPSSYPAPNPEFTKTAKIPQPPPPPPPQQPQQQPQNAKPNHDAKELHMFVWSSSASPVSEGAGGLHIFAGNEVAGAEQSGRSDQGAKEIRMLVADHPQNGENKENEGYVGEAFSFSGKEGEDERDDQKEGPTGSTGDQLHGKVSAGAPDGVNSKLMPPASVMTRLILIMVWRKLIRNPNTYSSLIGLIWSLISFRWHVAMPKIIERSISILSDAGLGMAMFTLGIFMGLQPKMIACGNSVATFAMAIRLLTGPAVMAIASIAIGLRGTLLRVAIVQAALPQGIVPFVFAKEYNVHPAILSTWVIFGMLIALPITLLYYVLLGL

**>Csa_5G284520**

MITGKDLYTVFTAVIPLYVAMILAYGSVRWWRIFSPDQCSGINRFVAIFAVPLLSFHFISTNDPYAMNFRFIAADTLQKIIMLVALTIWANFTKNGSLESMITIFSLSTLPNTLVMGIPLLQAMYGGNSGSLMVQVVVMQCIIWYTLLLFLFEYRGAKILIMEQFPETAASIVSFKVDSDVVSLDGRDFLETDAEIGNDGKLHVTVRKSNASRRSLGPCSLPALTPRPSNLTGAEIYSLSSSRNPTPRGSNFNHTDFYSLMGYQGRHSNNGQPELYPVQSSRGPTPRPSNIEESTGIPTANSPRFGFYPAPNPEFTKSGRTQPPQQQQPPLQNGGPTSKASHDAKELHMFVWSSSASPVSETDGLHVFNGTDFGASEQIGRSSDQNAKEIRMFIADHPQNGERKVGESEGKFRGEELNFQDVDNEKEEQVPIGLQTLAPVPPPHAAATATAAAPESGAAKQMPPTSVMTRLILIMVWRKLIRNPNTYSSLIGLAWSLIAFRWHVSMPKIIAQSISILSDAGLGMAMFSLGIFMALQPKLIACGNSIAAFAMAVRFLTGPAVMAAASVAIGLHGNLLRVAIVQAALPQGIVPFVFAKEYNVHPAILNTAVIFGMLIALPITLVYYILLGL

**>Csa_1G042820**

MITLSDFYHVMTAVVPLYVAMILAYGSVKWWKIFTPDQCSGINRFVALFAVPLLSFHFISTNNPYTMNLRFIAADTLQKLIVLAVLAVWSNISKRGCLEWTITLFSLSTLPNTLVMGIPLLKGMYGDFSGSLMVQIVVLQCIIWYTLMLFMFEYRGARMLISEQFPDTAGSIVSIHVDSDIMSLDGRQVLETEAEIKEDGKLHVTVRKSNASRSDIFSRRSVGLSSTTPRPSNLTNAEIYSLQSSRNPTPRGSSFNHTDFYSMMAAGGRNSNFGSSDVYGLSASRGPTPRPSNYEEEGGGNGGKPRFHYNATTGGNANANANANVNHYPAPNPGMFSPTGSKNAQPNNAKKPANGKTEDGSRDLHMFVWSSSASPVSDVFGNHEFGAHNDQKDVRLAVSPGKEGRRENQEEYAEREDFSFGNREMMNSNNNGGVGVGGTEKVGDIKPKTMPPTSVMTRLILIMVWRKLIRNPNTYSSLIGLTWSLVSFRWNVEMPAIIAKSISILSDAGLGMAMFSLGLFMALQPRIIACGNSIAAFSMAVRFLTGPAVMAVASIAVGLRGVLLRVAIVQAALPQGIVPFVFAKEYNVHPDILSTGVIFGMLVALPITLVYYILLGI

**>GLYMA_07G102500**

MITLTDFYHVMTAMVPLYVAMILAYGSVKWWKIFSPDQCSGINRFVALFAVPLLSFHFIASNNPYEMNLRFLAADTLQKIIILVLLAVWSNIAKRGCLEWAITLFSLSTLPNTLVMGIPLLKGMYGDFSGSLMVQIVVLQCIIWYTLMLFLFEFRGARMLISEQFPDTAGSIVSIHVDSDVMSLDGRQPLETEAEIKEDGKLHVTVRKSNASRSDIFSRRSQGLSSTTPRPSNLTNAEIYSLQSSRNPTPRGSSFNHTDFYSMMAAGGRNSNFGASDVYGLSASRGPTPRPSNYDEDGGKPKFHYHAGGTGHYPAPNPGMFSPSNGSKSVAAANANANAKRPNGQAQLKPEDGNRDLHMFVWSSSASPVSDVFGAHEYGGHDQKEVKLNVSPGKVENHRDTQEDYLEKDEFSFGNRGMDREMNQLEGEKVGDGKPKTMPPASVMTRLILIMVWRKLIRNPNTYSSLIGLTWSLVSFKWNVEMPAIIAKSISILSDAGLGMAMFSLGLFMALQPRVIACGNSTAAFAMAVRFLTGPAVMAAASVAVGLKGVLLHVAIVQAALPQGIVPFVFAKEYNVHPDILSTAVIFGMLIALPITLVYYILLGL

**>GLYMA_08G054700**

MITLTDFYHVMTAMVPLYVAMILAYGSVKWWKIFSPDQCSGINRFVALFAVPLLSFHFIASNNPYEMNFRFIAADTLQKMIVLVVLGIWANVSKRGCLEWTITLFSISTLPNTLVMGIPLLKGMYGEFSGSLMVQIVVLQCIIWYTLMLFMFEYRGARLLISEQFPDTAGTIVSIHVDSDVMSLDGRQHPLETDAQIKEDGKLHVTVRKSNASRSDIFSRRSQGFSSTTPRPSNLTNAEIYSLQSSRNPTPRGSSFNHTDFYSMMAAGRNSNFGANDVYGLSASRGPTPRPSNYDEDASNNNNGKPRYHYPAAGTGTGTGTGTGTGTGHYPAPNPGMFSPTASKNVAKKPDDPNKDLHMFVWSSSASPVSDVFGGGHEYDHKELKLTVSPGKVEGNINRDTQEEYQPEKDEFSFGNRGIEDEHEGEKVGNGNPKTMPPASVMTRLILIMVWRKLIRNPNTYSSLIGLTWSLISFRWNVKMPAIIAKSISILSDAGLGMAMFSLGLFMALQPRIIACGNSTAAFSMAVRFLTGPAVMAAASIAVGLKGVLLHVAIVQAALPQGIVPFVFAKEYNVHPDILSTGVIFGMLIALPITLVYYILLGL

**>GLYMA_09G176300**

MVPLYVAMILAYGSVKWWKIFSPDQCSGINRFVALFAVPLLSFHFIASNNPYEMNLRFLAADTLQKIIILVLLAVWSNITKRGCLEWAITLFSLSTLPNTLVMGIPLLKGMYGDFSGSLMVQIVVLQCIIWYTLMLFLFEFRGARMLISEQFPDTAASIVSIHVDSDVMSLDGRQPLETEAEIKEDGKLHVTVRKSNASRSDIFSRRSQGLSSTTPRPSNLTNAEIYSLQSSRNPTPRGSSFNHTDFYSMMAAGGRNSNFGASDVYGLSASRGPTPRPSNYDEDGGKPKFHYHAAGGTGHYPAPNPGMFSPSNGSKSVAANANAKRPNGQAQLKPEDGNRDLHMFVWSSSASPVSDVFGAHEYGGGHDQKEVKLNVSPGKGFIFVFVLSRLDFLRNLFRMENNHRDTQEDYLEKDEFSFGNREMDREMNQLEGLFMALQPRVIACGNSTAAFAMAVRFLTGPAVMAAASIAVGLKGVLLHVAIVQTRL

**>GLYMA_20G014300**

MITWGDFYTVMSAVIPLYVAMILAYGSVRWWKIFSPDQCSGINRFVAIFAVPLLSFHFISLNNPYAMNFRFIAADTLQKIIMLFALAIWTNFSANGSLEWMITIFSLSTLPNTLVMGIPLLIAMYGEYSGLLMVQVVVLQCIIWYTLLLFLFEYRGAKLLIMEQFPETAASIVSFKVDSDVVSLDGRDFLETDAEVGDDGKLHVTVRKSNASRRSFMMTPRPSNLTGAEIYSLSSSRNPTPRGSNFNHADFYSMMGYAPRHSNFGAADLYSVQSTSRGVTPRPSNFEENGAPAAATTQQQQQAISSPRFGFYPAVQTVPAAYPAPNPEFSSSGLTKSVSKNSQTQPQPQPQPQIQAQVAPPPQPQVAQPTNSGNKANHDAKELHMFVWSSSTSPVSEAGGLHVFSGADFGASDQSGRSEQGAKEIRMLVADDHPQNGETNKAAAEGEFGGEELKFPGKEGEQADEEGEKAGPGGLNKLGSSSTAELHPKSAVAVAGKHMPPASVMTRLILIMVWRKLIRNPNTYSSLIGVIWSLVAFRWHVQMPKIIEKSISILSDAGLGMAMFSLGLFMALQPKIIACGNSVATFAMAVRFLTGPAVMAAASIAVGLRGTLLRVAIVQAALPQGIVPFVFAKEYNVHPAILSTAVIFGMLIALPITLLYYILLGL

**>GLYMA_09G117900**

MITWKDLYMVLTAVVPLYVAMILAYGSVRWWKIFSPDQCSGINRFVAIFAVPLLSFHFISTNNPYAMNFRFIAADTLQKIIMLFALAIWTNLTKTGSLEWMITIFSLSTLPNTLVMGIPLLIAMYGEYSGSLMVQVVVLQCIIWYTLLLFLFEYRAAKILIMEQFPETAASIVSFKVDSDVVSLDGRDFLETDAEVGDDGKLHVTVRKSNASRRSFMMTPRPSNLTGAEIYSLSSSRNPTPRGSNFNHADFFSMMGYQPRHSNFTANDLFSSRGPTPRPSNFEESSMPQAATVASPRFGFYPSQTVPASYPPPNPEFSSSTKHLKSQSQNSLTPANGAHDAKELHMFVWSSSASPVSENAGLNVFGNTELGTSQQPDQGGAKEIRMLVADNHAHLQNGEANNKGGLEAGLDVEEFKFPVNGGEQVEEEKEKEKEGLNNGLNKLGSSSTAELHPKAAGAAEAPASKHMPPASVMTRLILIMVWRKLIRNPNTYSSLIGVVWSLIAFRWHVHMPKIIEKSISILSDAGLGMAMFSLGLFMALQPKIIACGNSVATFAMAIRFLTGPAVMAAASIAVGLRGTLLHVAIVQAALPQGIVPFVFAKEYNAHPAILSTAVIFGMLIALPITLVYYILLGL

**>GLYMA_07G164600**

MITWKDLYTVLTAVVPLYVAMILAYGSVRWWKIFSPDQCSGINRFVAIFAVPLLSFHFISTNNPYAMNFRFIAADTLQKIIMLFALAIWTNLTKTGSLEWMITIFSLSTLPNTLVMGIPLLIAMYGDYSGSLMVQVVVLQCIIWYTLLLFLFEYRAAKILIMEQFPETAASIVSFKVDSDVVSLDGRDFLETDAEVVTMGSFMLLISTGPEINSLSSSRNPTPRGSNPRHSNFTANDLFSSRGPTPRPSNFEEPSMPQAVTVASPRFGFYPSQTVPASYPPPNPDFSSATKNLKNQSQNQNPNQSQSQNSQAPAKGAHDAKELHMFVWSSSASPMSENAGLNVFSSTDLGTSEQPDQGAKEIRMLVADNNAHLRNGEANNKATVAGEASAGKHMPPANVMTRLILIMVWRKLIRNPNTYSSLIGVVWSLVAFRWHVHMPKIIEKSISILSDAGLGMAMFSLGLFMALQPKIIACGNSVATFAMAIRFLTGPAVMAAASIAVGLRGTLLHVAIVQAALPQGIVPFVFAKEYNVHPAILSTAVIFGMLIALPITLVYYILLGL

**AUXIN RESPONSE FACTOR 3 / ETTIN (ARF3/ETT) and AUXIN RESPONSE FACTOR 5 / MONOPTERROS (ARF5/MP)**

**>AT2G33860 (AtARF3)**

MGGLIDLNVMETEEDETQTQTPSSASGSVSPTSSSSASVSVVSSNSAGGGVCLELWHACAGPLISLPKRGSLVLYFPQGHLEQAPDFSAAIYGLPPHVFCRILDVKLHAETTTDEVYAQVSLLPESEDIERKVREGIIDVDGGEEDYEVLKRSNTPHMFCKTLTASDTSTHGGFSVPRRAAEDCFPPLDYSQPRPSQELLARDLHGLEWRFRHIYRGQPRRHLLTTGWSAFVNKKKLVSGDAVLFLRGDDGKLRLGVRRASQIEGTAALSAQYNQNMNHNNFSEVAHAISTHSVFSISYNPKASWSNFIIPAPKFLKVVDYPFCIGMRFKARVESEDASERRSPGIISGISDLDPIRWPGSKWRCLLVRWDDIVANGHQQRVSPWEIEPSGSISNSGSFVTTGPKRSRIGFSSGKPDIPVSEGIRATDFEESLRFQRVLQGQEIFPGFINTCSDGGAGARRGRFKGTEFGDSYGFHKVLQGQETVPAYSITDHRQQHGLSQRNIWCGPFQNFSTRILPPSVSSSPSSVLLTNSNSPNGRLEDHHGGSGRCRLFGFPLTDETTAVASATAVPCVEGNSMKGASAVQSNHHHSQGRDIYAMRDMLLDIAL*

**>AT1G19850 (AtARF5)**

MMASLSCVEDKMKTSCLVNGGGTITTTTSQSTLLEEMKLLKDQSGTRKPVINSELWHACAGPLVCLPQVGSLVYYFSQGHSEQVAVSTRRSATTQVPNYPNLPSQLMCQVHNVTLHADKDSDEIYAQMSLQPVHSERDVFPVPDFGMLRGSKHPTEFFCKTLTASDTSTHGGFSVPRRAAEKLFPPLDYSAQPPTQELVVRDLHENTWTFRHIYRGQPKRHLLTTGWSLFVGSKRLRAGDSVLFIRDEKSQLMVGVRRANRQQTALPSSVLSADSMHIGVLAAAAHATANRTPFLIFYNPRACPAEFVIPLAKYRKAICGSQLSVGMRFGMMFETEDSGKRRYMGTIVGISDLDPLRWPGSKWRNLQVEWDEPGCNDKPTRVSPWDIETPESLFIFPSLTSGLKRQLHPSYFAGETEWGSLIKRPLIRVPDSANGIMPYASFPSMASEQLMKMMMRPHNNQNVPSFMSEMQQNIVMGNGGLLGDMKMQQPLMMNQKSEMVQPQNKLTVNPSASNTSGQEQNLSQSMSAPAKPENSTLSGCSSGRVQHGLEQSMEQASQVTTSTVCNEEKVNQLLQKPGASSPVQADQCLDITHQIYQPQSDPINGFSFLETDELTSQVSSFQSLAGSYKQPFILSSQDSSAVVLPDSTNSPLFHDVWDTQLNGLKFDQFSPLMQQDLYASQNICMSNSTTSNILDPPLSNTVLDDFCAIKDTDFQNHPSGCLVGNNNTSFAQDVQSQITSASFADSQAFSRQDFPDNSGGTGTSSSNVDFDDCSLRQNSKGSSWQKIATPRVRTYTKVQKTGSVGRSIDVTSFKDYEELKSAIECMFGLEGLLTHPQSSGWKLVYVDYESDVLLVGDDPWEEFVGCVRCIRILSPTEVQQMSEEGMKLLNSAGINDLKTSVS*

**>BnaA06g14040D**

MMASVEEKMKTNGLVNGGTTTTSQSSLLEEMKLLKDQSGTRKPVINSELWHACAGPLVCLPQVGSLVYYFSQGHSEQVAVSTRRSATTQVPNYPNLPSQLMCQVHNVTLHADKDSDEIYAQMSLQPVHSERDVFPAPDFGLLNRSKHPAEFFCKTLTASDTSTHGGFSVPRRAAEKLFPPLDYTAQPPTQELVVRDLHENTWTFRHIYRGQPKRHLLTTGWSLFVGSKRLRAGDSVLFIRDEKSQLMVGVRRANRQQTALPSSVLSADSMHIGVLAAAAHATANRTPFLIFYNPRACPAEFVIPLAKYRKAICGSQLSVGMRFGMMFETEDSGKRRYMGTIVGISDMDPLRWSGSKWRNLQVEWDEPGCNDKPTRVSPWDIETPESLFIFPSLTSGLKRQLHPSYFAAGETEWGSLIKRPLIRDSTNGILPYASFPNMASEQLMRMMMRPHNNNNNQNASSFMSEMQQNLLRGHGGLLGDMNMQQQPMVSEMVQPESKLTVNPSTSNTSGQEQNLFQSMSAPPKPTLSGCSSGRVNHGNEQKSVEQASQVRTVAVCNESALSPLQADPCPDTSQQIYPPQSDPINGFSFLETEELTSQVSSFQSLAGSYKQPSLLTSQDSSAVVLPDSTNSPLFHDVWDNQLKFDQFSPLMQQDLYGCQDSTTSNILDPPPLSNTVLDDFCAIKETDFQNHHPSDCLVGNSNTSFAQDVSQITSASFADSKAISRQDNSGGTTGTSSSNVDFDDTSLLQQNCKGSWQKLAATPRVRTYTKVQKTGSVGRSIDVTSFRDYNELKTAIECMFGLEGLLTRPQSSGWKLVYVDYESDVLLVGDDPWEEFVGCVRCIRILSPTEVEQMSEEGMKLLNSACINDLKSSVSK

**>BnaA08g31250D**

MGSLACGEDKMKTNGLVNGGTTTSTTSQSTLLEEMKLLKDQSGTRKPVINSMLWHACAGPLVCLPQVGSLVYYFSQGHSEQVAVSTRRSATTQVPNYPNLPSQLMCQVHNVTLHADKDSDEIYAQMSLQPVHSERDVLPVPDLGLLRGSKHPSEYFCKTLTASDTSTHGGFSVPRRAAEKLFPPLDYTAQPPTQELVVRDLHENTWTFRHIYRGQPKRHLLTTGWSLFVGSKRLRAGDSVLFIRDEKSQLMVGVRRANRQQTALPSSVLSADSMHIGVLAAAAHATANRTPFLIFFNPRACPAEFVIPLPKYRKAICGSQLSVGMRFGMMFETEDSGKRRYMGTIVGISDLDPLRWPGSKWRNLQVEWDEPGCNDKPTRVSPWDIETPESLFIFPSLTSGLKRQLHPSYFAGENDWGSLIKRPLPYATFPNMASEQLMKMMMRPHNNQNAVTSFMPEMQQNVLMGHGGLLGDVKMQQPMVMNQVVQVQPDNNNPSVSNTSGQEQNLSQSMNAPTNLENSSGRVNHGNEELSEKPSALSPLQADPSPEQIYPPQQSDPTNGFSFLETEEMTSQVSSFQSLAGSYKQPLMLSSNESSPIVLPDSTNSFQDMWDNQLNGLKFDQFSPLMQQEDLYGCQNMCMSNSTNSNILDPPPLSNTVLDDFCAIKETEFHSLVGNNNSFAQDVQSQITSASFADSQAFSRQDNSGGTGGTSSSNVDFDDTSLLQQNSKGSWQKLATPRVRTYTKVQKTGSVGRSIDVTSFRDYEELKTAIECMFGLEGLLTRPKTSGWKLVYVDYESDVLLVGDDPWEEFVGCVRCIRILSPTEVQQMSEEGMKLLNSACINDLKT

**>BnaA05g09790D**

MGGLIDLNVTEEQEEETTPSSGSGSLSPCDSSSSASAFGVSGSSSSSGVCLELWHACAGPLISLPKRGSLVLYFPQGHLEQAPDFSAAIYGLPPHVFCRILDVKLHAETATDEVYAQVSLLPESEDIERKVREGVIDVDGGEEDYEVVKRTNTPHMFCKTLTASDTSTHGGFSVPRRAAEDCFPPLDYTQPRPSQELLARDLHGLEWRFRHIYRGQPRRHLLTTGWSAFVNKKKLVSGDAVLFLRGDDGKLRLGVRRASQIEGASAFSSQYNQNMNHNNFAEVAHAISTNSAFNIYYNPKASWSNFIIPAPKFLKTVDYPFCIGMRFKARVESEDASERRSPGIITGINDLDPIRWPGSKWRCLLVRWDDTDANGHHQQRISPWEIEPSGSISSSGSFITTGPKRSRIGFSSGKPDIPVSGSGATDFEESLRFQRVLQGQEIFHGFINTSTDGGAGARRGRFKGTEFGDSYGFHKVLQGQETVPAYPMMTDQRHQGMSQRNIWCGPFQNFSTRILPPSSVHVSSPLPSSGPNVRMEEHHGGSGRCRLFGFPLRDETTAVGSGMVVGPCVEGQNGSSIRGASAVHTQGRDIYGMRDMLLDIAL

**>BnaA04g19890D**

MGGLIDLNVTEEEDETLSSASGSLSPSNSSSSAFVANGSSSSSSSSSGVCLELWHACAGPLISLPKRGSLVLYFPQGHLEQQAPGFSAAIYGLPPHVFCRILDVKLHAETDTDEVYAQVSLLPESEDIERKVREGVDGGDQEDYEVLKRTNTPHMFCKTLTASDTSTHGGFSVPRRAAEDCFPPLDYSKPRPSQELLARDLHGLEWRFRHIYRGQPRRHLLTTGWSGFVNKKKLVSGDAVLFLRGDDGKLRLGVRRASQFEGASAFSTQYNQNTNHNNFSEVAHAISTNSAFNIYYNPKASWSNFIVPAKKFLKIVDYPFCIGMRFKARVESEDASERRSLGIITGISDLDPIRWPGSKWRCLIVRWDDNEANRHQQRVSPWEIEPSGSISSSSSFITAGPKRSRIGFPDIPVSEGIHATDFEESLRFQRVLQGQEKFQGFINTSSDGGAGARRGRFKGTEFGDSYGFHKVLQGQETVPVYSMTAHQQHGLSQRNIWCGPFQNFSSRVLPPSSSPSSVLLTNSNGLNGGLEEHHGGSGSRCRLFGFPLRDESTAMVVPCVEGQKGVSDVQSNHHHHSQGRDMYGMRDMLLDIAL

**>Csa_6G518210**

MGALIDLNTTEEDESHSPAGSSVSSSSASALTSSPSPSVTSSICLELWHACAGPLTSLPKKGSLVVYLPQGHFEQMQEFPPTPYDLPPHILCRVIDVQLHQAEAGSDEVYAQVSLFPENEQMEHKMQEEMNNDSEEEDVEEGEKTTTPHMFCKTLTASDTSTHGGFSVPRRAAEDCFPPLDYNQQRPSQELVAKDLLGLKWKFRHIYRGQPRRHLLTTGWSAFVNKKRLVSGDAVLFLRGNDGELRLGIRRAAQLKSGSAFSNICSQQLNSSSIMDVVNAVSSKSSFSVCYNPRAASSQFVLPFHKFLKSINHSFSVGLRFRLSFETDDGADRRHTGHITGVSDVDPIRWPGSRWRSLMVRWDDGETNRHGRVSPWEIEPSGSVSLSTNLVPPGLKRTRIGLSSTKLEFPVPNGIGASDFGESLRFQKVLQGQEILGYSTPPDGDNNNRCPPEKRRLIPGLHGSGIALMRNGPRNPLINSETSSRGIVFDESFQYHKVLQGQEIFPSPYYGRAVATNEVKANGGCSPADGIRLPRTKDGWPMAMQCENFLTRSSIPSVQVSSPSSVFMFQQSMVPVQSFNSHNRGNFAEQRIMNKSTSHHSGTTFMTDHSMNTEFIPQGMCPTSLGEQNQLGLSHPSTTTASAAFTVSKDLSSTCKAGCRLFGFSLTEEKNVGNKDDKGSSATTPINAGTTTVLSNMGVQCPLKSPLMNKVVGSNCTKGAFQYHLANYNTYY

**>Csa_3G866510**

MGSVEEKLKTSGGLINNAPQTNLLDEMKLLKEMQDQSGARKAINSELWHACAGPLVSLPHVGSLVYYFPQGHSEQVAVSTKRTATSQIPNYPNLPSQLMCQVQNVTLHADKDSDEIYAQMSLQPVNSEKDVFLVPDFGLRPSKHPNEFFCKTLTASDTSTHGGFSVPRRAAEKLFPPLDYTMQPPTQELIVRDLHDNTWTFRHIYRGQPKRHLLTTGWSLFVGAKRLRAGDSVLFIRDEKSQLLIGVRRANRQQTTLPSSVLSADSMHIGVLAAAAHAAANRSPFTIFYNPRACPSEFVIPLAKYRKCVYGTQLSAGMRFGMMFETEESGKRRYMGTIVGISDLDPLRWPGSKWRNLQVEWDEPGCCDKQNRVSSWEIETPESLFIFPSLTSGLKRPLHGGFLAGETDWGSLVKRPMLRVPENIRGDLSYAPTLCSEPLMKMLLRPQMVNLNGTTLQQDSTNNLVKIQDMKDMQNPKMQQLIPTETASPGNQNQHHPGPAQSDPINPNSSPKANVPGKVQTSVAIESEAPTAADGDKAKYDRDLSASTNQSNPLPPVGGCAEEKLTSNEMNMQTLVNQLSFVNQNQIPMQLQSVSWPMQPQLESLIQHPQPIDMPQPEYTNSNGLISSLDGDGCLINPSCLPLPGVMRSPGNLSMLGLQDSSTVFPEVLNFPLPSTGQDMWDPLNNIRFSSQTNHLISFSHADASNLNCMANANIMRDVSDESNNQSGIYSCSNLEMSNGGSTLVDHAVSSTILDDYCTLKDADFPHPSDCLAGNFSSSQDVQSQITSASLGDSQAFSRQEFHDNSAGTSSCNVDFDEGSLLQNGSWKQVVPPLRTYTKVQKAGSVGRSIDVTSFKNYDELCSAIECMFGLEGLLNDPRGSGWKLVYVDYENDVLLIGDDPWEEFVSCVRCIRILSPSEVQQMSEEGMKLLNSAMMQGINCPMSEGGRS

**>GLYMA_14G217700**

MMASVEEKIKTGGGMIVGGQTLAAEMKLLKEMQEHSGVRKTLNSELWHACAGPLVSLPQVGSLVFYFPQGHSEQVAASTRRTATSQIPNYPNLPYQLLCQVQNVTLHADKETDEIYAQMTLQPLNSEREVFPISDFGHKHSKHPSEFFCKTLTASDTSTHGGFSVPRRAAEKLFPPLDYTIQPPTQELVVRDLHDNTWTFRHIYRGQPKRHLLTTGWSLFVGSKRLRAGDSVLFIRDERSQLRVGVRRVNRQQTTLPSSVLSADSMHIGVLAAAAHAAANRSPFTIFYNPRACPSEFVIPLAKYRKSVFGTQVSVGMRFGMMFETEESGKRRYMGTIVGISDVDPLRWPGSKWRNIQVEWDEPGCGDKQNRVSVWEIETPESLFIFPSLTSGLKRPLPSGLLENEWGTLLRRPFIRVPENGTMELSNSIPNLYSEHMMRMLLKPQLINNNGAFLSAMQQESAATRGPLQEMKTTLAAENQMPLKNLHPHSIPDQPNALNMQSLLKNDQPEKLHPLGKIDNHLSSGIVIDKPKSESEVLPDHVIDYPSMEGCNIEKVAANPVNQQGLANQLPFHNQNQSPLLPQSSPWPMPPQIELSMPHPQMIDMVQADSAMVNGLFPQLDINEWMSYASSQPFAGQNRPTGPLSDLQEHTSLQPQVVNPPLPSMNNEVWDHYVKNLKFLSQADQLTSICQPGLYGLNGIPSSNNLRDLSAESNNQSEICVNVDASNSVGTTVVDPSTSSTILDEFCTMKDREFQNPQDCMVGNLSSSQDVQSQITSASLTESHAFPLRDIPDNSGGTSSSHVDFDESSFLQNNSWQQVPAPIRTYTKVQKAGSVGRSIDVTTFKNYEELIRAIECMFGLDGLLNDTKCSGWKLVYVDYESDVLLVGDDPWEEFVGCVRCIRILSPSEVQQMSEEGMKLLNSGALQGMNV

**>GLYMA_17G256500**

MMASVEEKIKTGGVGGGMVVGGQTLVAEMKLLKEMQEHSGVRKTLNSELWHACAGPLVSLPQVGSLVFYFPQGHSEQVAASTRRTATSQIPNYPNLPSQLLCQVQNATLHADKETDEIYAQMTLQPLNSEREVFPISDFGLKHSKHPSEFFCKTLTASDTSTHGGFSVPRRAAEKLFPPLDYTIQPPTQELVVRDLHDNTWTFRHIYRGQPKRHLLTTGWSLFVGSKRLRAGDSVLFIRDEKSQLRVGVRRVNRQQTTLPSSVLSADSMHIGVLAAAAHAAANRSPFTIFYNPRACPSEFVIPLAKYRKSVFGTQVSVGMRFGMMFETEESGKRRYMGTIVGISDVDPLRWPGSKWRNIQVEWDEPGCGDKQNRVSVWEIETPESLFIFPSLTSGLKRPLPSGLLENEWGTLLTRPFIRVPENGTMELSNSIPNLYSEHMMKMLFKPQLINNNGAFLSAMQQESAATRGPLQEMKTTLAAENQMLLKNLHPQSIPDQPNALNMQSLLKNDQPEKFHPLAKIDNHLPSGIVIDKPKLECEVLPDRVIDYPSMEGCNNEKVAVNPVNQQGLATQLPFHNQNQSPLLPQSSPWPMQPLIESSMPHPQMIGMAQADSAMVNGLFPQLDIDEWLAYASSQPFAGQNRPTGPFSDLQEHNSLQPQVVNPPLPSMNNEVWDHYVKNFKFLSQADQLTSICQPGMYGLNGVPSSNNLRDLSAESNNQSEICVNVDASNSVGTTMVDPSTSSTILDEFCTMKDGEFQNPQDCMVGNLSSSQDVQSQITSASLAESHAYPLRDIPDNSGGTSSSHVDFDESSFLQNNSWQQVPAPIRTYTKVQKAGSVGRSIDVTTFKNYEELIRAIECMFGLDGLLNDTKGSGWKLVYVDYESDVLLVGDDPWEEFVGCVRCIRILSPSEVQQMSEEGMKLLNSGALQGMNV

**>GLYMA_13G234200**

MGLIDLNTTEDDEAAPLSASSPASYSSSSSHSGISTSASTLVVPPTPPSVCLELWHACAGPLISLPKKGSVVVYLPQGHFEHVQDFPVTAYDIPPHVFCRVLDVKLHAEEGSDEVYCQVLLVPESEQVEQSLREGEIVADGEEEDTEAIVKSTTPHMFCKTLTASDTSTHGGFSVPRRAAEDCFPPLDYSQQRPSQELVAKDLHGLEWRFRHIYRGQPRRHLLTTGWSAFVNKKKLVSGDAVLFLRGDDGELRLGIRRAAQLKSAGSFAVPSGQQLNPATLKGVVNALSTRCAFSVCYNPRFSSSEFIIPVHKFLKSLDCSYSVGMRFRMRFETEDAAERRCTGLIAGISDVDPVRWLGSKWRCLLVRWDDIEAARRNRVSPWEIEPSGSASNSSNLMSAGLKRTRIGMTSVKLEFPTPDGIGASDFGESLRFRKVLQGQEILGVNTPFDGINAQSPRLYELGRCYPGSNCSGIPPTGNNIRMPHPASDFSCNGIGFSESFRFQKVLQGQEILPSQPYGRVLSIEEARANGCYGLYDGCQLLNTRNGWSAQMHDNAPYLHASVTPAQVSSPSSVLMFQQAVNPVSNCDYNKNKRDKEMEGRVHYQGLYTSEVKGRTFASSPFDDPILSTLAPEGASSLGMFDVHNQLGSSRPHESVSALRSSQELVSSCKSSCRLFGFSLTEDTHLAKKEASASTITLPLSSGPSFTRLVEDEFHPGHSLPSKGVGSNCTKGVLQY

**>GLYMA_07G202200**

MAGLIDLNNATEDDEMPSSGSSSTVCLELWHACAGPLISLPKKGSVVVYFPQGHLEQHLHDFPLPASANIPSHVFCRVLDVKLHAEEGSDEVHCQVVLVPETEQVHQKLREGEFDADGEEEDAEAVMKSTTPHMFCKTLTASDTSTHGGFSVPRRAAEDCFPPLDYSQQRPSQELVAKDLHGQEWRFRHIYRGQPRRHLLTTGWSAFVNKKKLVSGDAVLFLRGEDGELRLGIRRAAQLKSGSTFSALSGQQLSPTSLMDVVNALSARCAFSIHYNPRVSTSEFIIPIHRFLKSLDYSYSAGMRFRMRFETEDAAERRFTGLIVGIADVDPVRWPGSKWRCLMVRWDDLEVTRHNRVSPWEIEPSGSASTANNLMSAGLKRTKIGLPSAKLEFPVSNAIGTSDFGESLRFQKVLQGQEMLGVNPTYDSINAQSHQVSDLRRCYPVSNYPRIAATGNSIGIPQVSSNVSSNGIGFSESFRFQKVLQGQEIFPSQPYGRALSVDEACGNSRCGLFDGYQVMRSRNGWCSDMSNNSSNLHPPVPSGQVSSPSSVLMFQQAVNLVSNSDYNNKISQVMGGKFHQRVSYASDVKGGKFVSTPYEPLLCGLAKEGTNSFGLSNFHNQLDSSRSHDSISVLRANQELVPSCKSSCRVFGFSLTEVAPIANKEAADSSSVACSGPSFTRHVDDDFHPGHSLRSKAVASYCTKGVLQY

**>GLYMA_15G078800**

MGLIDLNTTEDDEAAPLSASSSHSGISTSASTLVVSPPPSVCLELWHACAGPLISLPKRGSVVVYLPQGHFEHVQDFPVNAFDIPPHVFCRVLDVKLHAEEGSDEVYCQVLLVPESEQVEHSLREGEIVADGEEEDTGATVKSTTPHMFCKTLTASDTSTHGGFSVPRRAAEDCFPPLDYSQQRPSQELVAKDLHGLEWRFRHIYRGQPRRHLLTTGWSAFVNKKKLVSGDAVLFLRGNDGELRLGIRRAAQLKWAGSFAVPSGQQLNPATLMDVVNALSTRCAFSVCYNPRFSSSEFIIPVHKFLESLDCSYSVGMRFRMRFETEDAADRRFTGLIAGISDVDPVRWPGSKWRCLLVRWDDIEAARHNRVSPWEIEPSGSASNSSNLMAAGLKRNRIEMTSAKLEFPNPNGIQTSDFGESLRFRKVLQGQEILGVNTPPFDGINAQSPWLYELGRCYPGSNCSGIPPTGNNIRVPHPASDFSCNGIGFSESFRFQKVLQGQEILPSQPYGRALSIEEARANGRYGFYDGCQLLNSRNGWSAQMHDNASHLHASVTPAQVSSPSSVLMFQQAVNPVSNCDYNINKCNKEMEGRIHYQGLYTSEMKGRTLASSPSDDLIVSRRAPEGASSIGMFDVHNQLGSSQLHESVSALRSSQKLVSSCKSSCRLFGFSLTEDTHVANEEVSASTITLPLSSGPSFTRLVEDEFHPGHSHPSKAVGSNCTKSVLQY

**>GLYMA_13G174000**

MAGLIDLNNATEDDETPSSGSSSSSSSSTVCLELWHACAGPMISLPKKGSVVVYFPQGHLEQHLHDFPLPASANIPSHVFCRVLDVKLHAEEGSDEVYCQVVLVPESEQVQQKLREGEFDADGEEEDAEAVMKSTTPHMFCKTLTASDTSTHGGFSVPRRAAEDCFPPLDYSQQRPSQELVAKDLHGQEWRFRHIYRGQPRRHLLTTGWSAFVNKKKLVSGDAVLFLRGEDGELRLGIRRAAQLKSGSTFSALSGQQGSPTSLMDVVNALSARCAFSIHYNPRVSSSEFIIPIHRFVKSLDYSYSAGMRFRMRFETEDAAERRFTGLIVGIADVDPVRWPGSRWRCLMVRWDDLEATRHNRVSPWEIEPSGSASTANNLMSAGLKRTKIGLPSAKLDFPVSNAIGTSDFGESLRFQKVLQGQEMLGVNTTYDSFNAQSHQLSDLRRCYPGSNYPRIAATGNSIGISQVSSNVSNNGIGFSESFRFQKVLQGQEILPSQPYGRALSVDEACGNGRFGLFDGFQAMRSRNGWSSHVSNNSSHLHPPVPSGQVSSPSSVFMFQQAVNPVLNSDYNNQIGQVMGDKVHQRVSYASEVKGGKFVSTPYEPLLRGLSQEGTNSFGLSNFHNQLDSSRSHDSVSVLRASQELVPSCKSRCRVFGFSLTEGAPVASKEATDSSAVACAGPSFTRHVEDDFHPGHSLRSKAVASYCTKGVLQY

**CYTOKININ OXIDASE 3 (CKX3) and CKX5**

**>AT5G56970 (AtCKX3)**

MASYNLRSQVRLIAITIVIIITLSTPITTNTSPQPWNILSHNEFAGKLTSSSSSVESAATDFGHVTKIFPSAVLIPSSVEDITDLIKLSFDSQLSFPLAARGHGHSHRGQASAKDGVVVNMRSMVNRDRGIKVSRTCLYVDVDAAWLWIEVLNKTLELGLTPVSWTDYLYLTVGGTLSNGGISGQTFRYGPQITNVLEMDVITGKGEIATCSKDMNSDLFFAVLGGLGQFGIITRARIKLEVAPKRAKWLRFLYIDFSEFTRDQERVISKTDGVDFLEGSIMVDHGPPDNWRSTYYPPSDHLRIASMVKRHRVIYCLEVVKYYDETSQYTVNEEMEELSDSLNHVRGFMYEKDVTYMDFLNRVRTGELNLKSKGQWDVPHPWLNLFVPKTQISKFDDGVFKGIILRNNITSGPVLVYPMNRNKWNDRMSAAIPEEDVFYAVGFLRSAGFDNWEAFDQENMEILKFCEDANMGVIQYLPYHSSQEGWVRHFGPRWNIFVERKYKYDPKMILSPGQNIFQKINSS

**>AT1G75450 (AtCKX5)**

MNREMTSSFLLLTFAICKLIIAVGLNVGPSELLRIGAIDVDGHFTVHPSDLASVSSDFGMLKSPEEPLAVLHPSSAEDVARLVRTAYGSATAFPVSARGHGHSINGQAAAGRNGVVVEMNHGVTGTPKPLVRPDEMYVDVWGGELWVDVLKKTLEHGLAPKSWTDYLYLTVGGTLSNAGISGQAFHHGPQISNVLELDVVTGKGEVMRCSEEENTRLFHGVLGGLGQFGIITRARISLEPAPQRVRWIRVLYSSFKVFTEDQEYLISMHGQLKFDYVEGFVIVDEGLVNNWRSSFFSPRNPVKISSVSSNGSVLYCLEITKNYHDSDSEIVDQEVEILMKKLNFIPTSVFTTDLQYVDFLDRVHKAELKLRSKNLWEVPHPWLNLFVPKSRISDFDKGVFKGILGNKTSGPILIYPMNKDKWDERSSAVTPDEEVFYLVALLRSALTDGEETQKLEYLKDQNRRILEFCEQAKINVKQYLPHHATQEEWVAHFGDKWDRFRSLKAEFDPRHILATGQRIFQNPSLSLFPPSSSSSSAASW

**>BnaAnng09190D**

MTSSFLLMTFAICTLIIAVGPSELLRIGAIDVDGHFTFKPSDLASVSSDFGMLKSPEEPLAVLHPSSAEDVARLIRTAYGSATAFPVSARGHGHSINGQASTGRNGVVVEMSHRNIGTPEPLVEPEEMYVDVWGGELWVDVLKKTLEHGLAPKSWTDYLYLSVGGTLSNAGISGQAFHHGPQISNVLELDVVTGRGEVMRCSEEENTRLFHGVLGGLGQFGIITRARISLEPAPQRVRWIRVLYSSFEVFTEDQEYLISMHGQSKFDYVEGFVIVDEGLVNNWRSSFFSPRNPVKISSVSSNGSVLYCLEITKNYHESESDTVDQEVEILMKKLNFIPTSVFTTDLQYVDFLDRVHKAELKLRSKNLWEVPHPWLNLFVPKSRISDFDKGVFKGILGNKTSGPILIYPMNKDKWDERSSAVTPDEEVFYLVALLRSALSDGEETQKLEYLKDQNRRILEFCEQAKINVKQYLPHHATQEEWVAHYGDKWDQFRSMKAEFDPRHILATGQKIFQNPSLALFRPSSSSSSASW

**>BnaA02g08420D**

MASNNFPSQSHLLVIIIFITTLLTPITTNTSPQPWNILSNDNFAGKLTSASSSVEAASIDFGHVTKILPSAVLNPSSVQDIIDLIKLSFDSQSSFPIAARGHGHSFRGQAAAKDGVVVNMRSMVNEDRGIKVSRTGLYADVDTAWLWIEVLNKTLELGLTPVSWTDYLYLTVGGTLSNGGISGQTSRYGPQISNVLELDIITGKGEIATCSNDMNSDLFYAALGGLGQFGIITRARIKLELAPKRAKWLRFLYTDFSEFTRDQERLISEAGGLHFLEGSVMLDHGPPDNWRSTYYPPSDHLRIVSMIKRHRVIYCLEVVKYYDEFSQHSVNEEMEELSESLNFVRGFVYEKDVTYIDFLNRVRTGELNLKSKGQWDVPHPWLNLFVPKSQISRFDYGVFKGIILRNNITTGPLLVYPMKRIMWNDQMSTAIPEEDVFYAVGFLRSAEFDNWEAYDKENMEVLMFCEDAKMDVIQYLPYHASQEGWVRHFGPRWNTFVERKYKYDPKMILSPGQNIFQ

**>BnaA10g28940D**

MVSYNFPSQIHLLMITILVIITTLLTPITTNTSSLPWNILSNDNFAGKLTTASSSVESAATDFGHITKIFPSAVLNPSSVQDITDLIKLSFDSQSSFPLAARGHGHSHRGQAAAKDGVVVNMRSMVNRDRGIKVSRTGLYADVDSAWLWIEVLNKTLELGLTPVSWTDYLYLTVGGTLSNGGISGQTFRYGPQISNVLEMDVITGKGEIATCSKDMNSDLFYAALGGLGQFGIITRARIKLELAPKKAKWLRFLYTDFSEFTRDQERLISETNGPHFLEGSVMLDHGPPDNWRSTYYPPSEHMRIVSMVKQHRVIYCLEVVKYYDETSQHSVNEEMEELSDSLNYERGFVYEKDVTYMDFLNRVRTGELKLKSKGKWDVPHPWLNLFVPKSQISRFEYGVFKGIILRNNITTGPLLVYPMNRNKWNDRMSTVIPEEDVFYAVGFLRSASFDNWDDYEKENMELFPPEYDSTKSRFSAL

**>Csa_1G588560**

MNENLPVPAYFIITFFISRLKSSINKSKAWTTTTTVNQQPNNLQNDPETLTIASSDYGNMVKETPAAVLEPSSINDVVQLISYAYNNPIPFHIAARGQGHSVRGQAMAKNGVVIDMSALRRNRKTPGIVVSCRRWTTGEFYVDVGGEQLWIEVLNATLGYGMTPVSWTDYLYITVGGTLSNAGISGQSFRYGPQVSNVVEMDVVTGKGNMMTCSPRKNCELFHAVLGGLGQFGIIARARIALEPAPTRVKWVRMLYTNFVAFTKDQEHLISLNGRKQVNALNYLEGLVLLHHGSPDNWRSSFFPLSDHSRIISLANQNSVIYCLEVVKYYDHHTQSTVDKDLEVLLEGLDYESGFKFEKDVTYVEFLNRVRSGELKLQSKGLWDVPHPWLNLFVPRSRIIDFDSGVFKDIIVRRNITKGPILIYPMNRSKWDDRNSTVIPDEEVFYTVGFLNSSGFDDWKKFEEQNEEILEYCEKSGIEIKQYLPHYKTQTQWIHHFGSKWTTFQHNKFKFDPKHILSPGQKIFNS

**>Csa_2G362450**

MITTKLLLAFSICRLIATVGLNMGPATELPRLGIDGLLTVDPLDIETASIDFGLMTRDPPLAVLHPASADDVAKLIRTVANAAEEENGGFTVSARGHGHSINGQAQTGNGVVIEMSGGGRQHRRVGGRGASPPLPVVSEKGRFVDVWGGELWIDVLKWTLEYGLAPRSWTDYLYLSVGGTLSNGGISGQAFNHGPQISNVHELDVVTGNGEIVKCSNEENADLFHGVLGGLGQFGIITRARIVLEPAPQRVRWIRVLYSNFEAFTKDQEWLISLHSKTNSNEKFDYVEGFVIVDEGLINNWRSSFFSPANPVKISSFNKNKSHGAVLYCLEITKNYHESSSHTLDQEVEALMKELNYIPESVFTTDLGYVDFLDRVHKAELKLRSKGLWDVPHPWLNLFVPKSRIADFDRGVFKGILGNNTSGPILIYPMNKHKWDPRTSAVTPEDDVFYLVALLRSALDNGEPTQSLEYLSHQNHQILEFCYENGIEVKQYLPHYTKEEEWADHFGDKWPEFQARKLKFDPHHILATGQRIFPAFKPVNMAVSR

**>GLYMA_04G028900**

MAVNNNKLVLLTFAICRLIVTVGLTVVPELLDVGLQGRLSVDTLELEAASVDFGRLSRGEPSEVVHPATAEDVARVVKAAFESPFAVSARGHGHSINGQAMIKEKKGVVIEMGKSDSGEDGSSIRVSEKGMYVDVWGGKLWIDVLSATLEYGLAPMSWTDYLYLSVGGTLSNAGISGQTFNHGPQITNVYELDVVTGKGELVTCSEDRNSELFHAVLGGLGQFGIITRARIALEPAPHRVRWIRVLYSNFARFCKDQEYLISLHGKAARERFDYVEGFVIVDEGLINNWRSSFFSASNPVKITSLNADGGVLYCLEITKNYDQGNADSVDEEIQALLKKLNFIPTSVFTTDLPYVDFLDRVHKAELKLRSKGLWDVPHPWLNLFVPKSRIGDFDKGVFKGILGNKTSGPILIYPMNKNKWDQRSSVVTPEEDVFYLVAFLRSALDTETLEYLTNQNRQILKFCHDIEIKVKQYLPHYTTQQEWMDHFGDKWTQFNARKMQFDPRRILATGQQIFQFHPSLTLNM

**>GLYMA_17G054500**

MALNYPFLTYFILLLVTITRLIFTVGKTEQWKAPILPELDIDNISHKLHDDPETIQMASRDYGHLTHEFPLAVFRPSSIDDIVTLIKSSYNSFAPFDIAARGQGHSTHGQAMARDGIVVDMASLRKQRNGVAISVSKDPLMGHYADVGGEQLWIDVLHATLEYGLAPVSWTDYLYLTVGGTLSNAGISGQSFRYGPQISNVHEMDVITGKGEFVTCSSQKNLELFHAVLGGLGQFGVIARARIALEPAPKRVKWVRLLYSDFSAFTKDQERLISINGRKQKNALDFLEGMLLMNQGPINNWRSSFFPLSDHPRIASLITEHSILYCLEVAKYYDEQTELNVDKEIEVLLQGLAYIPGFNYEKNVSYVEFLNRVRSGELKLQSQGLWEVPHPWLNLFIPKSQILDFNSGVFKDIVLKRNISSGPVLVYPMNRNKWDDRMSASIPDEDVFYTVGFLHSSGFDTWKAYDAQNREILEFCRDAGIMVKQYLPNHSTQEDWTNHFGAKWMKFLERKHQFDPRMILSPGQKIFHKKLQPVF

**>GLYMA_13G104700**

MALHYPFPTYFILLLVTITRLIYTVGKTEQWKAPILTELDINNISHKLHDDPEIIQMASRDYGHIVHEFPLAVFRPSSIDDIATLIKSSYNSFAPFGIAARGQGHSTHGQAMARDGVVVDMANLRKQRNGVAISVSKDPLMGHYADVGGEQLWIDVLHTTLKHGLAPVSWTDYLYLTVGGTLSNAGISGQSFRYGPQISNVHEMDVITGKGEFVTCSSQKNLELFHAVLGGLGQFGVIARARIALEPAPKRVKWVRLLYSDFFAFTKDQERLISINGRKQKNALDFLEGMLLMNQGPINNWRSSFFPLSDHPRISSLITEHSILYCLEVAKYYDEQTEINVDKEIQVLLQGLAYIPGFYYEKNVSYVEFLNRVRSGELKLQSQGLWDVPHPWLNLFIPKSQILDFNSRVFKDIVLKRNISSGPVLVYPTNRNKWDDRMSASIPDEEVFYTVGFLHSSGFDTWKAYDAQNSEILEFCRDAGIKVKQYLPNHSTQEDWTNHFGAKWIKFLERKHQFDPRMILSPGQKIFHKQLQPAF

**>GLYMA_09G063900**

MVAGKYPSPTYFILLLITITRLISTVGKTSQWMKALTPPPELASVSLDDTIFSKLRNDPEALQGRASRDYGNLVREVPSAVFHPTSSSDIARLIKLSYNGSVPFKIAARGQGHSTRGQAMVRDGVVVDMAGFRERGNGEGIRVVMSVVVDPNNKNGYGYYYADVGGEQLWIDVLNATLEHGLAPMSWTDYLYLTVGGTLSNAGISGQTFRYGPQITTVRQMDVITGKGEFVTCSQQTNSELFHAVLGGLGQFGIITRARIALAPAPKRVKWVRLLYNDFSAFTKDQEQLISITRRKQNIALDYLEGLLLMHQGPINNWRSSFFPLADHARIISLVTKHSVLYCLEVAKYYDGQNENNVDKELKVLLQGLSYIPGFYYEKDVSYVEFLNRVRSGELKLQSQGLWDVPHPWLNLFIPKSQIMEFDSGVFKNIILKRNITTGPVLVYPMNRNKWDNRMSASIPDEDIFYTVGFLHSSGFDNWKAYDAQNKEILQFCNDSGIKVKQYLPHYRTQEDWTNHFGPKWRTFVERKHQFDPKMILSPGQRIFNN

**>GLYMA_15G170300**

MQIKVLFHSIYSPYLLHLLSPLQHTNTNNQINPSTLETTKHSLFSHSIIFSHPLILSKPTKKKMVAENYPSPTYFILLFITITRLISTVGKTSQWTKALSLTPELASVSLDDTIFCKLRDDPEALQGRASRDYGNLVREVPLAVFHPASASDIARLIKLSYNGSVPFKIAARGQGHSTRGQAMAREGVVVDMAGFRERGNGVGIRVVSSVDPNNKNGYYYYADVGGEQLWIDVLHATLEHGLAPMSWTDYLYLTLGGTLSNAGISGQTFRYGPQITTVREMDVITGKGEFVTCSQQTNSELFHAVLGGLGQFGIITRARIALAPAPKRVKWVRLLYNDFSAFTKDQEQLISVTGRKQNVSLDYLEGLLLMHQGPINNWRSSFFPLADHARIISLVTKHSVLYCLEVAKYYDGQNENNVDKELQVLLQGLSYIPGFYYEKDVSYFEFLNRVRSGELKLQSQGLWDVPHPWLNLFIPKSQIMEFDSGVFKNIILKRNITTGPVLVYPMNRNKWDNRMSASIPDEDIFYTVGFLHSSGFDNWKAYDAQNKEILQFCNVAGIKVKQYLPHYRTQEDWANHFGPKWRTFVERKHQFDPRMILSPGQRIFNN

**ARABIDOPSIS HISTIDINE KINASE 3 (AHK3)**

**>AT1G27320 (AtAHK3)**

MSLFHVLGFGVKIGHLFWMLCCWFVSWFVDNGIEDKSGLLVGSVGDLEKTKMTTLKKKNKMWFWNKISSSGLKIPSFSYQFLGSVKFNKAWWRKLVVVWVVFWVLVSIWTFWYFSSQAMEKRKETLASMCDERARMLQDQFNVSMNHVQAMSILISTFHHGKIPSAIDQRTFSEYTDRTSFERPLTSGVAYAMRVLHSEREEFERQQGWTIRKMYSLEQNPVHKDDYDLEALEPSPVQEEYAPVIFAQDTVSHVVSLDMLSGKEDRENVLRARSSGKGVLTAPFPLIKTNRLGVILTFAVYKRDLPSNATPKERIEATNGYLGGVFDIESLVENLLQQLASKQTILVNVYDITNHSQPISMYGTNVSADGLERVSPLIFGDPLRKHEMRCRFKQKPPWPVLSMVTSFGILVIALLVAHIIHATVSRIHKVEEDCDKMKQLKKKAEAADVAKSQFLATVSHEIRTPMNGVLGMLHMLMDTELDVTQQDYVRTAQASGKALVSLINEVLDQAKIESGKLELEEVRFDLRGILDDVLSLFSSKSQQKGVELAVYISDRVPDMLIGDPGRFRQILTNLMGNSIKFTEKGHIFVTVHLVDELFESIDGETASSPESTLSGLPVADRQRSWENFKAFSSNGHRSFEPSPPDINLIVSVEDTGVGIPVEAQSRIFTPFMQVGPSISRTHGGTGIGLSISKCLVGLMKGEIGFSSTPKVGSTFTFTAVFSNGMQPAERKNDNNQPIFSEFRGMKAVVVDHRPARAKVSWYHFQRLGIRVEVVPRVEQALHYLKIGTTTVNMILIEQEIWNREADDFIKKLQKDPLFLSPKLILLANSVESSISEALCTGIDPPIVIVKPLRASMLAATLQRGLGIGIREPPQHKGPPALILRNLLLGRKILIVDDNNVNLRVAAGALKKYGADVVCAESGIKAISLLKPPHEFDACFMDIQMPEMDGFEATRRIRDMEEEMNKRIKNGEALIVENGNKTSWHLPVLAMTADVIQATHEECLKCGMDGYVSKPFEAEQLYREVSRFFNSPSDTES

**>BnaA07g08880D**

MSLFHVLGFCLKIGQLFWMLCCWFLSWFLSWFLDADKSPLDPDKTKMKNHNKMCFLWNKISTSGLKIPPSFSHHLFGSVRFGKTFWRKVLVAWVVSWVLISFWTFWCLTSQAMAKRKETLASMCDERARMLQDQFNVSMNHVQAMSILISTFHHSKFPSAIDQRTFSEYTDRTSFERPLTSGVAYAVRVLHSERQEFERQQGWTIRRMEQNPVHKDDYDTEALEPSPVQQEYAPVIFAQDTVSHVISLDMLSGKEDRENVLRARRSGKGVLTAPFPLIKTNRLGVILTFAVYKRDLPSNATPNERIEATNGYLGGVFDIETLVENLLQQLASKQTILVNVYDTTNHSQPISMYGSHVSADGLEHVSPLNFGDPFRKHEMRCRFKQKPPWPVQSMVTSFGILVIALLVAHIFHATLSRIRRAEEDCHKMELLKKKAEAADVAKSQFLATVSHEIRTPMNGVLGMLHMLMDTELDVTQQDYVRTAQASGKALVSLINEVLDQAKIESGKVELEEVRFDLRGILDDVLSLFSGKSQEKGLELAVYISDRVPEMLIGDPGRFRQILTNLMGNSIKFTEKGHIFVTVHLVEELLDSSDVETSSSSSTENTLSGLPVADRKRSWQNFKAFSSNGHRSLALAPSEINLVVSVEDTGVGIPVEAQSRIFTPFMQVGPSISRTHGGTGIGLSISKCLVGLMKGEIGFSSTPKVGSTFTFTVVFANGVHSTERKSELHNNNQPEFEGMNAVLVDHRPARAQVSWYHFQRLGIRVEVVTSVDQALRFMKTYATTVNMILIEQEVWNKEADVFVKDPLVHSPKLFLLANSIDTSVSDTLSNVIDPPVLIVKPLRASMLAATLQRGLGIGKRETPQRKGPPALILRNLLLGRKILIVDDNNVNLRVAAGALKKYGADVVCAESGVKAVSLLKPPHEFDACFMDIQMPEMDGFEATRRIRGMEEEMNNGEALTVEEGKRSRWHLPVLAMTADVIQATQEECLKCGMDGYVSKPFEAEQLYREVSRFFNSPSDTES

**>BnaC07g11340D**

MSLFHVLGFCLKIGQLFWMLCCWFLSWFVDADKFPLPVGSVGDPDKTKMKNHNMCFFWNKISTSGLKIPSFSHHLFGSVRFGKTFWRKVLVAWVVSWVLISFWTFWCLSSQAMDKRKETLASMCDERASMLQDQFNVSMNHVQAMSILISTFHHAKFPSAIDQRTFSEYTDRTSFERPLTSGVAYAVRVLHSERQEFERQQGWTIRRMDSLEQNPEYAPVIFAQDTVSHVISLDMLSGKEDRENVLRARRSGKGVLTAPFPLIKTNRLGVILTFAVYKRDLPSNATPKERIEATNGYLGGVFDIETLVENLLQQLASKQTILVNVYDTTNHSQPISMYGSDVSADVLEHVSQLNFGDPFRKHEMRCRFKQKPPWPVQSMVTSFGILVIALLVAHIFHATLSRIRRAEEDCHKMELLKKKAEAADVAKSQFLATVSHEIRTPMNGVLGMLHMLMDTELDVTQQDYVRTAQASGKALVSLINEVLDQAKIESGKVELEEVRFDLRGILDDVLSLFSGKSQEKGLELAVYISDRVPEMLIGDPGRFRQILTNLMGNSIKFTEKGHIFVTVHLVEELLDSSDVEASENTLSGLPVADRQRSWQNFKAFSSNGHRGLAPAPSEINLIVSVEDTGVGIPVEAQSRIFTPFMQVGPSISRTHGGTGIGLSISKCLVGLMKGEIGFSSTPKVGSTFTFTAVFANGVHSTERKSELHNNNKPEFEGMKAVLVDHRPARAQVSWYHFQRLGIRVELVPSVDQALRFMKTCATTVNMILVEQEVWNKEADVFVKEPLVHSPKLFLLANSIDTSVSDTLSNVIDPPVLIVKPLRASMLAATLQRGLGIGNRETPQRKGPPALILRNLLLGRKILIVDDNNVNLRVAAGALKKYGADVVCAESGVKSISLLKPPHEFDACFMDIQMPEMDGFEATRRIRGMEEEMNNGKALTEEEVKRSRWHLPVLAMTADVIQATHEECLKCGMDGYVSKPFEAEQLYREVSRFFNSPSDTES

**>Csa_6G095330**

MNWFINGGVMETKAGLLGGGGKIWLQLWETVIGNCCKMYHQYYQYIGSKKVKKTWWRRLLVAWVLSSILASLWIFHYMSSQATEKRKEALGSMCDERARMLQDQFNVSMNHIQAMSILISTFHHGKNPSAIDQRTFARYTERTAFERPLTSGVAYAVRVLHSDRERFEKQQGWTIKRMDKIEQSPVHEDDYAPEDLEPSPTQDEYAPVIFAQDTISHVVSLDMLSGVEDRNNVLRARASGKGVLTAPFKLIKTNRLGVILTFAVYKRDLPSNATPNERIQATDGYLGGVFDIESLVEKLLQQLASNQTILVNVYDTTNQSHPISMYGKDVSEDGLQHVSPLNFGDPDRKHEMRCRFKQKQPWPWLAMTTSIGILIIALLLGYIFHATLNRIAKVEDDYHEMMVLKKRAEDADIAKSQFLATVSHEIRTPMNGVLGMLHLLMDTDLDVTQQDYVKTAQDSGKALVSLINEVLDQAKIESGKLELEAIPFNLRADLDDILSLFSGKSQEKGLELAVYVSDSVPETLVGDPGRFRQIITNLVGNSIKFTEKGHIFVTVNLVKEVIESIDLEIESSKNSTLSGYPVANRRLSWAGFRTFSQEGSTACHFMTSPPDLINLMVSVEDTGVGIPLEAQSRIFTPFMQVRPSISRTHGGTGIGLSISKCLVGLMKGEIGFVSVPKIGSTFTFTAVFTNCSNSSEYNNTQQIKNTSISATSEFKGMRALVVDHQPIRAKVSRYHIQRLAINVEVLSDLNQCLSTTTISGSTVNMIFVEQKLWDQNVSTSDHFIKNLRNSYAVPPKLFLLTSSISSSKASTTVSDVFTPTVILKPLRAGMLAASLHRVMNVGIKGNPRNGELPVLSLRNLLLGRKILVIDDNKVNRIVAAGALQRYGADVVCENSGRDAIQLLTPPHHFDACFMDIQMPEMDGFEATRRIREIEHRINDGIQVGELSKEAYENTCYWRVPILAMTADVIQATHEECLRCGMDGYVSKPFEVERLYREVSQFFHSTSNGTL

**>GLYMA_08G105000**

MSLLHVVGFGLKVGHLLLVLCCWVVSVVYLNWFLSSGIIMDTKMGGGGGGGSKMWHKKWWEKISGQGCKIHQQYYQYIGSKKVKRALWRKLLLTWVVGWFIVSLRIFCYMSSQGTEKRKETLASMCDERARMLQDQFNVSMNHIQAMSILISTFHHAKSPSAIDQKTFAKYTERTAFERPLTSGVAYAVRVLHSEREQFEKQQGWTIKRMDTLEQNPVHKDDYAPEALEPSPVQEEYAPVIFAQDTIAHVISVNVLSGKEDRENVLRARESGKGVLTAPFRLLKTNRLGVILTFAVYKRDLPSNTTPNERIQATDGYLGGVFDVESLVEKLLQQLASKQTVIVHVYDTTNRTHPIAMYGSNESGDFFYHVSTLNFGDPFRKHEMHCRFKQKPPWPWVAITTSIGILVIALLVGYIFHATVNRIAKVEDDYREMMELKKRAEAADVAKSQFLATVSHEIRTPMNGVLGMLHMLMDTDLDVTQQEYVRTAQESGKALVSLINEVLDQAKIEFGKLELEAVLFDIRAILDDVLSLFSEKSQGKRVELAVYVSDHVPELLIGDPGRFRQIITNLMGNSIKFTDKGHIFVTIHLVEEVVRSIEVDKESNSENTLSGSPVADSRRSWEGFKAFSQEGPLGSFSSPSNDLVNLIVSVEDTGEGIPLESQPLIFTPFMQVGSSISRKHGGTGIGLSISKCLVGLMNGEIGFVSIPKIGSTFTFTAVFTNGHRSSSECKIQQINNQPQSASSEFEGMTALIIDPRSVRAEVSGYHIQRLGIHVEMVSDLKQGLSTISNGNVVVNMVLIEQEVWDRDLGLSSHFVNNTRRIDHGVPPKLFILVNSSSSFKASVNLGVHNPTVITKPLRASMLAASLQRAMGVQNKGAPHRELQSLSLRHLLRGRKILIVDDNGVNRAVAAGALKKYGADVVCVSSGKDAISSLKPPHQFDACFMDIQMPEMDGFEATKRIREMEDSVNREVSMDDFENITNWHVPILAMTADVIQATHEECLRCGMDGYVSKPFEAEQLYREVSRFFQSS

**>GLYMA_05G148100**

MSLLHVVGFALKVGHLLLVLCCWVVSVVYLNWFISSGIMETKMMGGGGGGGGKMWHKKWWENISGQGCKIHQQYYQYIGSKKVKRALWRKILLTWVVGWFIVSLWIFSYMSLQGTEKRKETLASMCDERARMLQDQFNVSMNHIQAMSILISTFHHAKSPSAIDQKTFAKYTERTAFERPLTSGVAYAVRVLHSEREQFEKQQGWTIKRMDTLEQNPVHKDDYAPEALEPSPVQEEYAPVIFAQDTIAHVISVNVLSGKEDRENVLRARESGKGVLTAPFRLLKTNRLGVILTFAVYKRDLPSNATPNERIQATDGYLGGVFDVESLVEKLLQQLASKQSVIVNVYDTTNHTHPIAMYGSNESGDVFFHVSTLNFGDPFRKHEMHCRFKQKPPWPWVAITTSIGILVIALLVGHIFHATVNRIAEVEDDYRKEMELKKQAEAADVAKSQFLATVSHEIRTPMNGVLGMLHMLMDTDLDVTQQEYVRTAQESGKALVSLINEVLDQAKIEFGKLELEAVLFDIRAILDDVLSLFSEKSQGKGVELAVYVSDHVPELLIGDPGRFRQIITNLMGNSIKFTDKGHIFVTIHLVEEVVRSIEVDKESNSENTLSGSPVADSRRSWEGFKAFSQEGPLGSFSSPSSDLVNLIVSVEDTGEGIPLESQPLIYTPFMQVGPSISRKHGGTGIGLSISKCLVGLMNGEIGFVSIPKTGSTFTFTAVFTNGHCSSNECKVQQINNQPHSASSEFEGMTALIIDPRSVRAKVSRYHIQRLGIHVEMVSDLKQGLSTISNGNIIINMVLIEQEVWDRDLGLSSHFVNNTRRIDQGVPPKLFILVNSSSSFKASVNLGVHNPIVITKPLRASMLAASLQRAMGVQNKGAPHRELQSLSLRHLLRGRKILIVDDNSVNRAVAAGALKKYGADVVCVSSGKDAISSLKPPHQFDACFMDIQMPEMDGFEATKRVREMEDSVNREVSMDDFENITNWHVPILAMTADVIHATHEECLKWGMDGYVSKPFEAEQLYREVSRFFQSS

**CYTOKININ RESPONSE FACTOR 7 (CRF7)**

**>AT1G22985 (AtCRF7)**

MKRIVRISFTDMEATDSSSSEDESPPSSRRRGKKLVKEIVIDHSDPPEVGKTRFKIRIPASLLAARNTTANKKKFRGVRQRPWGKWAAEIRCGRVKGRPERIWLGTFETAEEAALAYDNAAIQLIGPDAPTNFGRPDVDSAVVKKQDSDASGGASEEVV

**>BnaA02g35550D**

MKRIVRISVTDAEATDSSSDEDTEEPCRETTTTAQVRRRRGKRLVKEIVIDPPDSDDNRDACKTRFKIRIPAEFLKATAEGGKKKFRGVRQRPWGKWAAEIRCGRALKGRRDRLWLGTFDTAEEAALAYDNAAVQLIGPHAPTNFSFPAESQEVKMVAGASAVARVA

**>BnaAnng35170D**

MKRIVRISFTDVEATDSSSSEDDQTNTESPSPRKGKRFVKEIVIDPSDSAEVRKTRFKIRIPARLTKKFRGVRQRPWGKWAAEIRCGKAHGGIRNGGPVRLWLGTFETAEEAALAYDKAAIRLIGPHAPINFGPESPAVKQDSVAGD

**>BnaA08g20700D**

MKRIVRISFTDAEATDSSSSEDDEQTNTESPPPPRRRGKRCVNEIVIDPTDSSGKKNRDRGGKTRFKIRIPARLEKKNKKFRGVRQRPWGKWAAEIRCGKARDGRPVRVWLGTFETAEEAALAYDKAAVQLIGPDAVTNFGCSKAAKEGSDAGTSAVA

**>Csa_6G133770**

MSVGDMASLSGHKTNCRRKALTSGESTKPNQRLLRIIVTDADATDSSSEDELILGSRTAIRRQVREITIKRYSVPDSSSPKSPVSEICKKRNPRSRRSNNSCRRNKFRGVRQRPWGRWAAEVRDPILRKRIWLGTFDTAEEAAAVYDRAAIELQGPNAATNFSGDGAVKSAVEGSSKEEEEEEEEEEGGVESRKTTAAWSPTSVLHYDSFLTPIEEMGYCGEVDELGLEIGAASLPTARRQYGGEEELGEIELDLDYFLVDVIY

**>GLYMA_02G236800**

MSVHSKLKHHPMNLATPSSHFDNDPPCKTKTKTQRRLLRIIVTDHDATDSDSDEEEEQQQQTTRVKREITQINMQLPLSYNSSFPSSSSYYSSASACTSSEQNLKCKRPNKKPPPFSAVVRRRNNKFRGVRQRPWGRWAAEIRDPTRRKRLWLGTFDTAEEAATEYDRAAVKLKGPNAVTNFPLTPEAAAEAPVVSVDNLSSDGGASYSDLVASPTSVLAYECDSTPFDGFRYLDVEAFEFHIDAPLSLPDVNVALTCHHGKKEEEVAFDEFDIDEFMTWPY

**>GLYMA_18G051500**

MSDTHQTFMSVQPPLNLHHPFNPQASSSNFDGDPPCKTAAKTKPPRKKLLRVILTDHDATDSDSSGDDDDPKNPPKHKKVKRQITHITINLPSFSEAPTPAPIPTPSSSSSIDPTRLTLPKKRPAVPRRRSKFRGVRQRPWGRWTAEIRDPNQRKRVWLGTFDTAEEAAAVYDDAALKLKGPNAVTNFPLSAAGKTEHDTAPPEAAFSGEGFSSPTSVLTYFDGDSTPLYGFRYGEVDAFGFDIDAPFSLTDVNLGVLSQRFGKEDFGEFDPDEFLTWPS

**>GLYMA_11G199300**

MSDTHQPFMSVQPPMKLHNHPFNPQASSSNFDGDPPCKTAGKTKPPHTKLLRVILTDHDATDSDSSGDDEDPKNTKKHKKVKRQITHITINLPFFSKSPTPTSTPSSSSLDPTRSTRPKKRLGVPRRRNKFRGVRQRPWGRWTAEIRDPTQRKRVWLGTFDTAEEAAAVYDEAAVKLKGPNAVTNFPLSAAGNTEHDTPPPETVFSGEGLSSPTSVLTYCDGDSTPFDGFRYGEVDAFGFEFDIDAPFSLTAVNLGVLSQRFGKEEFGEFDPNEFLTWPS

**>GLYMA_14G205600**

MSVHAKLKHHPMNLASPSSHFEDDPPCKTKTKTQRRLLRIIITDHDATDSDSSDEEQQQQQQKTRRVNREITQINMQLPLSHNSSFSPSSSYYSSASTSSEQNLKCKRPNKKPPPSSAEARRRNKFRGVRQRPWGRWAAEIRDPTRRKRLWLGTFDTAEEAATEYDRAAVKLKGPNAVTNFPLAPEATAQSPPLAADNLSSDGGASYSDLVASPTSVLAYECDSTPFDGFRYLDVDAFGFHIDAPLSLPEVNVNVALTCHHGKKQEEAFDEFDLDEFMTWPY

**ARABIDOPSIS RESPONSE REGULATOR 1 (ARR1), ARR10 and ARR12**

**>AT3G16857 (AtRR1)**

MMNPSHGRGLGSAGGSSSGRNQGGGGETVVEMFPSGLRVLVVDDDPTCLMILERMLRTCLYEVTKCNRAEMALSLLRKNKHGFDIVISDVHMPDMDGFKLLEHVGLEMDLPVIMMSADDSKSVVLKGVTHGAVDYLIKPVRMEALKNIWQHVVRKRRSEWSVPEHSGSIEETGERQQQQHRGGGGGAAVSGGEDAVDDNSSSVNEGNNWRSSSRKRKDEEGEEQGDDKDEDASNLKKPRVVWSVELHQQFVAAVNQLGVEKAVPKKILELMNVPGLTRENVASHLQKYRIYLRRLGGVSQHQGNLNNSFMTGQDASFGPLSTLNGFDLQALAVTGQLPAQSLAQLQAAGLGRPAMVSKSGLPVSSIVDERSIFSFDNTKTRFGEGLGHHGQQPQQQPQMNLLHGVPTGLQQQLPMGNRMSIQQQIAAVRAGNSVQNNGMLMPLAGQQSLPRGPPPMLTSSQSSIRQPMLSNRISERSGFSGRNNIPESSRVLPTSYTNLTTQHSSSSMPYNNFQPELPVNSFPLASAPGISVPVRKATSYQEEVNSSEAGFTTPSYDMFTTRQNDWDLRNIGIAFDSHQDSESAAFSASEAYSSSSMSRHNTTVAATEHGRNHQQPPSGMVQHHQVYADGNGGSVRVKSERVATDTATMAFHEQYSNQEDLMSALLKQEGIAPVDGEFDFDAYSIDNIPV

**>AT4G31920 (AtRR10)**

MTMEQEIEVLDQFPVGMRVLAVDDDQTCLRILQTLLQRCQYHVTTTNQAQTALELLRENKNKFDLVISDVDMPDMDGFKLLELVGLEMDLPVIMLSAHSDPKYVMKGVKHGACDYLLKPVRIEELKNIWQHVVRKSKLKKNKSNVSNGSGNCDKANRKRKEQYEEEEEEERGNDNDDPTAQKKPRVLWTHELHNKFLAAVDHLGVERAVPKKILDLMNVDKLTRENVASHLQKFRVALKKVSDDAIQQANRAAIDSHFMQMNSQKGLGGFYHHHRGIPVGSGQFHGGTTMMRHYSSNRNLGRLNSLGAGMFQPVSSSFPRNHNDGGNILQGLPLEELQINNNINRAFPSFTSQQNSPMVAPSNLLLLEGNPQSSSLPSNPGFSPHFEISKRLEHWSNAALSTNIPQSDVHSKPDTLEWNAFCDSASPLVNPNLDTNPASLCRNTGFGSTNAAQTDFFYPLQMNQQPANNSGPVTEAQLFRSSNPNEGLLMGQQKLQSGLMASDAGSLDDIVNSLMTQEQSQSDFSEGDWDLDGLAHSEHAYEKLHFPFSLSA

**>AT2G25180 (AtRR12)**

MTVEQNLEALDQFPVGMRVLAVDDDQTCLKILESLLRHCQYHVTTTNQAQKALELLRENKNKFDLVISDVDMPDMDGFKLLELVGLEMDLPVIMLSAHSDPKYVMKGVTHGACDYLLKPVRIEELKNIWQHVVRSRFDKNRGSNNNGDKRDGSGNEGVGNSDQNNGKGNRKRKDQYNEDEDEDRDDNDDSCAQKKQRVVWTVELHKKFVAAVNQLGYEKAMPKKILDLMNVEKLTRENVASHLQKFRLYLKRISGVANQQAIMANSELHFMQMNGLDGFHHRPIPVGSGQYHGGAPAMRSFPPNGILGRLNTPSGIGVRSLSSPPAGMFLQNQTDIGKFHHVSSLPLNHSDGGNILQGLPMPLEFDQLQTNNNKSRNMNSNKSIAGTSMAFPSFSTQQNSLISAPNNNVVVLEGHPQATPPGFPGHQINKRLEHWSNAVSSSTHPPPPAHNSNSINHQFDVSPLPHSRPDPLEWNNVSSSYSIPFCDSANTLSSPALDTTNPRAFCRNTDFDSNTNVQPGVFYGPSTDAMALLSSSNPKEGFVVGQQKLQSGGFMVADAGSLDDIVNSTMKQEQSQGDLSGGDLGYGGFSSLRTCI

**>BnaA03g34320D**

MMNRGLGSGGGSSSGKNQGGEAVVEMFPSGLRVLVVDDDPTCLMILERMLRTCLYEVTKCNRAEMALSLLRKNKHGFDIVISDVHMPDMDGFKLLEHVGLEMDLPVIMMSADDSKSVVLKGVTHGAVDYLIKPVRMEALKNIWQHVVRKRRTEWSGVPTHSGSREAVSRGEEDGADDNNSSSEGGEEQGDEDASNLKKPRVVWSVELHQQFVAAVNQLGVEKAVPKKILELMNVPGLTRENVASHLQKYRIYLRRLGGVSQHQGSLNNSFMTSQDASFGSLPTLNGFDLQALAQLPAQSLAQLQAAGLGRPAAMNSKPGLHVSSSIVDERSVFSFDNPKMNLLHGVPTGMEPRQLAGLQQHRMTIQQQIAAVRAGHSLQNNGMRMPLASQPQQPFSRPQQSSIRQPMLPNRSGFSGRSSIPESSRVLPTTSYTNLATQQQHSMAFSNFQQELPLNSFPLASAPGLSVRKPHSSSYREEVNSSEAGFTTPSYDMFSSRQNDWDLRSMLSPHQDSQAYSSSSMSRNNNSAVAATDHSRNHHQQTPQGMVSHHQVYGNGGGSSVKVKSETMGFHEQYSNQEDLMSALLKQEGIGPVDTEFDFDAYSIDDIPV

**>BnaC05g36490D**

MMNPSQGRGLGSGGGSSSGRNKGGEAVVEMFPSGLRVLVVDDDPTCLMILERMLRTCLYEVTKCNRAEMALSLLRKNKHGFDIVISDVHMPDMDGFKLLEHVGLEMDLPVIMMSADDSKSVVLKGVTHGAVDYLIKPVRMEALKNIWQHVVRKRRSEWSVPEHSGSIEETGQQQQQRGPAVSEDAADDNASSVNNEGNNWRSSSNNSRKRKEEEGDEQGDEDASNLKKPRVVWSVELHQQFVAAVNQLGVEKAVPKKILELMNVPGLTRENVASHLQKYRIYLRRLGGVSQHQGNLNNSFMTGQDASFGPLSSLNGFDLQALAVTGQLPAQSLAQLQAAGLGRPAMVSKSGLPRSGFSGRSSVAESSRVLPTSYTNLATQQHSSTSVAFNSFQQELPVNSFPLPSAPGLSVPTQVRKPHSSSSSYQEEQLTQPIMVPHHQVYSNGGGGGSSVRVKSERDTAAMAFHEQYSNQEDLMSALLKQEGVAPVVDTEFDFDAYSIDDIPV

**>BnaA03g42350D**

MLTPGAVGGSSNSDPFPSGLRVLVVDDDPTCLMILERMLKTCLYRVTKCNRAEIALTLLRKNKNGFDIVISDVHMPDMNGFKLLEHVGLEMDLPVIMMSADDSKSVVLKGVTHGAVDYLIKPVRIEALKNIWQHVVRKKQNVSEHSGSVEETGGDRQQQQQRDDDDDGGGDNNSSSGNNEGNLRKRKEEEQGDDKEDTSSLKKPRVVWSVELHQQFVAAVNHLGVDKAVPKKILEMMNVQGLTRENVASHLQKYRIYLKRLGGVSQGNMNHSFMTGQDPSYGPLNGFDLQGLATAGQLQAQSLAQLQAVGLGQSSSPLIKPGITSVDQRSFKQLASRFGDGHGPMMMNGGGGNKQTSLLHGVPTGHMRLQQQQMAGMRVAGPSMQQQQQQSMLSRRSVPETRSSRVLPAATHSAFNNSFPLASAPGMMSVSDTKGVNEFCNPSYDILNNFPQQQHHNNNNSVNEWDVRNVGMVFNSHQDNTTSAAFSTSEAYSSSSTHKRKREAELVVEHGQNQQQPQSRSVNPMNQIYMNDGGSVRMKTETVTCPPQATTMFHEQYSNQDDLLSDLLKQEGLLDTEFDFEGYSFDNILV

**>BnaC07g43590D**

MTVEQDFEAVDQFPVGMRVLAVDDDQTCLRILETLLHRCQYHVTTTDSAQTALELLRENKNKFDLVISDVDMPDMDGFKLLELVGLEMDLPVIMLSAHSDPKYVMKGVKHGACDYLLKPVRIEELKNIWQHVVRKSKFKKMKSIVIHDESSQGNSDQNGVKANRKRKDQFEEVEEEDEERGNENDDPTAQKKPRVLWTRELHNKFLAAVDHLGVEKAQPKKILDLMNVDKLTRENVASHLQKFRSALKKITNEANQQASMAAIDSHFMQMSSLKGLGGFHHQRQIPLGSSQFHGGAATMRHYPLGRLNSFGGVFPHVSSSLPRNHNDGGGYILQGMPIPPLDDLHINNNNKAFPGFSSQQSSLMVAPNNQLVLQGHQQSSYPSLNPGLSPHFEINKRLEDWSNALLSTNIPQSDVHSKPDALEWNHFCNSDAAQAGFIDPLQMKQQPANNLGPMTDAQRLRSSNPNEGLFVGQQKVENGSMPSNAGSLDDIVNSMMPKEQSQAEFFEGDLGFGWHNSSLRTCI

**>BnaA03g51830D**

MTVEQDFEAVDQFPVGMRVLAVDDDQTCLRILETLLHRCQYHVTTTDSAQTALELLRENKNKFDLVISDVDMPDMDGFKLLELVGLEMDLPVIMLSAHSDPKYVMKGVKHGACDYLLKPVRIEELKNIWQHVVRKSKFKKMKSIVINDDHSQGNSDQNGVKANRKRKDQFEEVEEEDEERGNENDDPTAQKKPRVLWTRELHNKFLAAVDHLGVEKAQPKKILELMNVDKLTRENVASHLQKFRSALKKITNEANQQANMAAIDSHFMQMSALKGLGGFHNQRQIPLGSGQFHGGAATMRHYPLGRLNSFGGVFPHVSSSLPRNHNDGGYVLQGMPIPPLDDLNNKAFPSFTSQQSSLMVAPNNQLVLQGHQQSSYPSLNPGLSPHFEINKRLDDWSNALLSTNIPQSGVHSKPDALEWNHFCNSDAAQAGFIDPLQMKQQPANNLGPMTDAQLLRSSNPIEGLFVGQQKLENGSMPSNAGSLDDIVNSMTPKEQSQAELFEGDLGFGWHNSSLRTCI

**>BnaC01g06500D**

MTVEQGIEAVDQFPVGMRVLAVDDDHTCLRILETLLHRCQYHVTTTDKAQTALELLRENKNKFDLVISDVDMPDMDGFKLLELVGLEMNLPVIMLSAHSDPKYVMKGVKHGACDYLLKPVRIEELKNIWQHVVRKSKLKKMKGIVTNGESEGNSDQNGVRANRKCKDQLEEKDEEEQEQERGNDKKPRVIWTRELHNKFLAAVDHVGFKNAVPKKILDLMNVDNLTRENVASHLQKFRLALKKITNEAKQQANMAAANSHFMQMSSLKGFGSFHHSPIPLGSSQFHGGAATMRHYPSNRTFAGMFPHVSSSLPRNLHNDGGHMLQGTPFPPLENLHINSNNKAFPSFASQQSSLLVAPSMVLEGHQQSTSPSFSPHLEINKRLEDWSNNIPQSNVHSEPDALGWNMFGNSTNQTYFCRDTDFGSSSAAHADSLQMKQQHENIFSPMTVAQLLRSSSANEGLFMGQQKLEDGFMASDAGSLDDIVNSMMTQFFECSRNSQAEFSEGDWVWVA

**>BnaA04g14760D**

MTVEQQDCVALDQFPVGMRVLAVDDDQTCLRILESLLHRCQYHVTTTNQAQKALELLRENKNKFDLVISDVDMPDMDGFKLLELVGLEMDLPVIMLSAHSDPKYVMKGVTHGACDYLLKPVRIEELKNIWQHVVRKNRGSNNGDKKDGSGNEGVANSDQNNGRANRKRKDQYNEDEDEERDDNDDPSSQKKPRVVWTVELHKKFVAAVNQLGFEKAMPKKILDLMNVEKLTRENVASHLQKFRLYLKRISGNQQAIMANSDLHFLQMSNGLDGFHHRPIPVGTGQFHGGAAAAGMRPFPPNGILGRLNTPSGMSGVRNLSSSPSSGMFLQNPTDLGKFHHVSSLPLNHIDGGNILQGLPMPLEFDQLQTNNNKSIIAGNSMAFPSFPTQQQSSLPNNNNHLVLEGHPQAPPSAFPGHQINKRLEHWSNAVSSSSSTLPPPGQNSNSLISHQFDASSSSYSIPFCDSTIPLNPALDHTNPRAFYRATDMDSSANVQPGVYYDSLQMRKSGNYGPTTDAMLSSNNPKEGFTVGQQKLQSGFMGGEAGSLDDIVNSTMKQEQSQGDLSEGDLGYGGFSSLRTCI

**>BnaC04g56320D**

MTVERQDCVALDQFPVGMRVLAVDDDQTCLRILESLLHRCQYHVTTTNQAQKALELLRENKNKFDLVISDVDMPDMDGFKLLELVGLEMDLPVIMLSAHSDPKYVMKGVTHGACDYLLKPVRIEELKNIWQHVVRKNRGSNNGDKKDGSGNEGVANSDQNNGRANRKRKDQYNEDEDEERDDNDDPSSQKKPRVVWTVELHKKFVAAVNQLGFEKAMPKKILDLMNVEKLTRENVASHLQKFRLYLKRISGNQQAIMANSDLHFLQMSNGLDGFHHRPIPVGSGQFHGGAAAAGMRPFPPNNGILGRLNTPSGIGGVRNLSSPPSSGMFLQNPTDLGKFHHVSSLPLNHIDGGNILQGLPIPLEFDQLQTNNNKSIIAGNSMAFPSFPTSLPNNNNHLVLEGHPQASPSAFPGHQINKRLEHWSNAVSSSSSTLPPPGQNSNSLISHQFDASSSNYSIPFCDSTIPLNPALDHTNPRAFYRATDLDSNANVQPGVYYDSLQMRQSGNYGPTTDAMLSSNPKEGFTVGQQKLQSGFMGGEAGSLDDIVNSTMKQEQSQGDLSEGDLGYGGFSSLRTCI

**>BnaC03g39750D**

MMNPLHGRGGLGSGGGSSSGKNQGGEAVVEMFPSGLRVLVVDDDPTCLMILERMLRTCLYEVTKCNRAEMALSLLRKNKHGFDIVISDVHMPDMDGFKLLEHVGLEMDLPVIMMSADDSKSVVLKGVTHGAVDYLIKPVRMEALKNIWQHVVRKRRTEWSGVPTHSGSQQREAVSSGEDGADDNNSSSEGGEEQGDEDASNLKKPRVVWSVELHQQFVAAVNQLGVEKAVPKKILELMNVPGLTRENVASHLQKYRIYLRRLGGVSQHQGSLNNSFMTSQDASFGSPPTLNGFDLQALAQLPAQSLAQLQADASQQQQPFPRPQQSSIRQPMLPNRKQQKLPVNSFPLASAPGLSVRKPHSSSYREEVNSSEAATDHSRNHYQQTPPGMVSHHQVYRNGGGSSVKVKSETMGFHEQYSNQEDLMSALLKQEGIGPVDTEFDFDAYSIDDIPV

**>BnaA03g34300D**

MFSSGLRVLVVDDDPTCLAILERMLRTCSYEVTKCNRAEMALSLLRKNKHGFDIVISDVHMPDMDGFELLEHVGLQMDLPVISTVVLRGVTHGAVDYLIKPVRIEALQNIWQHVVRKRRSVPEHSDGEDAADDNSSSVNGGKKWRSML

**>Csa_3G165610**

MSTASSSSVRKAGEAVPDQFPAGLRVLVVDDDPTCLMILEKMLRICRYDVTNCSRAEDALSLLRQNKNGFDIVLSDVHMPDMDGFKLLEYIGLEMDLPVIMMSVDDGKNVVMKGVTHGACDYLIKPVRMEALKNIWQHVVRKRKNEWKDLEQTCVDDVDRQQKTNEDADYSSSANEGSWRNSKRRKDDVEDPEERDDSSTLKKPRVVWSVELHQQFVAAVNQLGIDKAVPKKILELMNVPGLTRENVASHLQKYRLYLRRLSGITQHQSNLNNTFMSAQDAFGPPLNGLDLQTLAAAGQLQPQSLATLQAAGFGRSTAKSGMPMPLVDQRNHIFSFENPKLRFGDGQQPHLNGSKPMNLLHGIPTTMEPKQLANLQHSAQSHGNMTMQVSIQGGQSSSQLMQTPQPQARAQILNESTTTSVTRLPQTMQPSILPNGTTSAVLARTEFGNNNRGGGYNLVSPASTMLNFPLNQTAELPGNSFPLQSTPGMSSIVPKGRFPDDVNSDIKGSEGFGPSYDMFRDLHPQKPHDWDLQHVGVTFDTSQGSLDIPPSAFSHQGYASSQQNGQNRNTSTAGKAMFLLEEGSDNGNAQSMGQQLNPIFVDGSVRVKSERASDISSQTDLFSEPFGQEDLMSSLFKQQQGSIATAESELEWLFHHNTPV

**>Csa_6G312560**

MDLGHMKGSIPTPTPTPTSISARKATDMSSDQFPAGLRVLVVDDDPTCLKILEKMLQNCCYAVTKCNRAEIALSMLRENKNGFDIVLSDVHMPDMDGFKLLEQVGLEMDLPVIMMSADDGKNVVMKGVIHGACDYLIKPVRIEALRNLWQHVVRKRKTEWKDLEQSGSVDEGDREQNLSEGANYSSSAYEGSWNSSKRRRDAEEEPEERDDTSTLKKPRVVWSVELHQQFVAAVNQLGIDKAVPKKILELMNVPGLTRENVASHLQKYRLYLRRLSGVSPHQSNLNNSFINPQDPPFGSMGSFNGIDLQTLSVTGQLSPQSLAALQATGLRRPTAKSGISMALDQNNIFSFENPKLRFVEDQTQHLNNSKPVNLFHGIPTKMEPKQLANIQHPSVQPQGNINMQLNIKSEHGGTQLMHLSQQQTIGQTLGNSPASHLPRISSTLRKPIISGSVMRGNGTADNSHRPGYNLVSPTSVMVNYPMSQTTELSNNYSLQSNSSVSTLNCKGMFQEEISSDLKVSGGLMSSYDVFSDLPLQKSHDWDSQNVSTLAFGTFHHGNFIQDSFDISSSTLIHHGFPSSQTNGQNQNSSVVGKTVFSFSDSTQPENPQNIFQNSLLDPVEVKSQRIPDANYQTDLLSEDFGQDELLGVFIKQQQQGDNGSVDFSGYQTENICV

**>Csa_4G652100**

MTVESRLDDPVDQFPTGMRVLAVDDDPTCLLILETLLRRCQYHVTTTNQAVMALKLLRENKNKFDVVISDVHMPDMDGFKLLELVGLEMDLPVIMLSANGDPKLVMKGITHGACDYLLKPVRIEELKNIWQHVIRRKKFDPKDRMNSGNQDRPDSENGEESADPNGKFNKKRKDQNYNEDDDQDYGQDNDDSSTQKKPRVVWSVELHRKFVNAVNQLGIDKAVPKKILDLMNVEKLTRENVASHLQKYRLYLKRISCVANQQANMVTALSSADPSYLRIGSLGGIGNFHSLTGPPQFPNTSYRSIPTTGMFGRLNSPACMGMHGLSSPGTVQLGNLSNSSNDQSTFQPIVLSGNRSGNIFRGMPEPSEIDQLQHVKHGTHVDNLSSAFDERTVFPSIGLPNGNMSSSTLSNPLLGLTNDPSIPEAQHHEVNGGRKFGTQSSISVVSSNSDSPRPSLDNVRCTGDNWSNAVHSSRVHLNTIPSANLSNVATSYHGQLRDNTTSALHIGNCLSDISSITSLSTQSHESRIDSQRQAATTSNRFDQLISKVPVQDWNDHKEDAYYHTTMACSSSNSLIPVNGAAETLSKRLENPNAIFSGSKDFNATGQANVLDHAMVEHDQFKKSNMETPLMLKQGYLYDHQQKMQSRFSSSNCGSLEDIACAMMKQEQDDGKLLEGEFGCDNYPIGTCM

**>GLYMA_07G243300**

MNLSNGKGSMSTLTASVVMKSGDAVSDQFPAGLRVLVVDDDPTCLMILEKMLRTCLYEVTKCNRAETALSLLRENKNGFDIVISDVHMPDMDGFKLLEHIGLEMDLPVIMMSADDGKSVVMKGVTHGACDYLIKPVRIEALKNIWQHVVRKRKNEWKDAEQSGSAEEGDRQPKASDEADYSSSANEGSWRNSKKRRDEEEEAEDRDDTSTLKKPRVVWSVELHQQFVAAVDQLGIDKAVPKKILELMNVPGLTRENVASHLQKYRLYLRRLSGVSQHQNNMNNSFLSPQEATFGTISSINGIDLQTLAVAGQLPAQSLATLQAAGLGRPTGKAGVPMPLMDQRNLFSFENPRLRFGEGQQQHLSTSKPMNLLHGIPTNMEPKQLANLHQSTQSIGSLNMRVNASATQGSPLLMQMAQSQPRGQMLSENIGPRVPRLPSSLGQPTVSNGISNGLLGRNGIAGNNRGPAYNPVPPSSSLLSFPMNQTSEMSVNNSFPLGSTPGISSITTKGSFQEEVTSGIKGSGGFPSYDIFNELHHQKPHDWEITNPNLTYNASQHANPLQGNIDVTPSVLVHQGFSSTQQTGQSRDAALIGKAMFSMGEGLEQNNFQNASQNLNSLLLDNSIRVKAERIPDASSQTNLFPEHYGQEDLMSALLKQQEGMGPSENEFDFDGYSLDNIPV

**>GLYMA_17G030600**

MNLSNGKGSMSTLTASVVMKSGDAVSDQFPAGLRVLVVDDDPTCLMILEKMLRTCLYEVTKCNRAETALSLLRENKNGFDIVISDVHMPDMDGFKLLEHIGLEMDLPVIMMSADDGKSVVMKGVTHGACDYLIKPVRIEALKNIWQHVVRKRKNEWKDAEQSGSAEEGDRHPKASDEADYSSSANEGSWRNSKKRRDEEEEAEDRDDTSTLKKPRVVWSVELHQQFVAAVDQLGIDKAVPKKILELMNVPGLTRENVASHLQKYRLYLRRLSGVSQHQNNLNNSFLGPQEATFGTISSINGIDLQTLAVAGQLPAQSLATLQAAGLGRSTAKAGVPIPLMDQRNLFSFENPRLRFGEGQPQHLSTSKPMNLLHGIPTNMEPKQLANLHQSTQSIGGLNMRVNASATQGNPILMQMAQSQPRGQMLSENTGPRVPRLPSSLGQPTVSNGISNGLLGRNGIAGNNRGPAYNPVPPSSSLLSFPMNQTSEMSVNNSFPIGTTPGISSITTKGSFQEEVTSGIKGSSGFPSYDIFNELHHQKSHDLEITNPGLTYNASHHVNPLQGNIDVAPSVLVHQGFSSTQQTGQSRDGALIGKAMFSMGEGLEQNNLQNANFNPLLVDNSIRIKAERIPDASSQTNLFPEHYGQEDLMTALLKQQEGMGPAENEFEFDGYSLDNIPV

**>GLYMA_15G145200**

MNLSNGKGSTSSSPLKAGDSVSDQFPAGLRVLVVDDDPTCLMILERMLRACLYEVTKCQRAEVALSLLRENKNGFDIVLSDVHMPDMDGFKLLEHIGLEMDLPVIMMSADDGKHVVMKGVTHGACDYLIKPVRIEALKNIWQHVIRKRKNGLRDVEQSGSVEEGDQPPKVSDDGDYSSSVNEARSSKKRRDEDEEGDEKDDSSTLKKPRVVWSVELHQQFMAAVNQLGIDKAVPKKILELMNVPGLTRENVASHLQKYRLYLRRLSGVSQQQGNLNNSFMSSQEATFGGTSINGIDLQTLSAAGQFPSQSLAKLQATGLGRTTAKAGVPMPLSDQKNLFSFENPRLRFGEGSLQHLSNSKPINLLHGIPTNMEPKQLANLHQSTQPLGSLNMRVNAPVTQNNPLLMQMAQSQPRGQMLGENAGSHVTRFPSSLVQPTVSNGISNGVLGNGIAGTSNITTYNPVQQNSSLLSFPMNQTNEISVSNFPLRSSPGITSITNKGMFHEEGTSGIKGPGGFVQGYDMFNELHHQKSHDWDLTNTGMTYDAAHHANPLQGNIDVSPSVLVHQSFPTMHQTVQNRDTTSIGKAMFSTGEGMHQSNLQNVGQHHNNLLLDNSVRVKAERIPDPSCQINNLFADQYGQEDLVSAFLKQQESIGTSENEFDFDGYSLDNIPV

**>GLYMA_09G040000**

MNLSHGKGSTSSSPLKAGDTVSDQFPAGLRVLVVDDDPTCLMILERMLRACLYEVTKCKRAEVALSLLRENKNGFDIVLSDVHMPDMDGFKLLEHIGLEMDLPVIMMSADDGKQVVMKGVTHGACDYLIKPVRIEALKNIWQHVVRMRKNGLRDVEQSGSMEEGDRPPKGSDDGNYSSSVNEAKSSKKRRDEDEEGDERDDSSTLKKPRVVWSVELHQQFMAVVNQLGIDKAVPKKILELMNVPGLTRENVASHLQKYRLYLRRLSGVSQQQGNLSNSFMSSQEATFGGTSINGIDLQTLSAAGQFPSQSLAKFQAAGLGRTTAKAGMPMSLSDQKNLFSFENPRLRFGEGSLQHLSNSKPINLLHGILTNMEPKQLANLHQSTQPLGSLNMRVNSPATQNKPLLMQMTQSQPRGQLLSENASSHVTRYPSSLVQPTVPNGISSGVLGNAIAGTSNITTTYNPVQQNSSLLSFPMNQTNEMSASNFHLRSTPGITSIPNKGMFHEEGTSGVKGSGGFVQGYDMFNDLHHQKSHDWDLTNTGMTYDASQHANPLQGNIDVSPSVLVHQSFPSMQQTVQNRDTTSIGKAMFSTGEGMHQSSLQNIGQHHNNLLLDNSVRVKAERIPDPSCQINNLFSDQYGQEDLVSAFLKQQEGVGTAENEFDFDGYSLDNIPV

**>GLYMA_14G110600**

MTVVEDHRMDDLRDQFPIGMRVLAVDDDSTCLMVLETLLRRCQYHVTTTKNAITALKLLRENKTMFDLVISDVHMPDMDGFKLLELVGLEMDLPVIMLSVNDDPKMVMKGITHGACDYLLKPVRIEELQNIWQHVIRRKKIDSKERNKTSDHDKPNADNGNGRVSAGTGNSDQNGKPSKKRKDQDEDDEEENEDGHDNEDSSTLKKPRVVWSVELHRKFVSAVNQLGIDKAVPKKILDLMNVEKLTRENVASHLQKYRLYLKRISCVANRQANLVAALGTADSSYLRMGSLSGVGHMQTLTGPQQFHNNAFRPFPPGGMIGRLNASVGLNMHGISSSEALQLSHAQNLNKSINDPLKFQSAIACGNQNGIQGMPMSIGLDQLQHNKGVSVGPIQNMSSLIDDRPTFGVSKKLSDQTQKVTIGCSPSPVLDISNNDLVLKADSENTQGGGVYENLTSVASQHSQFSLPLLDHGRCSDIWSSPMQSSGTNSYPPSETLQGGNLSGQSSITSLFNQSHDSPTDMHSQGLIFTNNLGQMSNNVPFQGWDDNNHDSNYDANVIGNSIDTLIDPEGHTSINSNYNRNLDFNFCNPLQMKHDGIMGLSEENSLKQQYGYIMNRQKSQNSSATNNLGSLEDFASSMMKQKQDKVKFLDGSYLCNNNYPAGTSM

**>GLYMA_04G062500**

MTVEKKRNDSGDEFPVGMRVLAVDDDPTCLLVLKTLLQRCQYHVTTTNQAIKALALLREHKDKFDLVISDVHMPDMDGFKLLELVGLEMDLPVIMLSANGDTKMVMKGISHGACDYLLKPVRMEELKNIWQHVIRRKKFDSKEKNKTSNLDKPTSNSGNGLGSSGTGNSDQNEKLTKKRKDQDEDEDEDQENDLDNEDPSAQKKPRVVWSVDLHRKFVAAVNQLGIDKAVPKKILDLMNVEKLTRENVASHLQKYRLYLKRISCVANQQANMVAALGVADPSYLRMSSVSGVGHVQTLSGSGQLHNNNFRSFPPSGIINRLNTPAGLNVHGFPSGVLQLSQSQNLNNANDHHKFQSVIVPVNQNGVQGMTVSVGLDQLQNNKVVMSVQNLTTVFDAKTTFPIPNKLPDPRPKITNSVSHTSDVHFSNNALMLEPRPQGSQGSVRIGTLSSSVASQHSEFSLSLLDQGRYSDNWTSAVQPSVIQTNFFPPSECFRQTNIPPADNMASVPLQGGNLSGPSITSLSRQSHDSMTEMHSEGMTFTNRPGHTSSNVPFQGWDDHNQDATHHSNIMSINSLTPVNGAAVQAGHAATNSVLHRNLDFNYCDPLQMKHEGFVELTDETLLKQHQGNTMDQQKSQENHFSNNLGSLEDLVSSMMK

**>GLYMA_06G063500**

MTVEKKMNDSGDEFPVGMRVLAVDDDPTCLLVLETLLRRCQYHATTTNQAIKALALLREHKDKFDLVISDVHMPDMDGFKLLELVGLEMDLPVIMLSANGDTKLVMKGISHGACDYLLKPVRMEELKNIWQHVIRRKKFDSKEKNKTRNIDKPTSNSSNGLGSSGTGNSDHNEKLTKKRKDQDEDEDEEQENDHDNDDPSAQKKPRVVWSVELHRKFVAAVNQLGIDKAVPKKILDLMNVEKLTRENVASHLQKYRLYLKRISCVANQQTNMVVALGGADPSYLRMNSVSGVGHIQSISGSGQLHNNAFRSFPPSGIINRLNTPAGLNVHGFPSGVLQLSQSQNLKNTNDNLKFQSAIVPANQNGVHGMTVSVGLDQLQNNKGVMSVQNLTTVFDAKTTFPISNKLPDPRPKITNSGSHTPDVSFSNNALMLEPRPQGTQGSVRIETLSSSVASQHSEFSLSLLDQGRYSDNWASTVQPSVIQTNSYPPSECFGQTNIPPTDNMASVPLQGGNLSGASITSLSRQSYDSMTDMHSEGVTFTNRPGHISSNVPYQGWHDNNQDATHHSNILSINSLTPVNGAAVPAGHAAMNSALHRNLDFNYCDPLQMKHEGFVELTDEALSKQHQGNIMNLPKSQQSHFSNNLGSLEDLVSSMMKQENDKMKLLDGNLICNNYSGGLSRCDSRFSSTFQNIMHPR

**>GLYMA_17G217100**

MTVVENHTMDDLRDQFPIGMRVLAVDDDSTCLMVLETLLRRCQYHVTTTKNAITALNLLRENKTMFDLVISDVHMPDMDGFKLLELVGLEMDLPVIMLSVNDDPKMVMKGITHGACDYLLKPVRIEELQNIWQHVIRRKKIDSKEQNKTSDYDKTNSDSGNGRGSAATGNSDQNGKPSKKRKDQDEDDDEENDTDHDNEDPSTQKKPRVVWSVELHRKFVSAVNLLGIDKAVPKKILDLMNDEKLTRENVASHLQKYRLYLKRISCGANRQANMVAALGTADSSYLRMGSLSGVGHLQTLTGPQQFHNNAFRPFPPGGMIGRLNTSVGLNNHGLSSSEALQLSHAQNLNNSINDPLKFQSSIACGNQNAIQGMPMSIGLDQLQHNKGVSVSVGPIQNMSPLIDARPTFAVSNRLSDQIPKVTMGCSPSPVLDVSNNALVLKADSENTQGRGVYENLTSVASQHSQFSLPLLGHGRCSDIWSSPMRSSGTNSYPPSETLQGGNLSGASSITSLSNQSHDSQTDMHSQGLIFTNNSGQISNNVPFLGWDDHNHDSSYHSNVIGNSIDSLIDPEGHTSINSTYNRNLDFNFCDPLQMKHDGIMGLSDENSLKQQHRYIMNQQKSQNSRAPNNIGSLEDFVSSMMKQKQDKVKFLDGSYLCNNNYPDGTSM

**GIBBERELLIN-INSENSTIVE DWARF 1a (GID1a), GID1b, and GID1c**

**>AT3G05120 (AtGID1a)**

MAASDEVNLIESRTVVPLNTWVLISNFKVAYNILRRPDGTFNRHLAEYLDRKVTANANPVDGVFSFDVLIDRRINLLSRVYRPAYADQEQPPSILDLEKPVDGDIVPVILFFHGGSFAHSSANSAIYDTLCRRLVGLCKCVVVSVNYRRAPENPYPCAYDDGWIALNWVNSRSWLKSKKDSKVHIFLAGDSSGGNIAHNVALRAGESGIDVLGNILLNPMFGGNERTESEKSLDGKYFVTVRDRDWYWKAFLPEGEDREHPACNPFSPRGKSLEGVSFPKSLVVVAGLDLIRDWQLAYAEGLKKAGQEVKLMHLEKATVGFYLLPNNNHFHNVMDEISAFVNAEC

**>AT3G63010 (AtGID1b)**

MAGGNEVNLNECKRIVPLNTWVLISNFKLAYKVLRRPDGSFNRDLAEFLDRKVPANSFPLDGVFSFDHVDSTTNLLTRIYQPASLLHQTRHGTLELTKPLSTTEIVPVLIFFHGGSFTHSSANSAIYDTFCRRLVTICGVVVVSVDYRRSPEHRYPCAYDDGWNALNWVKSRVWLQSGKDSNVYVYLAGDSSGGNIAHNVAVRATNEGVKVLGNILLHPMFGGQERTQSEKTLDGKYFVTIQDRDWYWRAYLPEGEDRDHPACNPFGPRGQSLKGVNFPKSLVVVAGLDLVQDWQLAYVDGLKKTGLEVNLLYLKQATIGFYFLPNNDHFHCLMEELNKFVHSIEDSQSKSSPVLLTP

**>AT5G27320 (AtGID1c)**

MAGSEEVNLIESKTVVPLNTWVLISNFKLAYNLLRRPDGTFNRHLAEFLDRKVPANANPVNGVFSFDVIIDRQTNLLSRVYRPADAGTSPSITDLQNPVDGEIVPVIVFFHGGSFAHSSANSAIYDTLCRRLVGLCGAVVVSVNYRRAPENRYPCAYDDGWAVLKWVNSSSWLRSKKDSKVRIFLAGDSSGGNIVHNVAVRAVESRIDVLGNILLNPMFGGTERTESEKRLDGKYFVTVRDRDWYWRAFLPEGEDREHPACSPFGPRSKSLEGLSFPKSLVVVAGLDLIQDWQLKYAEGLKKAGQEVKLLYLEQATIGFYLLPNNNHFHTVMDEIAAFVNAECQ

**>BnaC05g46680D**

MAASDEVNLIDSKTVVPLNTWVLISTFKLSYNLLRRSDGTFNRHLAEYLDRKVTANANPEDGVFSFDVVIDRTTSLLSRVYRPAYASDQEGPVSVLDLEKPLDDEIVPVILFFHGGSFAHSSSNSKIYDTLCRRLVGACGGCVVVSVNYRRAPESPYPCAYDDGWTALNWVNSRTWLESKKDSKVRIFLAGDSSGGNIVHNVALKAGEAGIDVLGNVLLNPMFGGEERTESEKRLDGKYFVTVRDRDWYWKAFLPEGEDREHPACNPFGARAKSLRGLRFPKSLVVVAGLDLIQDWQLAYAEGLERAGKEVRLVHLEKATIGFYLLPNNSYFHSVMDEIAAFVNAES

**>BnaCnng69280D**

MAAGNEVNLNECKRIVPLNTWVLISNFKLAYTLLRRPDGSFNRHLAEFLDRKVPPNSFPLDGVFSFDHLDSSTNLLTRIYLPAPLDPSRYGAVDLTEPLSTTEIVPVLVFFHGGSFTHSSVNSAIYDTLCRRLVTICGVVVVSVDYRRSPEHRYSCAYDDGWNALKWVKSRIWLRSGKDSDVYVYLAGDSSGGNIAHNVVVRATNEGVKVLGNILLHPMFGGVERTQSEKRLDGKYFVTVQDRDWYWRAFLPQGEDRDHPACNPFGPRGRCLEGVKFPKSLVVVAGLDLVQDWQLAYVDGLKKSGQDVNLLYLKQATIGFYFLPNNDHFRCLMDELKKFVHSIEDDSLSKSSPILLTP

**>BnaC04g21040D**

MIIIMDSLRIVPLNTWVLISNFKLAYTLLRRPDGSFNRHLAEFLDRKVPSNSFPLDGVFSFDHLDSTSNLLTRIYLPAPLDPSRYGAVDLTEPLSTPEIVPVFIFFHGGSFTHSSANSAIYDTFCRRLVTICGVVVVSVDYRRSPEHRYPCAYDDGWNALKWVKSRVWLQSGKDSNVYVYLAGDSSGGNIAHNVAVRATNEGVKVLGNILLHPMFGGVERTQSEKRLDGKYFVTVQDRDWYWRAYLPEGEDRDHPACNPFGPRGQSLEGVTFPKSLVVVAGLDLVQDWQLAYVDGLRKTGHDVNLLYLKQATIGFYFLPNNDHFHCLMDELKKFVHSIEEDSQKQAKSYSSESIAT

**>Csa_1G572450**

MVVPLNTWVLISNFKLAYNLLRRPDGTFNRHLAEFLDRKVPANANPVDGAFSFDVIIDRATSLLCRIYRPANGGEPQTTNIVDLEKPVDSEVVVPVIVFFHGGSFAHSSANSAIYDTLCRRLVSLCKAVVVSVNYRRAPENRYPCAYDDGWAALNWVNSRSWLQSKDSKTYIYLAGDSSGGNIVHHVASRAVKSGIEVLGNILLNPMFGGQERTKSEVRLDGKYFVTIRDRDWYWRAFLPEGEDRDHPACNPFGPRGYSLEGIKFPKSLVVVAGLDLVQDWQLAYARGLENDGQEVKLLYLEQATIGFYLLPNTEHFYTVMDEISEFVSSDC

**>Csa_7G391240**

MAGSNEVNLNESKRVVPLNTWVLISNFKLAYTILRRADGTFNRELAEYLERKVPANVFPVDGVFSFDHVDRASGLLNRVYQLAPENEAKWGIIDLEKPLSTTKVVPVILFFHGGSFAHSSANSAIYDTFCRRIVSVCKAVVVSVNYRRSPEHRYPCAYEDGWAALKWVKSKTWLQSGKDSKVHVYLAGDSSGGNIAHHVAVRAAEEDIEVLGNILLHPMFGGEKRTESEKKLDGKYFVTIQDRDWYWRAYLPEGEDRDHPACNIFGPKAKSLVGLDFPKSLVVVAGLDLMQDWQLAYVQGLKDSGHNVKLLFLEQATIGFYFLPNNEHFYCLMEEINNFLNP

**>GLYMA_10G158000**

MAGSNELNPNDSKMVVPLNMWVLISNFKLAYNLLRRPDGTFNRDLAEFLDRKVPANANPVDRVFSFDVVVDRETNLLTRIYRPTEGEERSVNILDLEKPVSSEVVPVIIFFHGGSFAHSSANSAIYDTLCRRLVGICKAVVVSVNYRRAPENRYPCAYDDGWTALKWVSSRSWLQSKKDKKVHIYLAGDSSGGNIVHHVALKAVESGIEVFGNILLNPLFGGQERTESEKRLDGRYFVRVKDRDWYWRAFLPEGEDRDHHACNPFGPKGKSLEGITFPKSLVVVAGLDLVQDWQLGYAKGLEKAGQEVKLIFLEQATIGFYLLPNNEHFSPVMDEIKYFVSSDC

**>GLYMA_20G230600**

MAGSNELNPNDSKMVVPLNMWVLISNFKLAYNLLRRPDGTFNRDLAEFLDRKVPANANPVDGVFSFDVIVDRETNLLTRIYRLAEGEERSVNILDLEKPVNSEVVPVIIFFHGGSFAHSSANSAIYDTLCRRLVGICKAVVVSVNYRRAPENRYPCAYDDGWTALKWVSSASWLQSRKDKKVHIYMAGDSSGGNIVHHVALKAMESGIEVFGNILLNPLFGGQERTESEKRLDGRYFVGVKDRDWYWRAFLPEGEDRDHHACNPFGPKGKSLEGITFPKSLVVVAGLDLVQDWQLGYAKGLEKAGQEVKLLFLEQATVGFYLLPNNEHFSPVMDEIKYFVGSDC

**>GLYMA_02G151100**

MTGSNEVNLSESKSVVPLNTWVLISNFKLAYNLLRRADGTFNRELAEFLDRKVPANAIPVDGVFSFDHVERSTGLFNRVYQLAPENMGRFIELEKPLSTTEIVPVIIFFHGGSFSHSSANSAIYDTFCRRLVNNCKAVVVSVNYRRSPEYRYPCAYDDGWAALNWVKSRTWLQSGKDSKVHVYLAGDSSGGNIAHHVAVRAAEEDIEVLGNILLHPLFGGEKRTESETKLDGKYFVRLQDRDWYWRAFLPEGTDRDHPACNPFGPKGKNLEGLKFPKSLVCVAGLDLLQDWQVEYVEGLKNCGQDVNLLYLKEATIGFYFLPNNDHFYTLMEEIKNFVNPNC

**>GLYMA_10G022900**

MTESNNEVNLSESKSVVPLNTWVLISNFKLAYNLLRRADGTFNRELAEFLDRKVPANTIPVDGVFSFDHVERSTGLFNRVYQVAPENMGRFIELEKPLSTTKIVPVIIFFHGGSFSHSSANSAIYDIFCRRLVSNCKAVVVSVNYRRSPEYRYPCAYDDGWSALNWVKSRTWLQSGKDSKVHVYLAGDSSGGNIAHHVAVRAAEEDIEVLGNILLHPLFGGEKRTESEMKLDGKYFVRLQDRDWYWRAFLPEGADRDHPACNPFGPKGKNLQGLKLPKSLVCVAGLDLLQDWQLEYVEGLKNCGQDVKLLYLKEATIGFYFLPNNDHFYTLMEEIKNFVNPNC

**>GLYMA_03G148300**

MAGSNQVNLNESRSVVPLNTWVLISNFKLSYKLLRRDDGTFNRELAEYLDRKVPANAIPVEGVFSIDHVDRNAGLFYRVYLPTSGNEAQWGIRDLEKPLSTTEIVPVIVFFHGGSFSHSSANSHIYDTFCRRLVRICKAAVVSVNYRRSPEHRYPCAYDDGWAALRWVKSRAWLQSGREAKVHVYLAGDSSGGNIVHHVAVRAAEEEIEVLGNILLHPLFGGEKRTESELRLDGKYFVRLKDRDWYWRAFLPEGENRDHPACNPFGPRGRSIEGLKFPKSLVCVAGLDLLQDWQLAYAKGLEDCGQQVKLLFLKEATIGFYFLPNNDHFYCLMKEINNFVNSDSDC

**GIBBERELLIC ACID INSENSITIVE （GAI） and REPRESSOR OF GA1-3 （RGA）**

**>AT1G14920 (AtGAI)**

MKRDHHHHHHQDKKTMMMNEEDDGNGMDELLAVLGYKVRSSEMADVAQKLEQLEVMMSNVQEDDLSQLATETVHYNPAELYTWLDSMLTDLNPPSSNAEYDLKAIPGDAILNQFAIDSASSSNQGGGGDTYTTNKRLKCSNGVVETTTATAESTRHVVLVDSQENGVRLVHALLACAEAVQKENLTVAEALVKQIGFLAVSQIGAMRKVATYFAEALARRIYRLSPSQSPIDHSLSDTLQMHFYETCPYLKFAHFTANQAILEAFQGKKRVHVIDFSMSQGLQWPALMQALALRPGGPPVFRLTGIGPPAPDNFDYLHEVGCKLAHLAEAIHVEFEYRGFVANTLADLDASMLELRPSEIESVAVNSVFELHKLLGRPGAIDKVLGVVNQIKPEIFTVVEQESNHNSPIFLDRFTESLHYYSTLFDSLEGVPSGQDKVMSEVYLGKQICNVVACDGPDRVERHETLSQWRNRFGSAGFAAAHIGSNAFKQASMLLALFNGGEGYRVEESDGCLMLGWHTRPLIATSAWKLSTN

**>AT2G01570 (AtRGA)**

MKRDHHQFQGRLSNHGTSSSSSSISKDKMMMVKKEEDGGGNMDDELLAVLGYKVRSSEMAEVALKLEQLETMMSNVQEDGLSHLATDTVHYNPSELYSWLDNMLSELNPPPLPASSNGLDPVLPSPEICGFPASDYDLKVIPGNAIYQFPAIDSSSSSNNQNKRLKSCSSPDSMVTSTSTGTQIGGVIGTTVTTTTTTTTAAGESTRSVILVDSQENGVRLVHALMACAEAIQQNNLTLAEALVKQIGCLAVSQAGAMRKVATYFAEALARRIYRLSPPQNQIDHCLSDTLQMHFYETCPYLKFAHFTANQAILEAFEGKKRVHVIDFSMNQGLQWPALMQALALREGGPPTFRLTGIGPPAPDNSDHLHEVGCKLAQLAEAIHVEFEYRGFVANSLADLDASMLELRPSDTEAVAVNSVFELHKLLGRPGGIEKVLGVVKQIKPVIFTVVEQESNHNGPVFLDRFTESLHYYSTLFDSLEGVPNSQDKVMSEVYLGKQICNLVACEGPDRVERHETLSQWGNRFGSSGLAPAHLGSNAFKQASMLLSVFNSGQGYRVEESNGCLMLGWHTRPLITTSAWKLSTAAY

**>BnaA06g34810D**

MKRDLHQFQGPNHGTSIAGSSTSSPAVFGKDKMMMVKEEEDDELLGVLGYKVRSSEMAEVALKLEQLETMMGNAQEDGLAHLATDTVHYNPAELYSWLDNMLTELNPPAATTGSNALNPEINNNNNNSFFTGGDLKAIPGNAVCRRSNQFAFAVDSSSNKRLKPSSSPDSMVTSPSPAGVIGTTVTTVTESTRPLILVDSQDNGVRLVHALMACAEAVQSSNLTLAEALVKQIGFLAVSQAGAMRKVATYFAEALARRIYRLSPPQTQIDHSLSDTLQMHFYETCPYLKFAHFTANQAILEAFEGKKRVHVIDFSMNQGLQWPALMQALALREGGPPSFRLTGIGPPAADNSDHLHEVGCKLAQLAEAIHVEFEYRGFVANSLADLDASMLELRPSETEAVAVNSVFELHKLLGRTGGIEKVFGVVKQIKPVIFTVVEQESNHNGPVFLDRFTESLHYYSTLFDSLEGAPSSQDKVMSEVYLGKQICNLVACEGPDRVERHETLSQWSNRFGSSGFAPAHLGSNAFKQASTLLALFNGGEGYRVEKNNGCLMLSWHTRPLITTSAWKLSAVH

**>BnaA09g18700D**

MKRDLHQFQGPPDTRFPNHGTANTGSSSKDKMMMVKEEEDGGNMDELLAVLGYKVRSSEMAEVALKLEQLETMMGNVQEDGLSNLATDTVHYNPSELYSWLDNMLTEFNPPPPEINNSFLAGAGGSDYDLKAIPGNAIYARSDQFAIDSSSSSNQAGDNSQSTKRLKSCSSPDSLSTRSSTRSMVLVDSQENGVRLVHALMACAEAIQNNDLSIAEALVKQIGFLAVSQAGAMRKVATYFAEALARRIYRLSPPQTQIDHSLSDTLQMHFYETCPYLKFAHFTANQAILEAFEGKKRVHVIDFSMNQGLQWPALMQALALREGGPPVFRLTGIGPPAADNSDHLHEVGCKLAQLAEAIHVEFEYRGFVANSLADLDASMLELRPSEIEAVAVNSVFELHKLLGRTGGIEKVLGVVKQIKPVIFTVVEQESSHNGPDFLDRFTESLHYYSTLFDSLEGVPSSQDKVMSEVYLGKQICNLVACEGPDRVERHETLSQWANRFGTSGFAPAHLGKRVCFWLCLTAAKVIVWRRIMGA

**>Csa_5G569350**

MKREHHHLHPRPEPPSMAVVPNGESFLNTGKAKLWEEEAQLDGGMDELLAVLGYKVKSSDMADVAQKLEQLEEAMCQVQDTGLSHLAFDTVHYNPSDLSTWLESMLTELHPMPNFATPPPPSQLDDPSFLAPAESSTITSIDYDPQRQTSSRIFEESSSSDYDLKAITSSAIYSPRENKRLKSSESDSDVFSTSAIRASDSVTRPVVLVDSQENGIQLVHALMACAEAVQQNNLNIAEALVKRIGYLAVSQAGAMRKVATFFAEALARRIYRLCPENPLDHSVSDRLQMHFYESCPYLKFAHFTANQAILEAFEGKKRVHVIDFSMNRGMQWPALIQALALRPNGPPAFRLTGIGPPAPDNSDYLQEVGWKLAELAEAIHVDFEYRGFVANSLADLDASMLELRPSEVESVVVNSVFELHKLLARPGALEKVLSVVKQMKPEIMTVVEQEANHNGPVFVDRFTESLHYYSTLFDSLEGSPNNQDKIMSEMYLGKQICNVVACEGADRVERHETLTQWQTRLSSAGFEPIHLGSNAFKQASMLLALFGSGEGYRVEENNGSLMLGWHTRPLIATSAWKIGNNPVVAK

**>GLYMA_05G140400**

MKRERQQLGSNAGTSSCGYSSGKSNLWEEEGGMDELLAVVGYKVRSSDMAEVAQKLERLEEAMGNVQDDLTDLSNDAVHYNPSDISNWLQTMLSNFDPLPSEEPEKDSASSDYDLKAIPGKAIYGGGSDALPNPKRVRTDESTRAVVVVDLQENGIRLVHSLMACAEAVENNNLAVAEALVKQIGFLALSQVGAMRKVATYFAEALARRIYRVFPQQHSLSDSLQIHFYETCPYLKFAHFTANQAILEAFQGKNRVHVIDFGINQGMQWPALMQALALRNDGPPVFRLTGIGPPAADNSDHLQEVGWKLAQLAERIHVQFEYRGFVANSLADLDASMLDLREDESVAVNSVFEFHKLLARPGAVEKVLSVVRQIRPEILTVVEQEANHNGLSFVDRFTESLHYYSTLFDSLEGSPVNPNDKAMSEVYLGKQICNVVACEGMDRVERHETLNQWRNRFGSTGFSPVHLGSNAYKQASMLLSLFGGGDGYRVEENNGCLMLGWHTRPLIATSVWQLATKSVVAAH

**>GLYMA_08G095800**

MKREREQLGSIAGTSSCGYSSGKSNLWEEEGGMDELLAVVGYKVRSSDMAEVAQKLERLEEAMGNVQDDLPEISNDVVHYNPSDISNWLETMLSNFDPLPSEEPEKDSASSDYDLKAIPGKAIYGASDALPNPKRVKADESRRAVVVVDSQENGIRLVHSLMACAEAVENNNLAVAEALVKQIGFLAVSQVGAMRKVAIYFAEALARRIYRVFPLQHSLSDSLQIHFYETCPYLKFAHFTANQVILEAFQGKNRVHVIDFGINQGMQWPALMQALAVRTGGPPVFRLTGIGPPAADNSDHLQEVGWKLAQLAEEINVQFEYRGFVANSLADLDASMLDLREGEAVAVNSVFEFHKLLARPGAVEKVLSVVRQIRPEIVTVVEQEANHNRLSFVDRFTESLHYYSTLFDSLEGSPVNPNDKAMSEVYLGKQICNVVACEGMDRVERHETLNQWRNRFVSTGFSSVHLGSNAYKQASMLLALFAGGDGYRVEENNGCLMLGWHTRPLIATSAWQLAATR

**BRASSINOSTEROID INSENSITIVE 2 (BIN2)**

**>AT4G18710 (AtBIN2)**

MTSDGATSTSAAAAAAAAAAARRKPSWRERENNRRRERRRRAVAAKIYTGLRAQGDYNLPKHCDNNEVLKALCVEAGWVVEEDGTTYRKGCKPLPGEIAGTSSRVTPYSSQNQSPLSSAFQSPIPSYQVSPSSSSFPSPSRGEPNNNMSSTFFPFLRNGGIPSSLPSLRISNSCPVTPPVSSPTSKNPKPLPNWESIAKQSMAIAKQSMASFNYPFYAVSAPASPTHRHQFHTPATIPECDESDSSTVDSGHWISFQKFAQQQPFSASMVPTSPTFNLVKPAPQQMSPNTAAFQEIGQSSEFKFENSQVKPWEGERIHDVGMEDLELTLGNGKARG

**>BnaA02g17120D**

MTWDGDTSTSAGAARRERENNRRRCVAAKIYTGLRAQGDFNLPKHCDNNEVLKALSLF

**>Csa_6G501930**

MTGRGSSGRTPTWKERENNKRRERRRRAIAAKIYTGLRAQGNYKLPKHCDNNEVLKALCNEAGWVVEEDGTTYRKGCKPPPIDIGTSANMSACSSLQPSPQSSCFPSPVPSYHASPSSSSFPSPTRFDGNPSSYLLPFLQNISSIPANLPPLRISNSAPVTPPLSSPTSRGSKRKPDWESIPNSYVTSFRHPLFAVSAPSSPTRCHHLTPATIPECDESDASTVDSGRWVSFQTVAPSVAPPSPTFNLMKPVSQQNSLQDAVDRHGAMGWGATSDRGRGSEFEFEKFESGTVKPWEGERIHEVGVDDLELTLGGGKARG

**>GLYMA_12G231400**

MTSGARQPTWKERENNKRRERRRRAIAAKIFSGLRMYGNYKLPKHCDNNEVLKALCNEAGWTVEADGTTYRKGCKPPVERMDIVGGSAAASPCSSYHPSPCASYNPSPGSSCLPSPRASPYPPNHNADGNSLIPWLKNLSSGSSSASSSKLPQLYIPNGSISAPVTPPISSPSSRKPRINADWEDLSTRPAAWGGPAYTFLPSSTPPSPGRQVAETDWFSKIRIPQVGLTPTSPTFSLVSSNPFGFKEDAMGGSGSRMWTTPGASGTCSPAVAAGSENTSDIPMAEAVSDEFAFGSSSSVLVNAWKGERIHEASFGTDDLELTLGSSKTRLLHK

**>GLYMA_13G266600**

MTSVARQPTWKERENNKRRERRRRAIAAKIFSGLRMYGNYKLPKHCDNNEVLKALCNEAGWTVEADGTTYRKGCKPPVERMDIVGGSAAASPCSSYHPSPCASYNPSPGSSCLPSPRASPFPPNPNADGNSLIPWLKNLSSGSSSASSSKLPQLYIPNGSISAPVTPPISSPSSRKPQIRADWEDQSNCPTAWGGPAYTFVPSSTPPSPGRQVAETDWFSKIRIPQGGLAPTSPTFSLVSSNPFGLKEDAMVGSGSRMWTTPGASGTCSPAVAAGSENTSDIPMAEAVSDEFAFGSSSSGLVNAWKGERIHEASFGTDDLELTLGSSKTRLLHK

**BRASSINAZOLE-RESISTANT 1 (BZR1) and BRI1-EMS-RESISTANT 1 (BES1)**

**>AT1G19350 (AtBES1)**

MKRFFYNSSEEERKKKAYSSKKMTSDGATSTSAAAAAAAMATRRKPSWRERENNRRRERRRRAVAAKIYTGLRAQGNYNLPKHCDNNEVLKALCSEAGWVVEEDGTTYRKGHKPLPGDMAGSSSRATPYSSHNQSPLSSTFDSPILSYQVSPSSSSFPSPSRVGDPHNISTIFPFLRNGGIPSSLPPLRISNSAPVTPPVSSPTSRNPKPLPTWESFTKQSMSMAAKQSMTSLNYPFYAVSAPASPTHHRQFHAPATIPECDESDSSTVDSGHWISFQKFAQQQPFSASMVPTSPTFNLVKPAPQQLSPNTAAIQEIGQSSEFKFENSQVKPWEGERIHDVAMEDLELTLGNGKAHS

**>AT1G75080 (AtBZR1)**

MTSDGATSTSAAAAAAAAAAARRKPSWRERENNRRRERRRRAVAAKIYTGLRAQGDYNLPKHCDNNEVLKALCVEAGWVVEEDGTTYRKGCKPLPGEIAGTSSRVTPYSSQNQSPLSSAFQSPIPSYQVSPSSSSFPSPSRGEPNNNMSSTFFPFLRNGGIPSSLPSLRISNSCPVTPPVSSPTSKNPKPLPNWESIAKQSMAIAKQSMASFNYPFYAVSAPASPTHRHQFHTPATIPECDESDSSTVDSGHWISFQKFAQQQPFSASMVPTSPTFNLVKPAPQQMSPNTAAFQEIGQSSEFKFENSQVKPWEGERIHDVGMEDLELTLGNGKARG

**>BnaA09g44210D**

MKHVTGAQPAQWLEVFAKEMEAGWIRDTSYKTSGVHQYVLAKRILGPNFMVGSEDSEETFGKHQKKNMTSEGAMLTSAAAMATRRKPSWRERENNRRRERKRRAVAAKIYNGLRAQGNFNLPRHCDNNEVLKALCSEAGWVVEEDGTTYRKGHKPLPDDMAGSSSRATAYSSYNQSPFESPILSYQASPSSSSFPSPRGGDTHNISTIFPFLRNGGINPSSLPPLRISNSAPVTPPVSSPTSRNPKPLPTWESLTKQAMANAARQSVSSFNYPFYAVSAPTSPTHHRQFNAPATIPECDESDSSTTDSGHWISFQKFSQQQFRGGFAVPPSPTFNLVKPPLPQQLSSNIAAAQEIGSQVKPWEGERIHDVAMEDLELTLGNSKGRS

**>BnaA08g22080D**

MTSDGATSTSAAAAMAATRRKPSWRERENNRRRERRRRAVAAKIYTGLRAQGNYNLPKHCDNNEVLKALCSEAGWVVEEDGTTYRKGHKPPSLPGDVAGSSSRATPYSSYNQSPFESPILSYQVSPSSSSFPSPSRGGGDTHNNISTIFPFLRNGGIPSSLPPLRISNSAPVTPPVSSPSSKHPKTLPTWGECFTNQSAKQSMSSFNYPFYAVSAPASPTHHRQFNAPPATIPECDESDASTVDSGHWISFQKFSQQQPFLGVSAVPASPTFNLVRPPVPQQLSPNTGATQEIGQSSEFKFENSQVKPWEGERIHDVAMEDLELTLGNAKGRM

**>Csa_2G361450**

MTSDGATSASNCRRKPSWRERENNRTRERRRRAIAAKIYSGLRAQGNFNLPKHCDNNEVLKALCAEAGWTVEDDGTTYRKGCKPPPIDIVGTSTKITPYSSQNPSPLSSSFPSPMGSYQVSPSSSSFPSPSRYDANNPSNLIPYLRQAIPISLPPLRISNSAPVTPPLSSPASRTPQPFPNWEVNAKESLSSLNYPFFAVSAPASPTRPQLHTPAPIHKCDESESSTNDSNQWALFRAYAPSASTMPTSPTFNLVKPADQHVLHGGFIQENGRRNEFEFLGYKVKPWEGEKIHDVGLEDLELTLGSSKARS

**>GLYMA_14G076900**

MVDDGATSAATSRRKPSWRERENNRRRERRRRAIAAKIYSGLRAQGNFNLPKHCDNNEVL

KALCAEAGWCVEEDGTTYRKGCKPPLANGAGSSMRNITFSSSQNPSPLSSSFPSPIPSYQVSPSSSSFPSPFRLDVDKDNVSHLIPYIRNASLSLPPLRISNSAPVTPPLSSPTSRNPKPIPTWESIAKESMASFSYPFFAASAPASPTHRHLYTPPTIPECDESDTSTGESGQWVKFQAFAPSSSVLPISPTFNLVKPVVPPGMPDNSIQEMRTSSDEFGVQVKPWVGEKIHEVALDDLELTLGSGKVRS

**>GLYMA_17G248900**

MADDGATSAATSRRKPSWRERENNRRRERRRRAIAAKIYSGLRAQGNFNLPKHCDNNEVLKALCAEAGWCVEEDGTTYRKGCKPPLANGAGSSMRNIPFSSSQNPSPLSSSFPSPIPSYQVSPSSSSLPSPFRLDGDKDNVSNLIPYIRNASLSLPPLRISNSAPVTPPLSSPTSRNSKPIPTWESIAKESMASFNYPFFAASAPASPTHRHLYTPLTIPECDESDTSIGESGQWVKFQAFAPSASVFPTSPTFNLVKPVIPHRMPDNSIQVMRTSSEEFGVQVKPWVGEKIHEVALDDLELTLGSGKVRS
